# Supplementary material for: Elucidating target specificity of the taccalonolide covalent microtubule stabilizers employing a combinatorial chemical approach
Source: Nat Commun. 2020 Jan 31;11:654. doi: 10.1038/s41467-019-14277-w (PMC6994698; doi:10.1038/s41467-019-14277-w)
Supplement: Supplementary file 1 — Supplementary Information [file 41467_2019_14277_MOESM1_ESM.pdf]

Supplementary Information

Elucidating target specificity of the taccalonolide covalent microtubule stabilizers employing a combinatorial chemical approach

Lin Du et al.

Contents:

Supplementary Figures 1-96
Supplementary Tables 1-11
Supplementary Methods
Supplementary References

Supplementary Figures

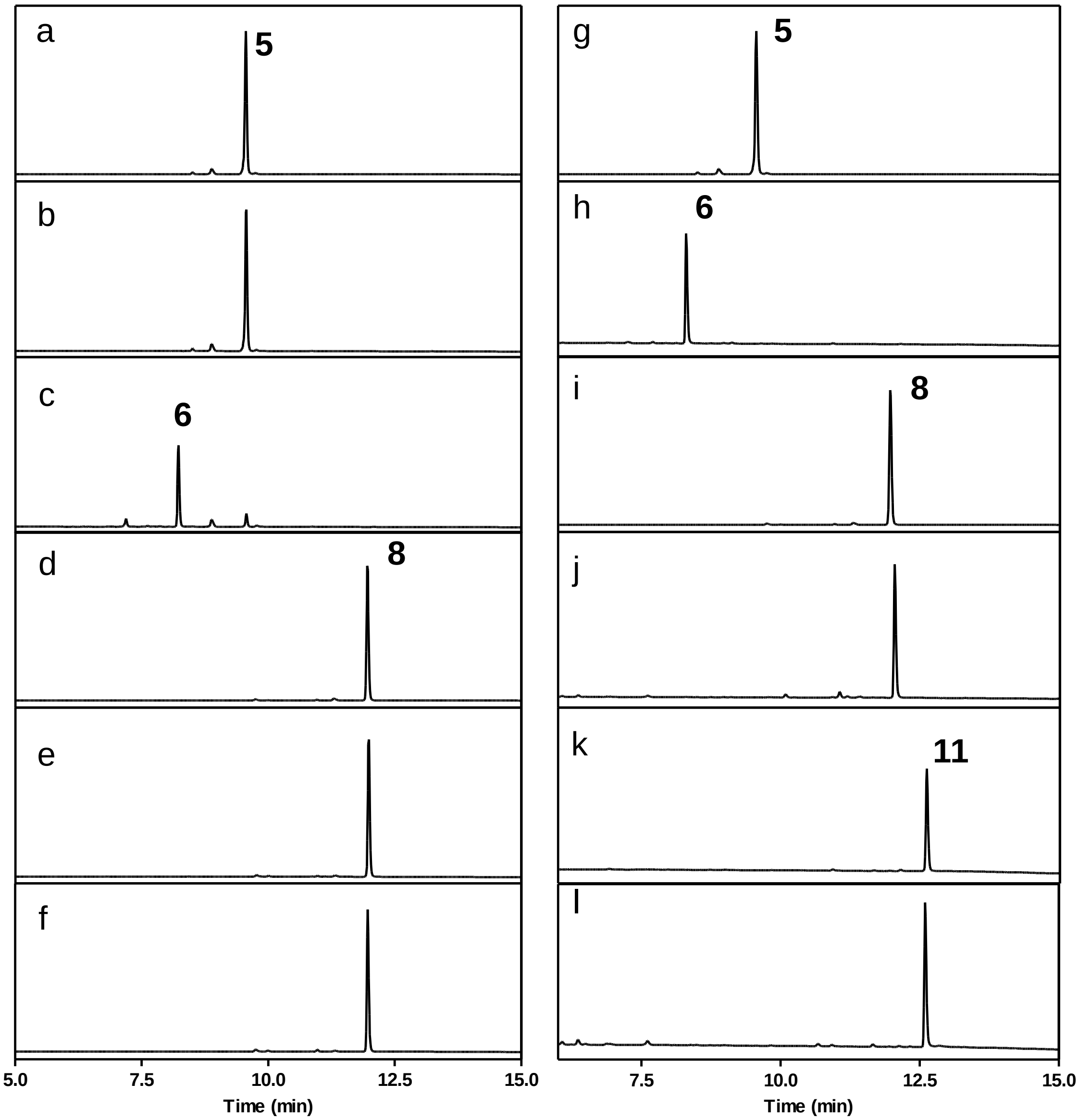

Supplementary Figure 1. Hydrolytic stability of taccalonolide-based probes. The MeOH solutions of pure **5** (**a**) and **8** (**d**) were analyzed by LCMS to provide the controls. The MeOH [(**b**) and (**e**)] and 50% MeOH/PBS solutions [(**c**) and (**f**)] of **5** and **8** were kept overnight in static followed by LCMS analysis. (**g**)-(**l**) Hydrolytic stability of **5**, **8**, and **11** in complete RPMI 1640 medium with 10% FBS. The MeOH solutions of pure **5** (**g**), **8** (**i**), and **11** (**k**) were analyzed by LCMS to provide the controls. Each compound (100 µM) was incubated in 0.1 mL RPMI 1640 medium at 37 ºC, respectively, for 1 hr (**5**, **h**) or 16 hrs (**8**, **j**, and **11**, **l**) in static.

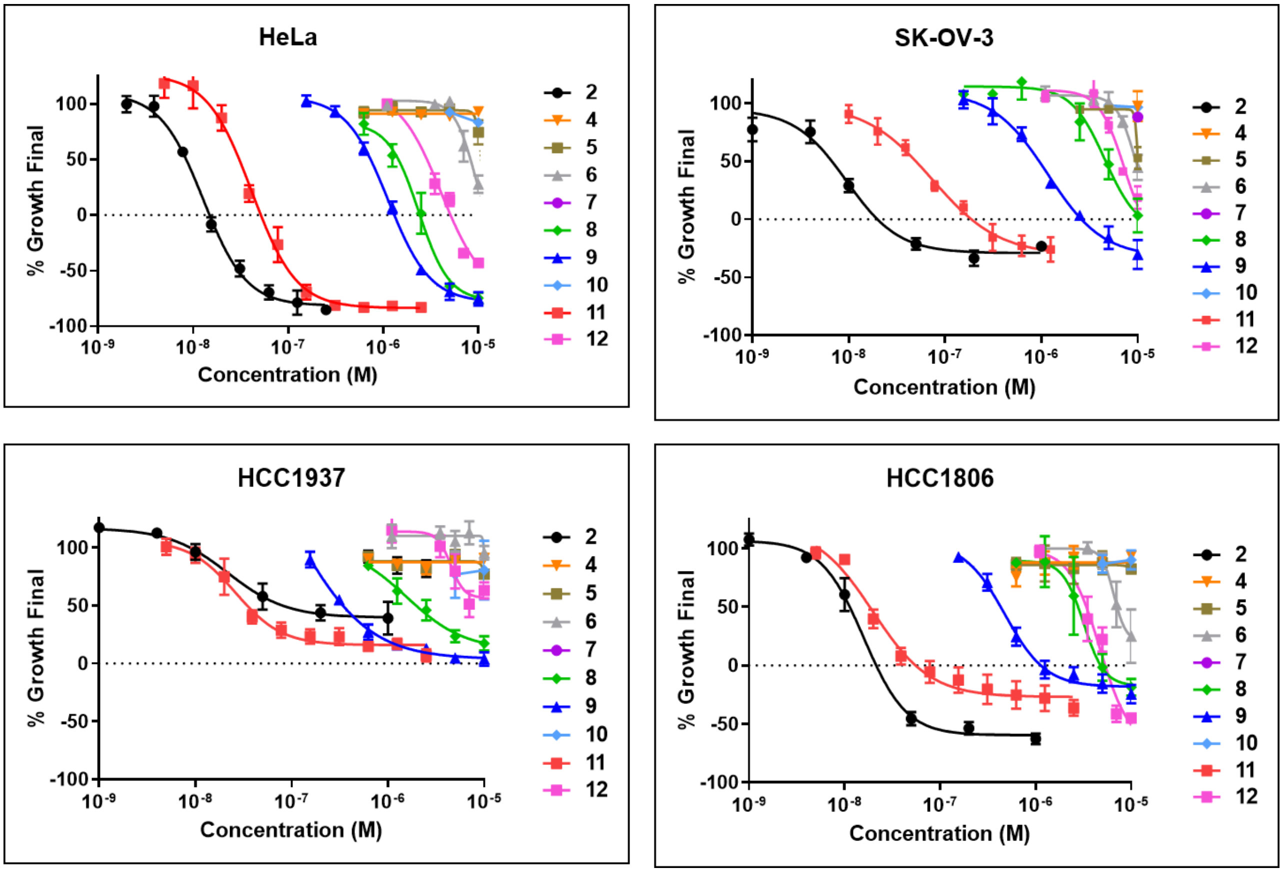

Supplementary Figure 2. Concentration-response curves for the growth of cancer cells treated with taccalonolide or taccalonolide probes. For each graph, **2** (black circle), **4** (orange upside-down triangle), **5** (brown square), **6** (grey triangle), **7** (purple circle), **8** (green diamond), **9** (dark blue triangle), **10** (light blue diamond), **11** (red square), and **12** (pink square). All points are from 3 biologically independent experiments, each performed in triplicate represented as mean ± SEM with the exception of the following: n = 4 for **5**, **6**, **8**, **11**, and **12** in SK-OV-3, n = 2 for **10** in HeLa and HCC1937. Source data are provided as a Source Data file.

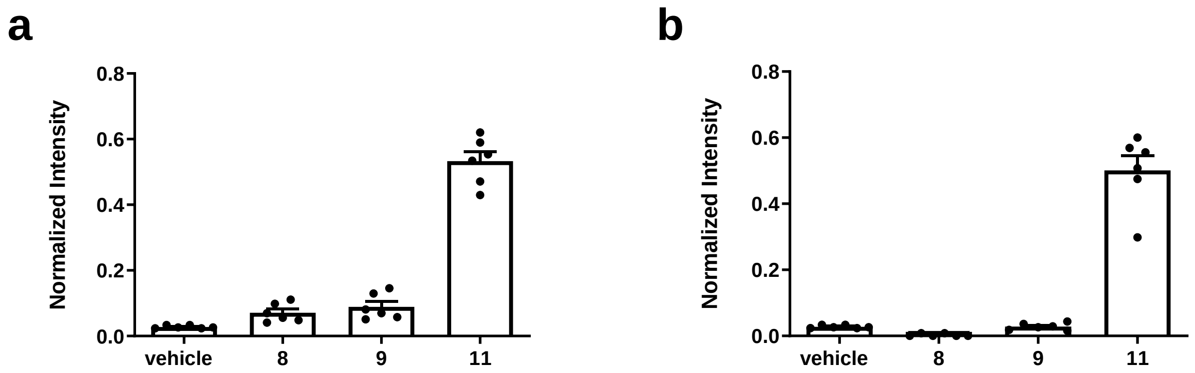

Supplementary Figure 3. Quantification of fluorescent intensity of taccalonolide probes. Intensity values from Figure 4c (**a**) and 4d (**b**) were obtained from 6 independent wells, normalized on a log scale to the weakest signal, and presented as mean ± SEM. Source data are provided as a Source Data file.

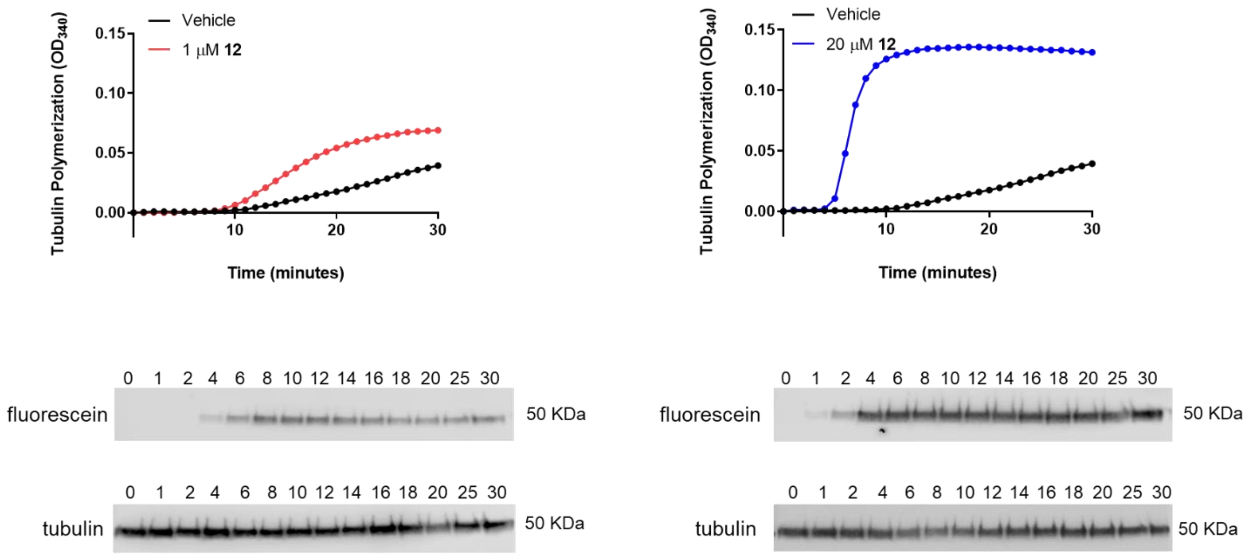

Supplementary Figure 4. Time course of microtubule polymerization and the binding of taccalonolide probe **12** to purified tubulin. Pure tubulin at a concentration of 20 µM was incubated with vehicle (black) or **12** at a concentration of either 1(red) or 20 µM (blue). The extent of microtubule polymerization or **12** binding was determined by the extent of fluorescein signal as compared to tubulin signal by immunoblotting at the indicated times after warming the samples to 37˚C. Data are representative of two independent experiments. Source data are provided as a Source Data file.

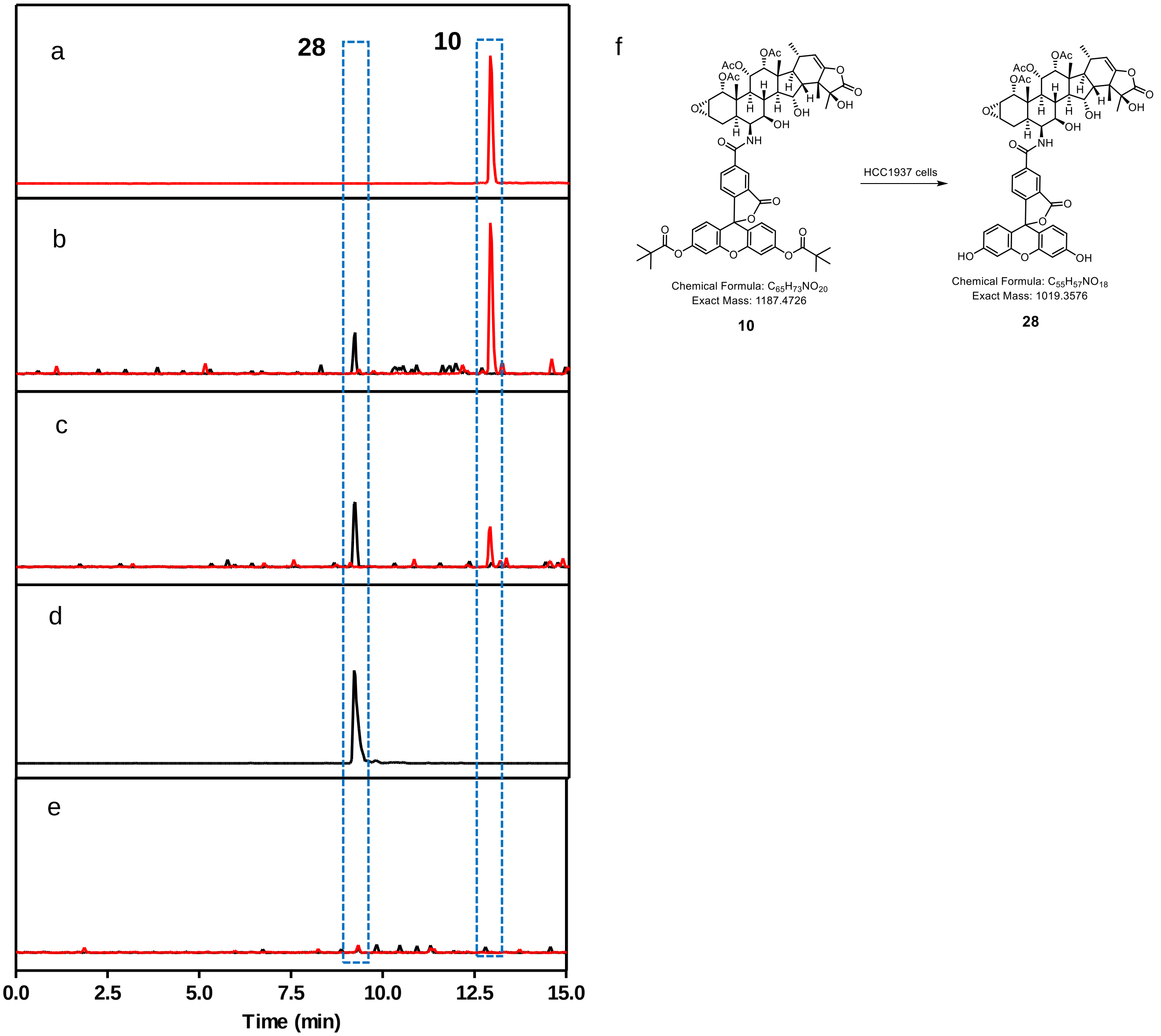

Supplementary Figure 5. Cellular hydrolysis and deprotection of **10**. Compound **10** prior to addition to cells (**a**) or after addition to HCC1937 cells at 1 μM for 8 h (**b**) and (**c**), biological replicates. Black trace: negative mode, selected ion at *m/z* 1018.35 (**28**); red trace: negative mode, selected ion at *m/z* 1186.46 (**10**). The LCMS trace of the **28** alone is shown in (**d**) and a control for cells treated with vehicle is in (**e**). Intracellular compounds were detected after the removal of medium and PBS wash 2x prior to lysis, extraction with EtOAc, and redissolving the dried extracts in 100 μM MeOH followed by LCMS analysis. (**f**) Structures of **10** and its deprotected analogue **28**.

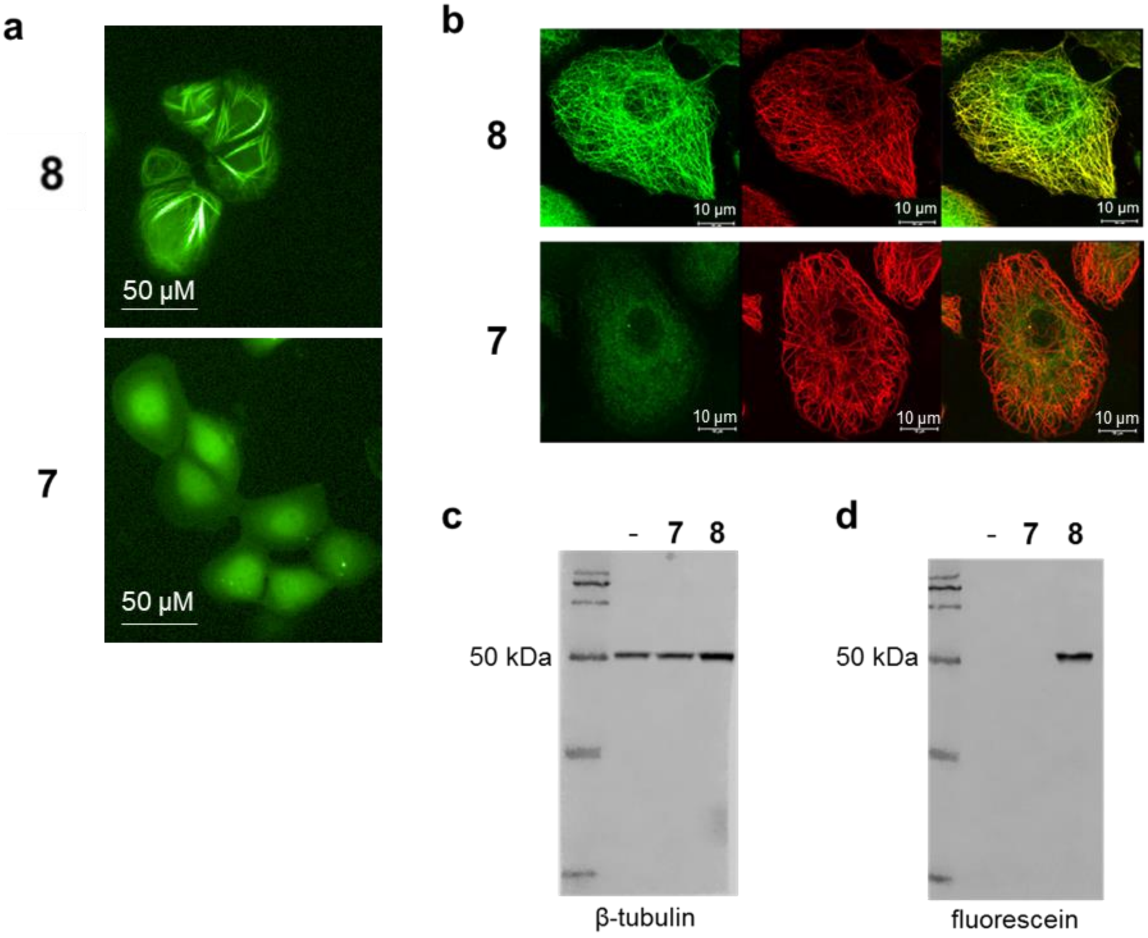

Supplementary Figure 6. The taccalonolide C-22 epoxide is critical for the localization and binding of taccalonolides to β-tubulin. HCC1937 cells were treated with 5 μM taccalonolide probes with (**8**) or without (**7**) the 22,23-epoxide for 6 h. (**a**) Visualization of taccalonolide probes **8** and **7** in live HCC1937 cells. (**b**) Confocal imaging-based colocalization of taccalonolide probe **8** (green) with β-tubulin (red) immunofluorescence in fixed HCC1937 cells as compared to **7**. (**c,d**) HCC1937 cells treated with **7** or **8** were lysed and subjected to immunoblotting using an anti-β-tubulin antibody (**c**) or an anti-fluorescein antibody (**d**). Source data are provided as a Source Data file.

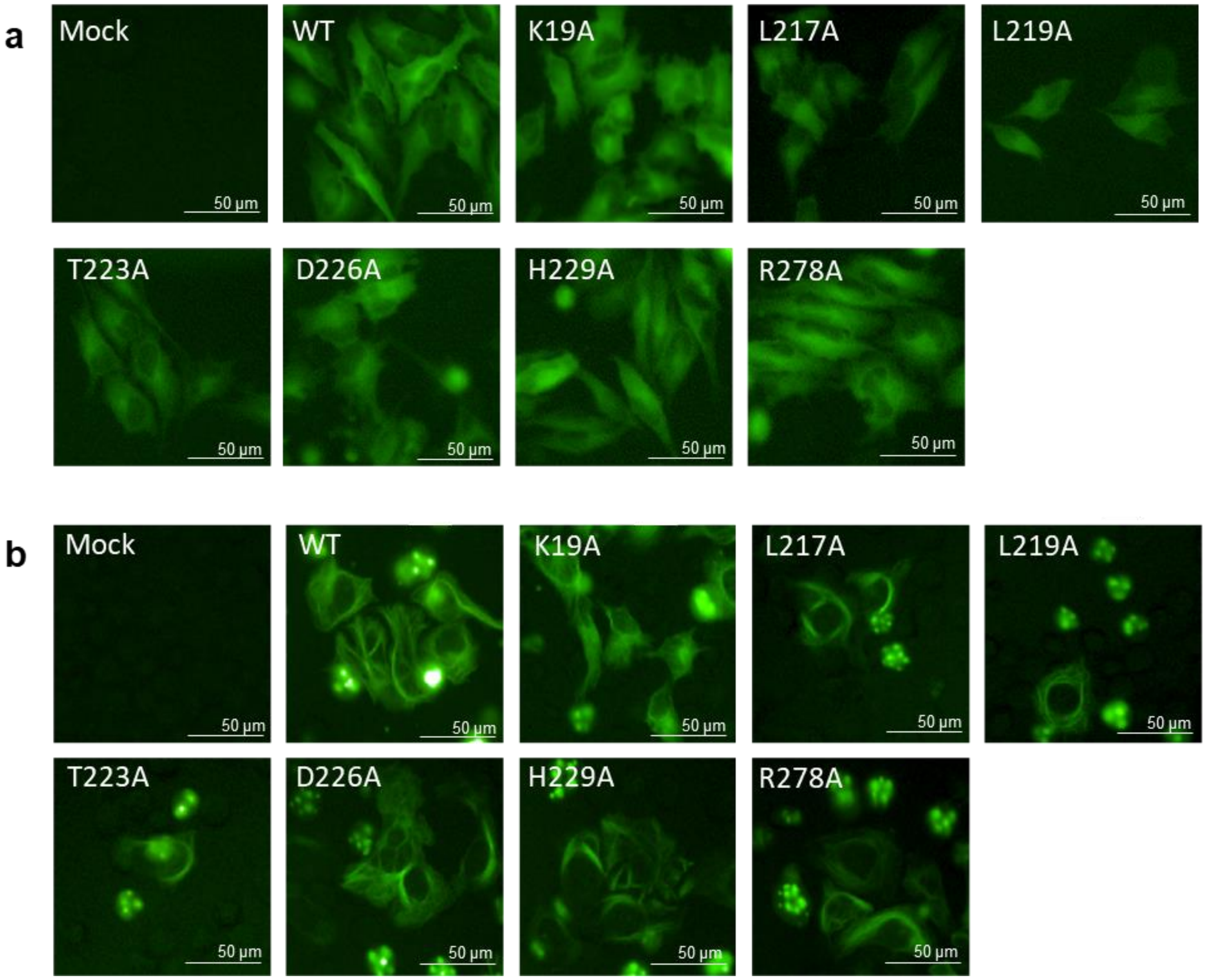

Supplementary Figure 7. The incorporation of GFP-tagged β-tubulin mutants into microtubules. GFP-tagged mutants were visualized in live HeLa cells before (**a**) and after (**b**) treatment with 100 nM **2** for 22 h. Source data are provided as a Source Data file.

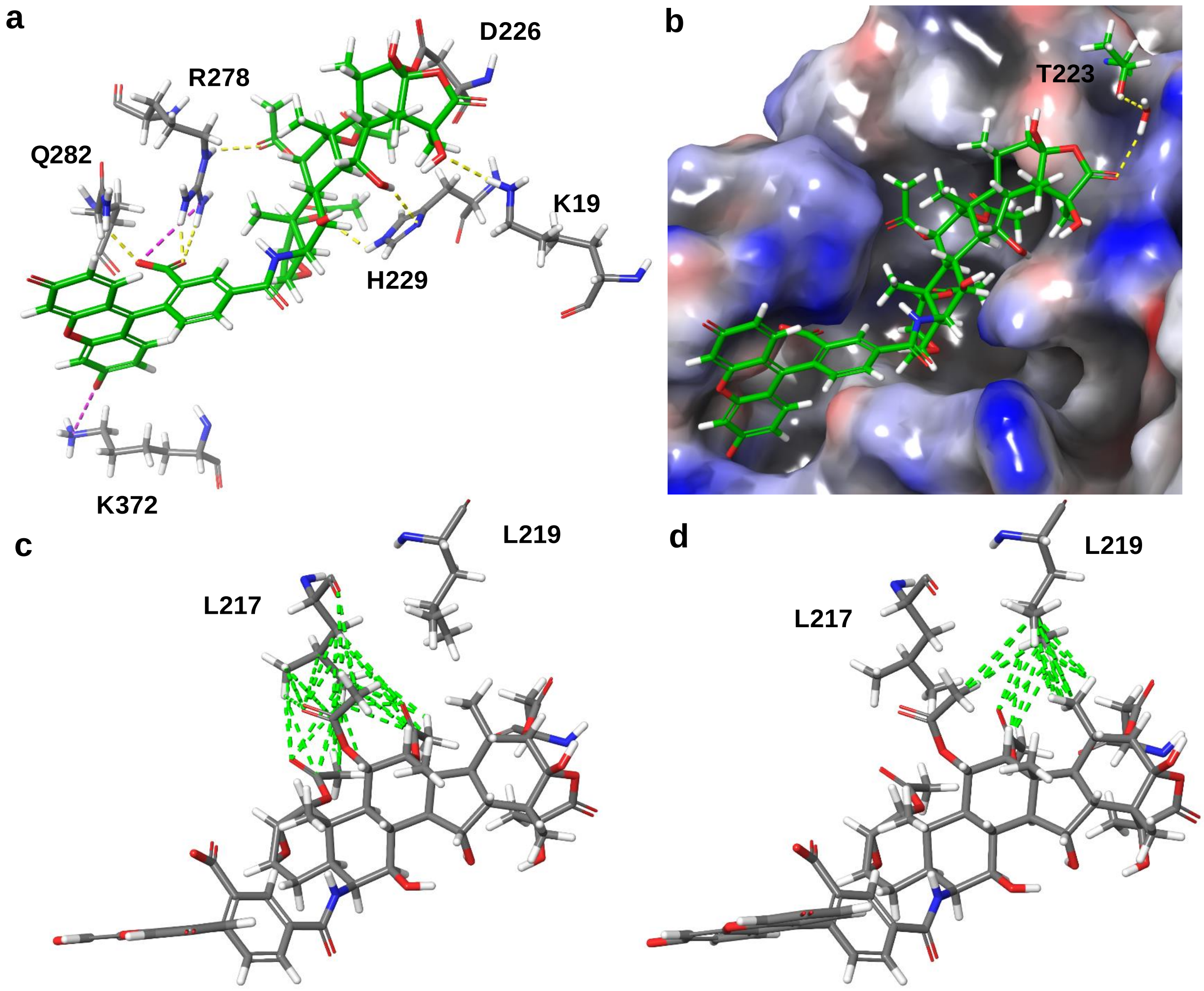

Supplementary Figure 8. Non-covalent interactions between selected β-tubulin residues and **12**. (**a**) Five β-tubulin residues (R278, Q282, K19, H229, and K372) directly interact with **12** via H-bonds and salt bridges; (**b**) T223 indirectly interacts with **12** via H_2O-bridged H-bonds; (**c**) Hydrophobic interactions between L217 and **12**; (**d**) Hydrophobic interactions between L219 and **12**. The selected H-bonds, hydrophobic interactions, and salt bridges are displayed as yellow, green, and magenta dashed lines, respectively.

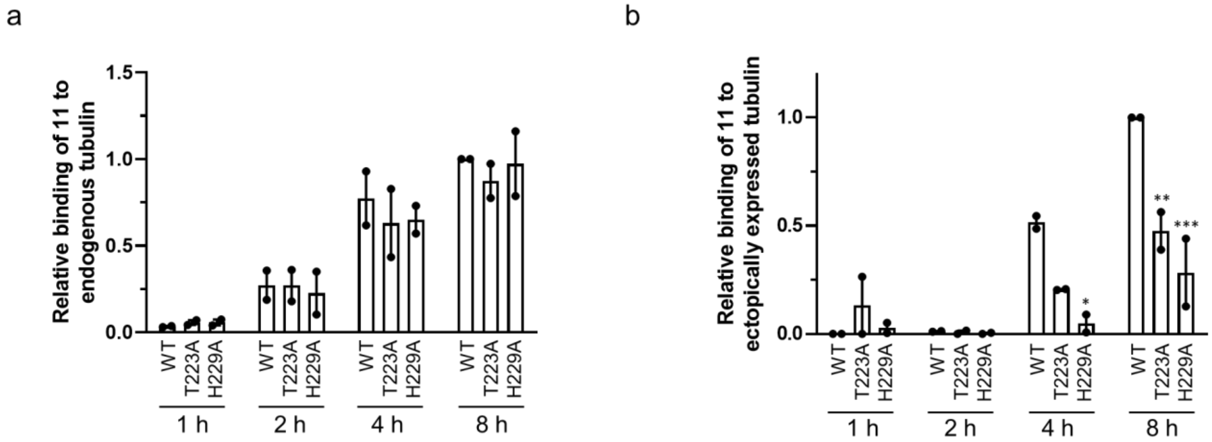

Supplementary Figure 9. Time course of **11** binding to wild type (WT) or mutant β-tubulin. HeLa cells were transfected with GFP-tagged TUBB1 constructs with indicated mutations then treated with 1 μM **11** for 1 - 8 h. Probe-treated cells were lysed and subjected to immunoblotting for fluorescein or β-tubulin. Ratio of **11** bound to endogenously expressed tubulin (**a**) or ectopically expressed tubulin constructs (**b**), mutant or WT, was normalized to the ratio of the WT form bound at 8 h. Average ± SEM for n = 2 independent experiments. One-way ANOVA and Tukey's post-hoc test were used to calculate statistical significance between each condition and significant differences between the binding of **11** to the mutants at each time point as compared to wild type control are depicted:*p<0.05, **p<0.01, ***p<0.001. Source data are provided as a Source Data file.

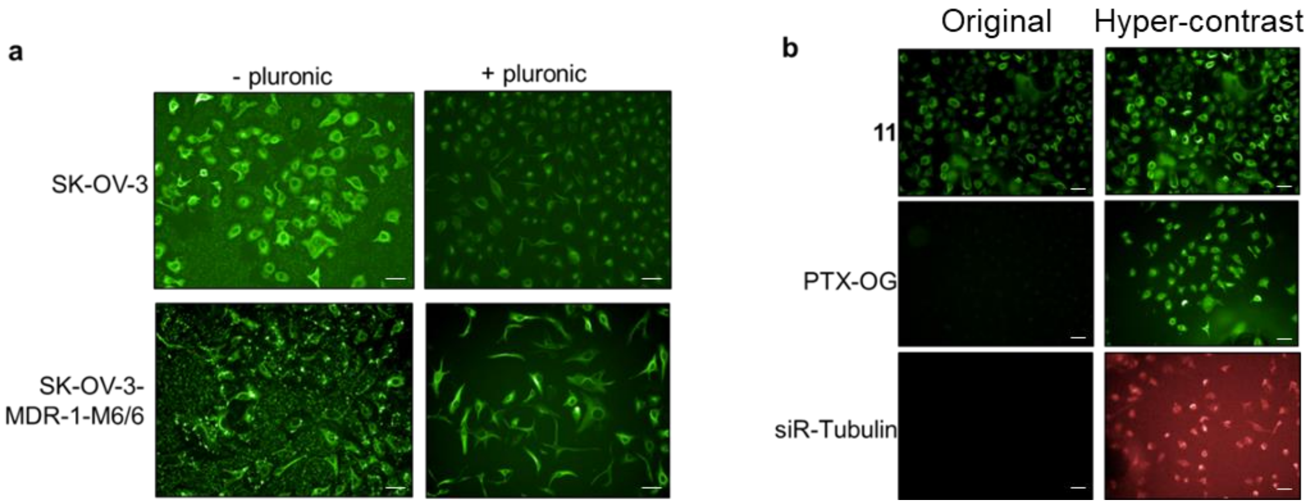

Supplementary Figure 10. Effect of pluronic F-127 on probe imaging. (**a**) Comparison of Tubulin tracker green in SK-OV-3 or SK-OV-3-MDR-1-M6/6 cells with or without pluronic F-127 which facilitates probe loading and reduces background signal. (**b**) Images of chilled SK-OV-3 cells from Figure 7 were hyper-contrasted to visualize low signal intensity. Scale bars = 50 μm. Source data are provided as a Source Data file.

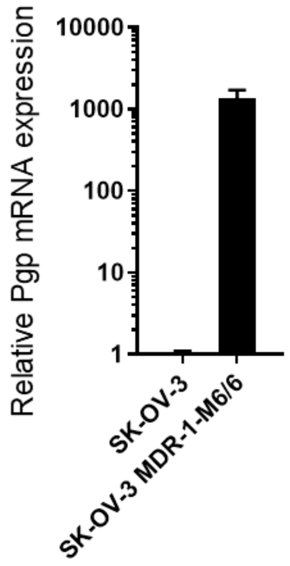

Supplementary Figure 11. P-glycoprotein (Pgp) expression. Levels of Pgp mRNA are over 1000 x greater in the SK-OV-3 MDR-1-M6/6 cells than the parental SK-OV-3 cells. A one-tailed t-test was performed to give a P value of 0.0307 between the SK-OV-3 MDR-1-M6/6 cell line and the parental SK-OV-3 cells for Pgp expression where mean relative Pgp mRNA expression ± SEM for n = 2 biologically independent experiments each performed in duplicate. Source data are provided as a Source Data file.

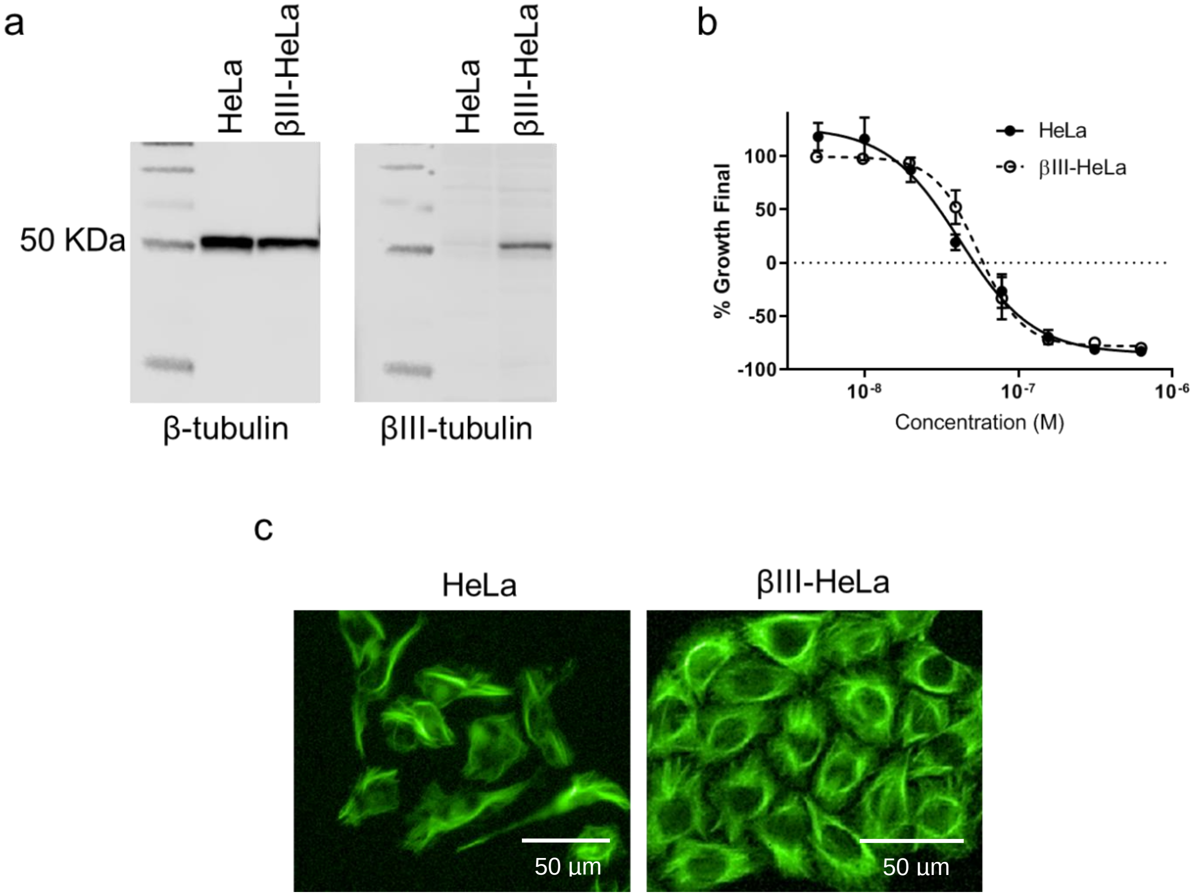

Supplementary Figure 12. Taccalonolide probes retain potency and efficacy in βIII-tubulin expressing cells. (**a**) Expression of total β-tubulin and the βIII-isotype of tubulin in HeLa cells and an isogenic line that overexpresses this isotype (βIII-HeLa). (**b**) The taccalonolide probe **11** retains antiproliferative and cytotoxic potency and efficacy in the βIII-tubulin expressing cell line (open circle) as compared to the parental HeLa cell line (closed circle). Each point represents mean ± SEM from n = 4 biologically independent experiments for βIII-HeLa cells and n = 3 independent experiments for HeLa cells. (**c**) The taccalonolide probe **11** retains the ability to bind cellular microtubules in the βIII-tubulin expressing cell line as compared to the parental HeLa cell line. Cells were treated with 0.5 μM **11** for 5 h and imaged under identical acquisition conditions. Source data are provided as a Source Data file.

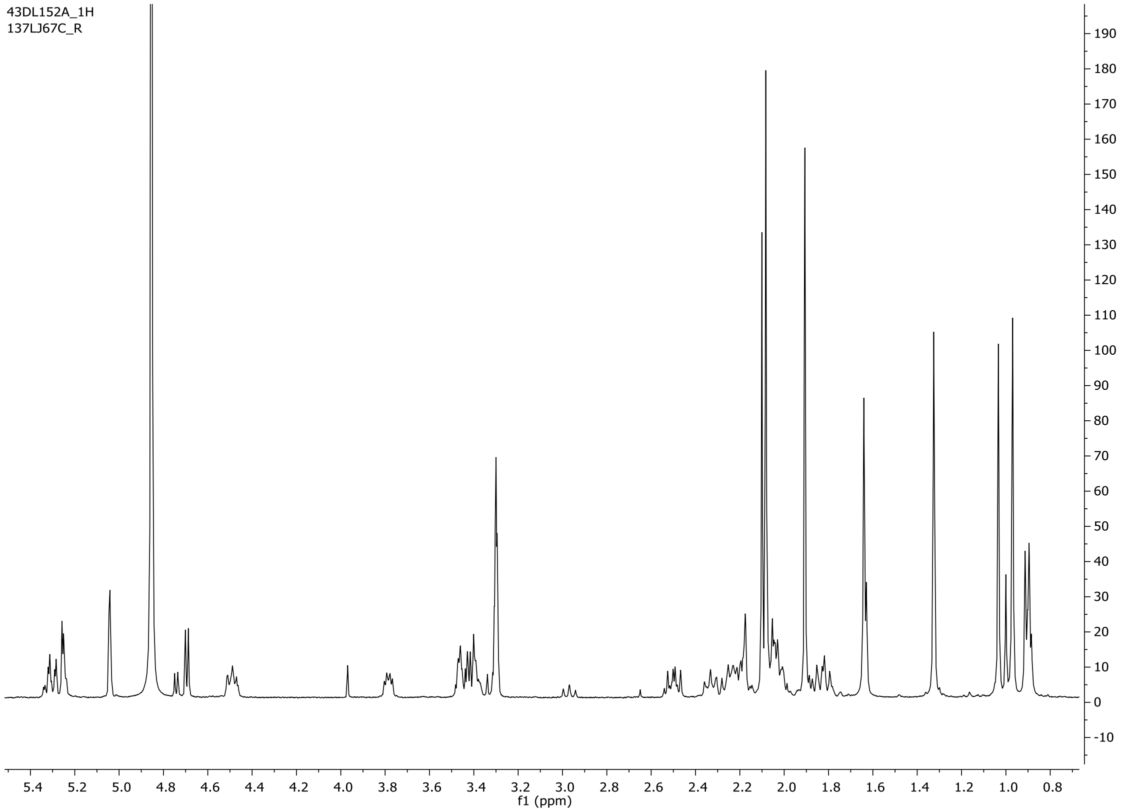

Supplementary Figure 13. ^{1}H NMR spectrum of **TFA-14** in methanol-d_4

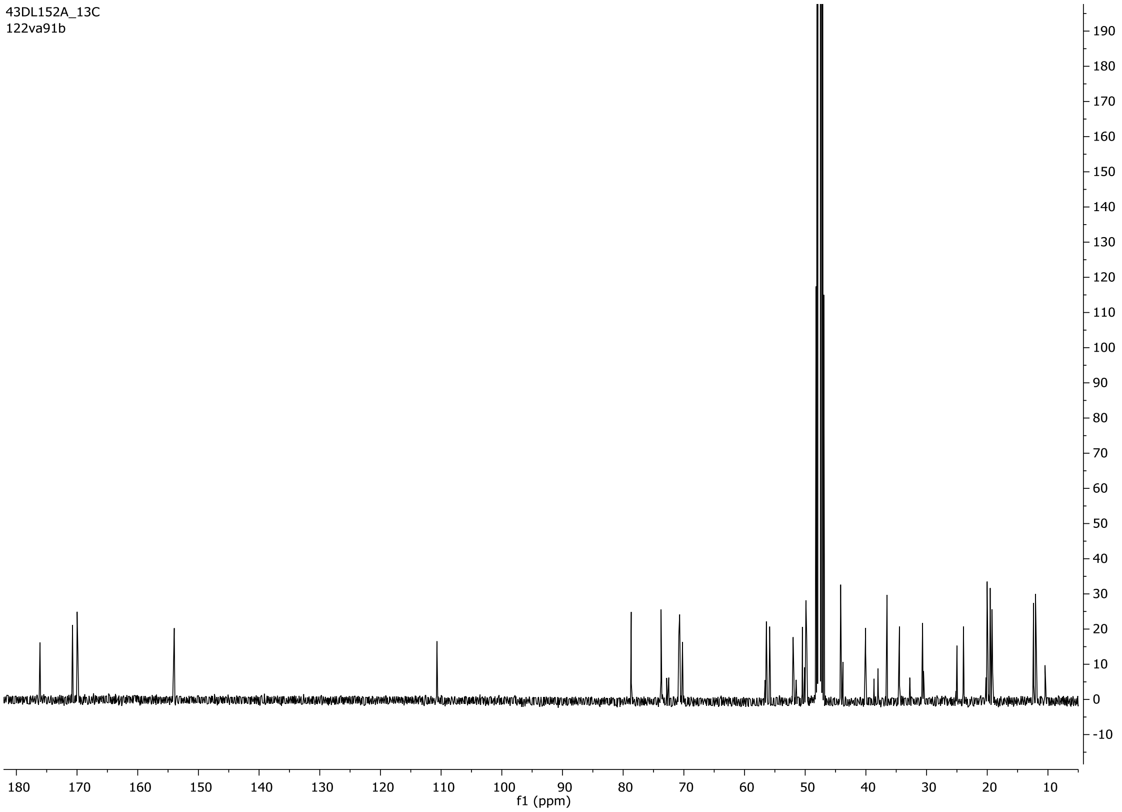

Supplementary Figure 14. ^{13}C NMR spectrum of **TFA-14** in methanol-*d*$_4$

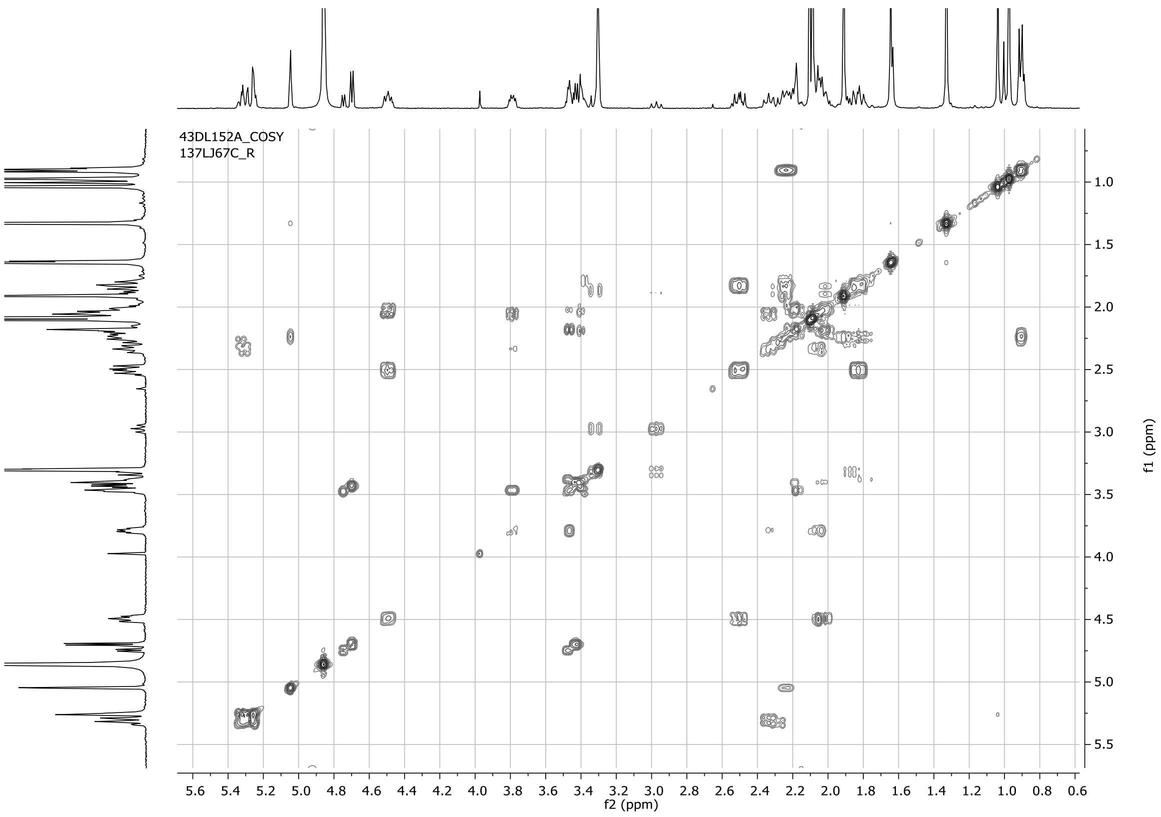

Supplementary Figure 15. ^{1}H-^{1}H COSY spectrum of **TFA-14** in methanol-d_4

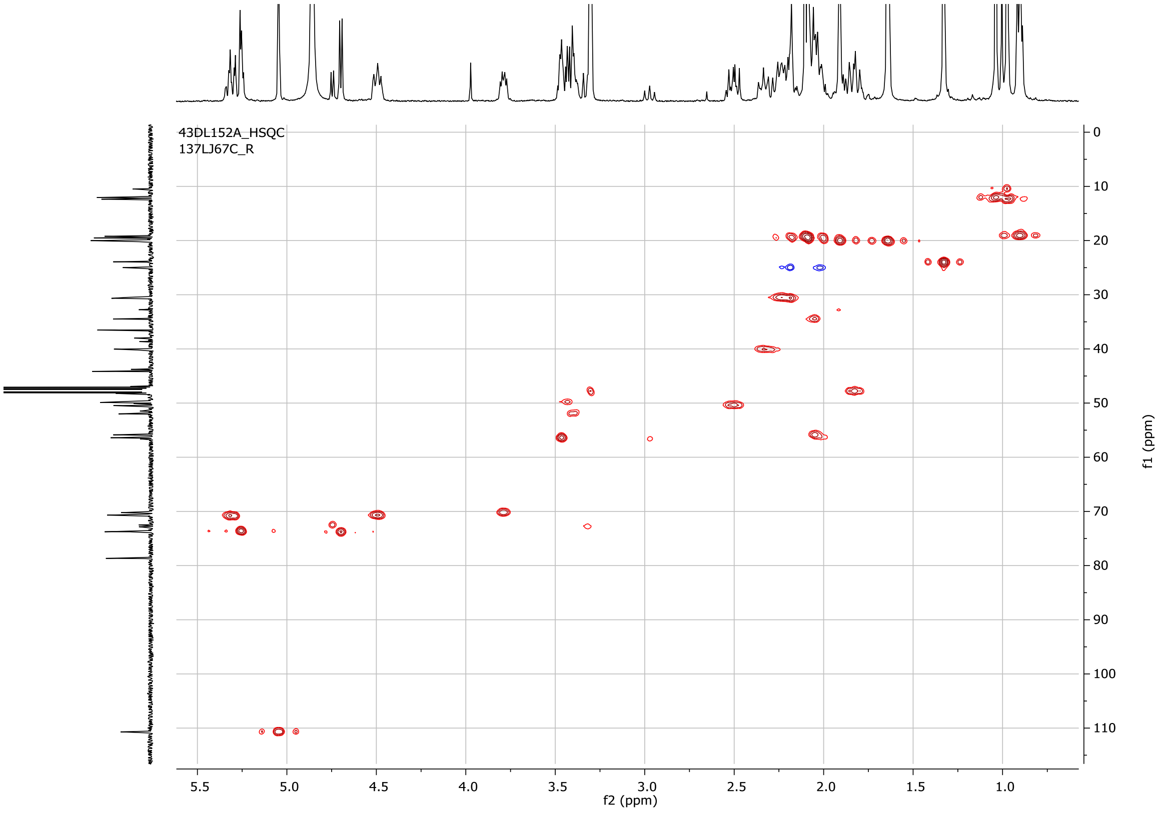

Supplementary Figure 16. HSQC spectrum of **TFA-14** in methanol-d_4

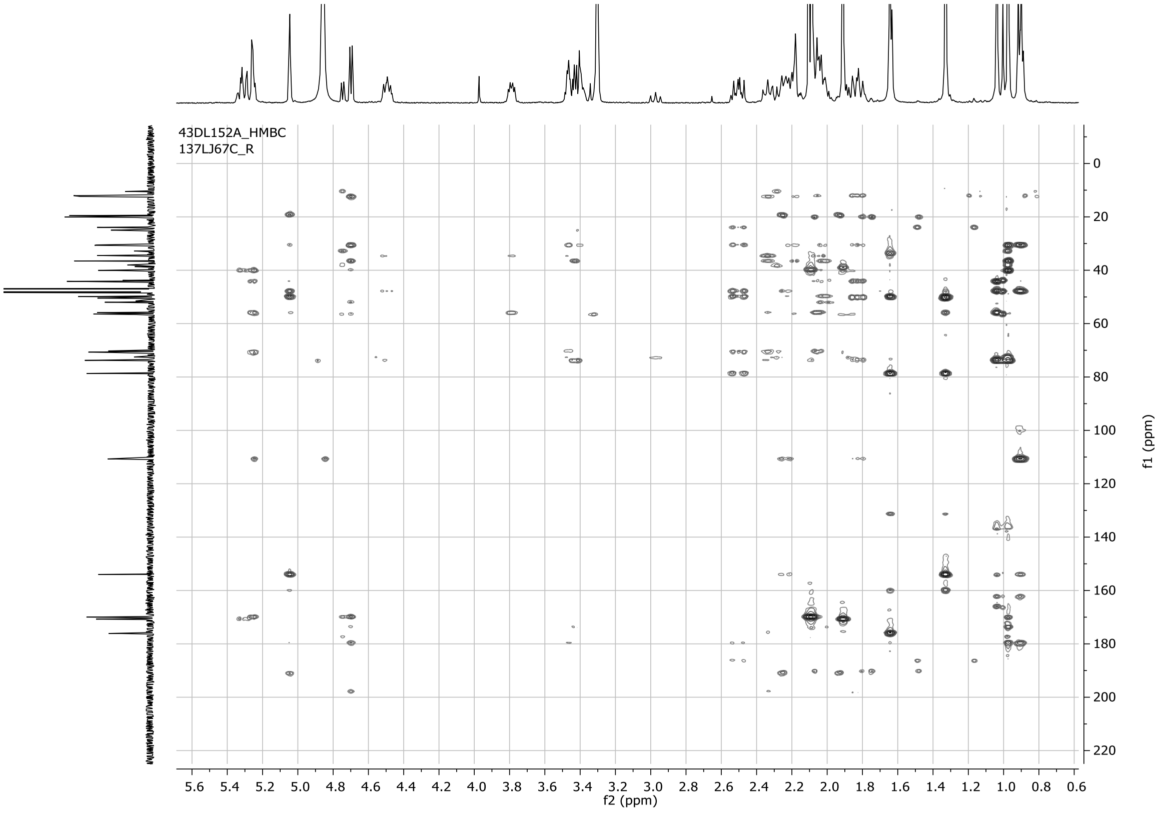

Supplementary Figure 17. HMBC spectrum of **TFA-14** in methanol-d_4

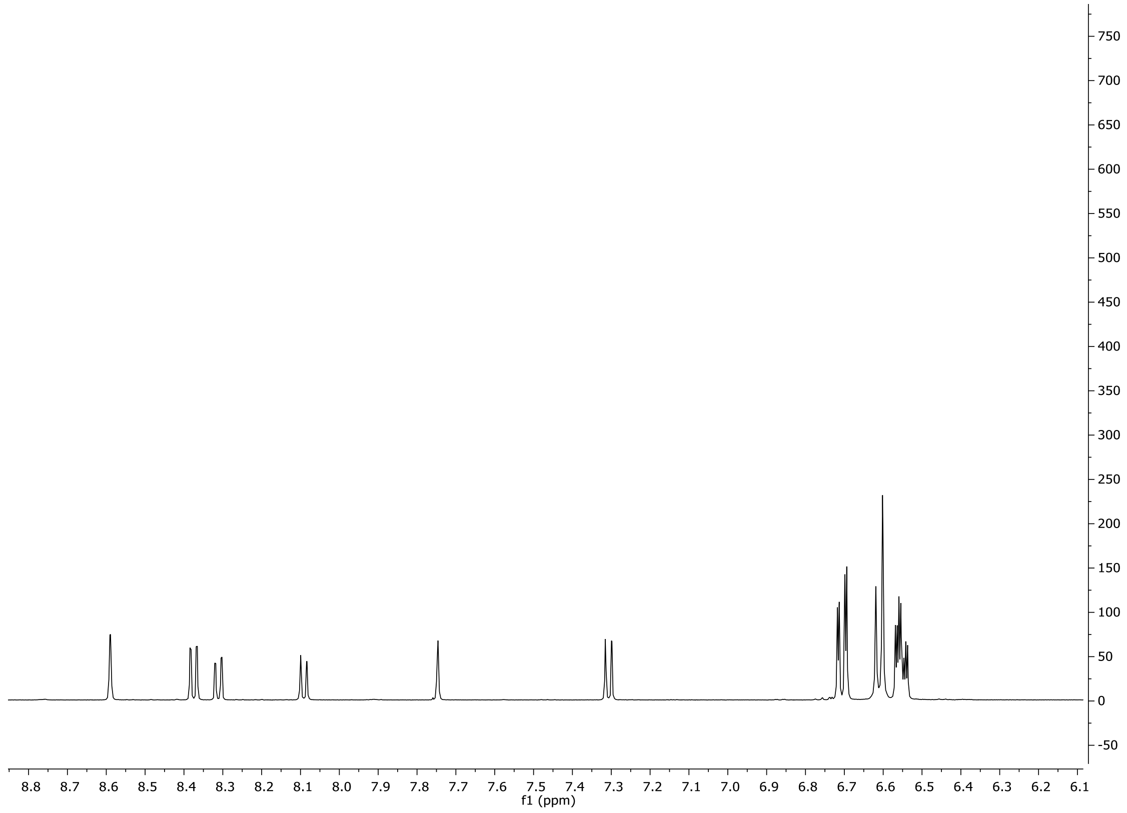

Supplementary Figure 18. ^{1}H NMR spectrum of **15** in methanol-d_4

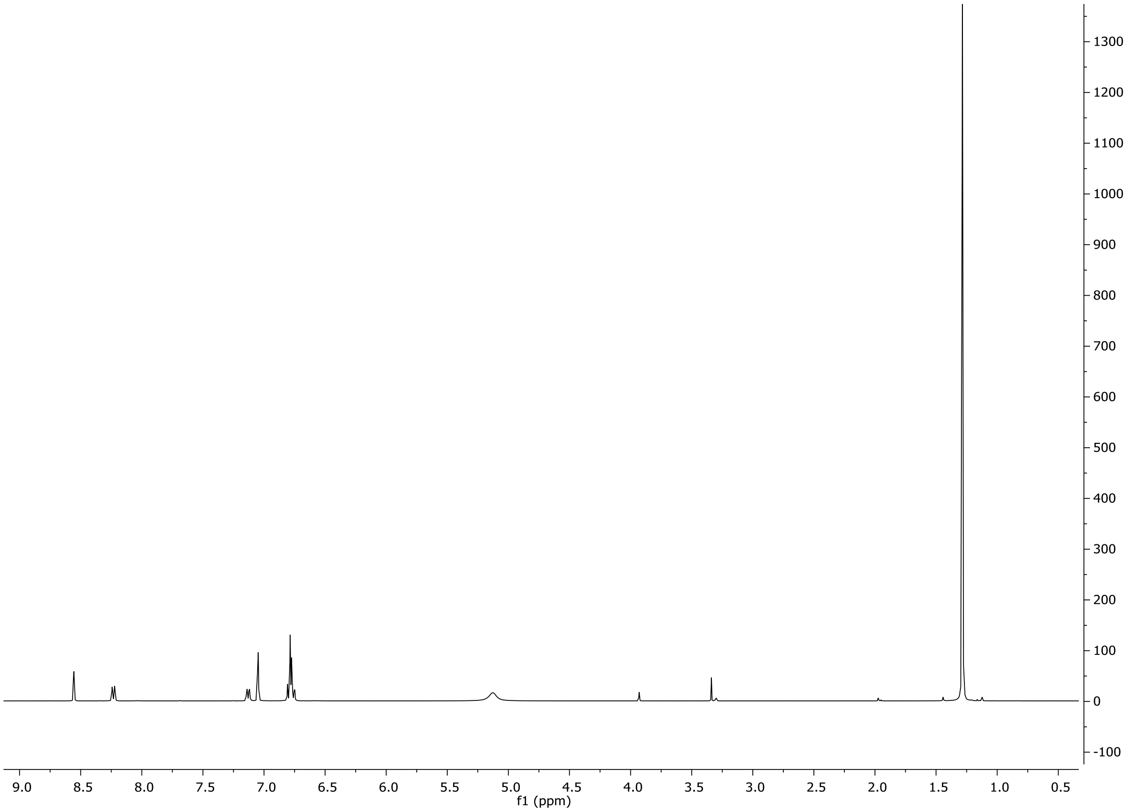

Supplementary Figure 19. ^{1}H NMR spectrum of **16** in methanol-d_4

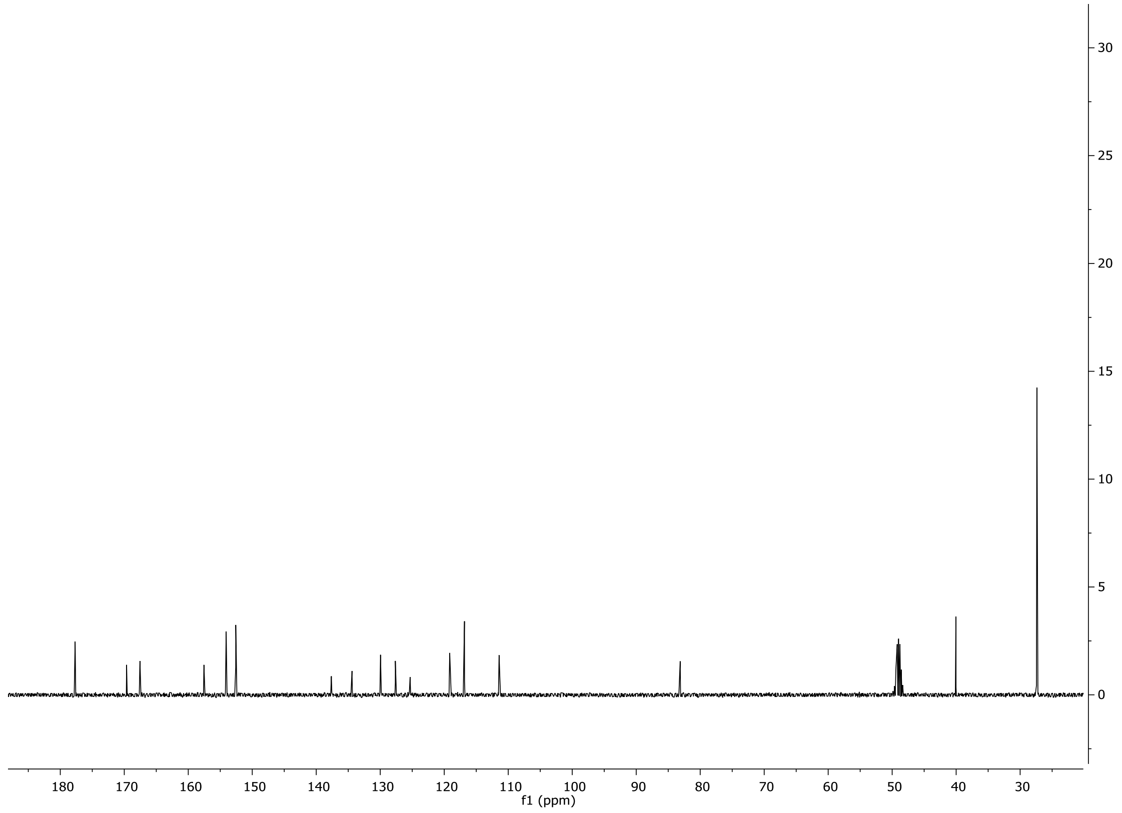

Supplementary Figure 20. ^{13}C NMR spectrum of **16** in methanol-d_4

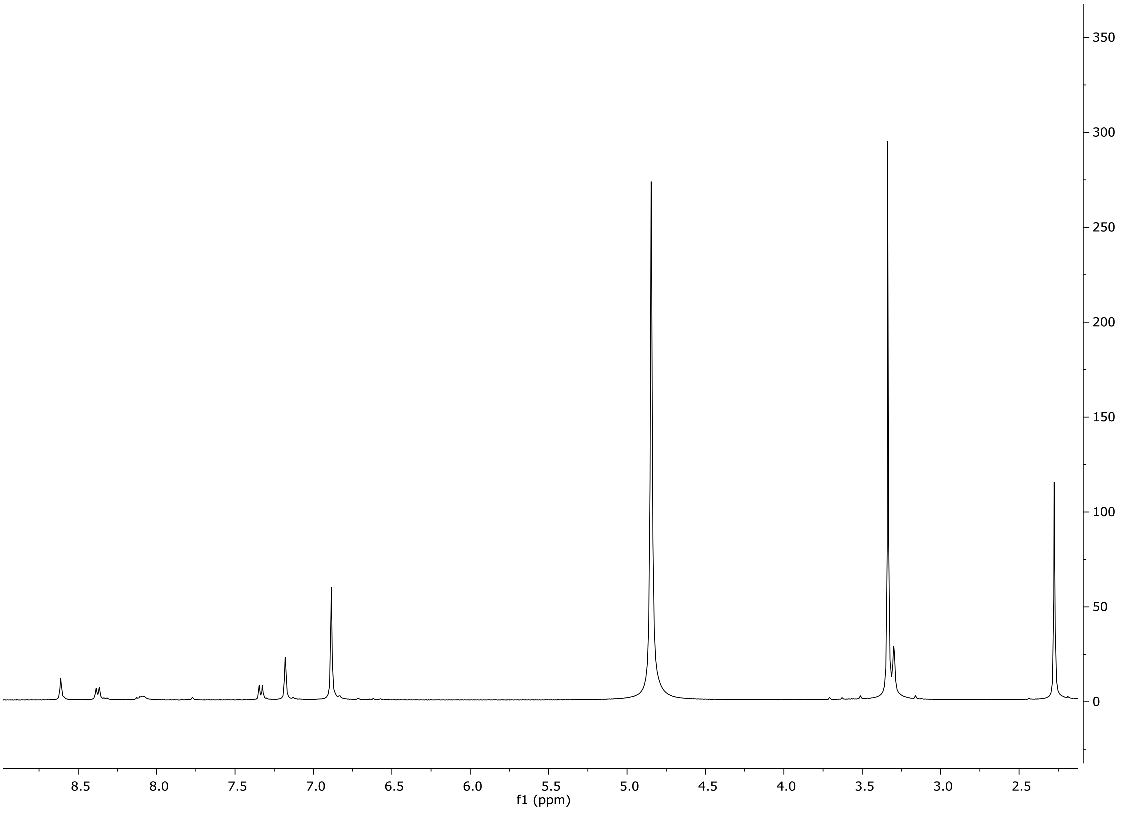

Supplementary Figure 21. ^{1}H NMR spectrum of **17** in methanol-d_4

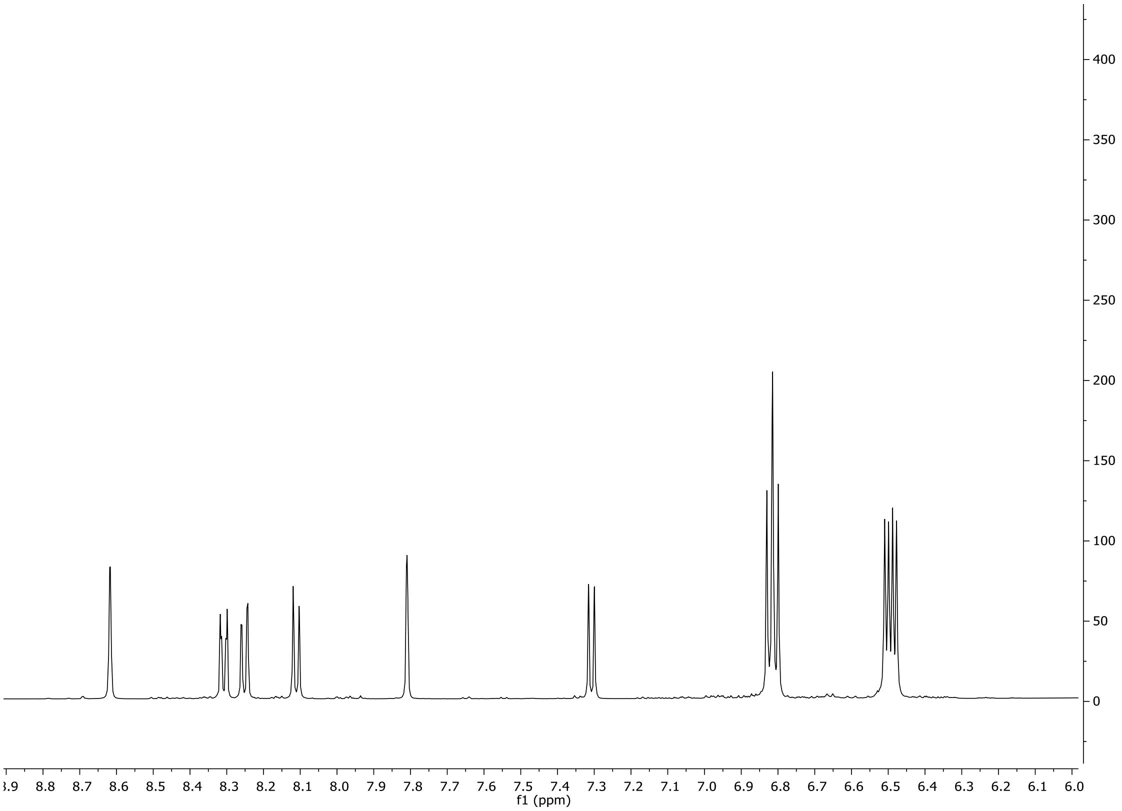

Supplementary Figure 22. ^{1}H NMR spectrum of **19** in methanol-d_4

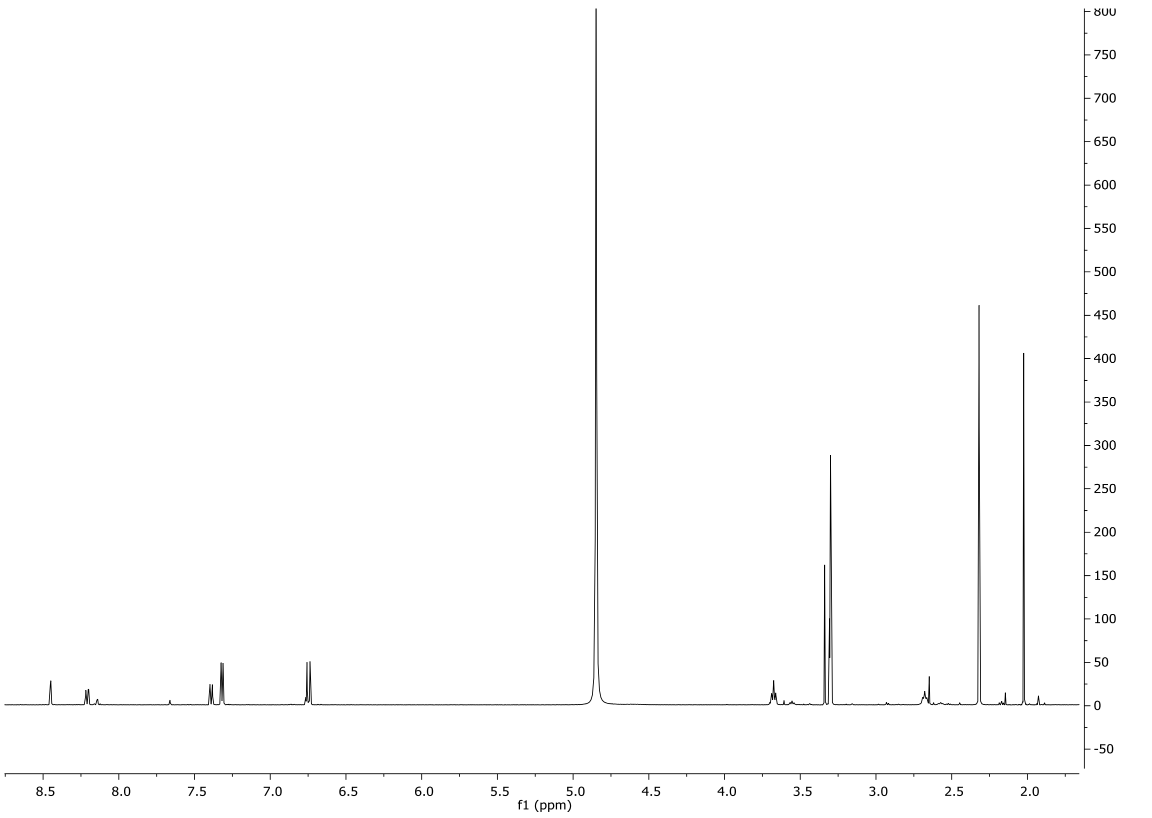

Supplementary Figure 23. ^{1}H NMR spectrum of **20** in methanol-d_4

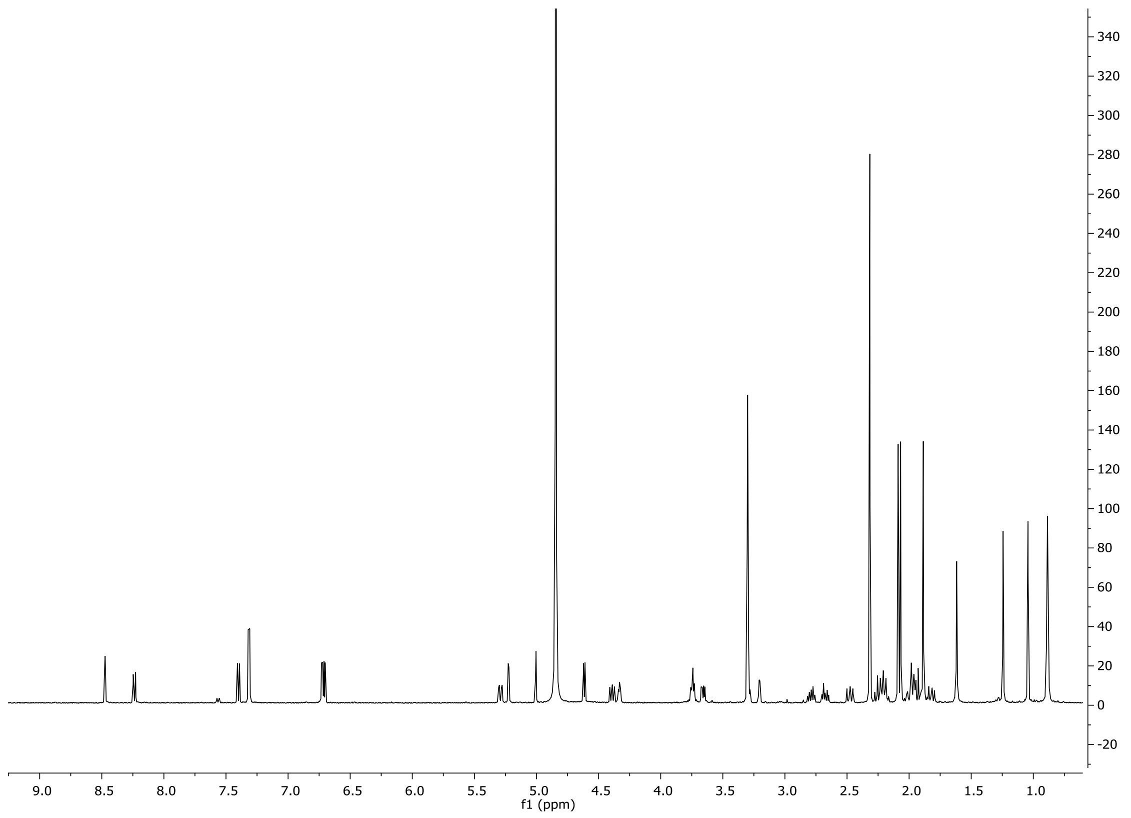

Supplementary Figure 24. ^{1}H NMR spectrum of **21** in methanol-d_4

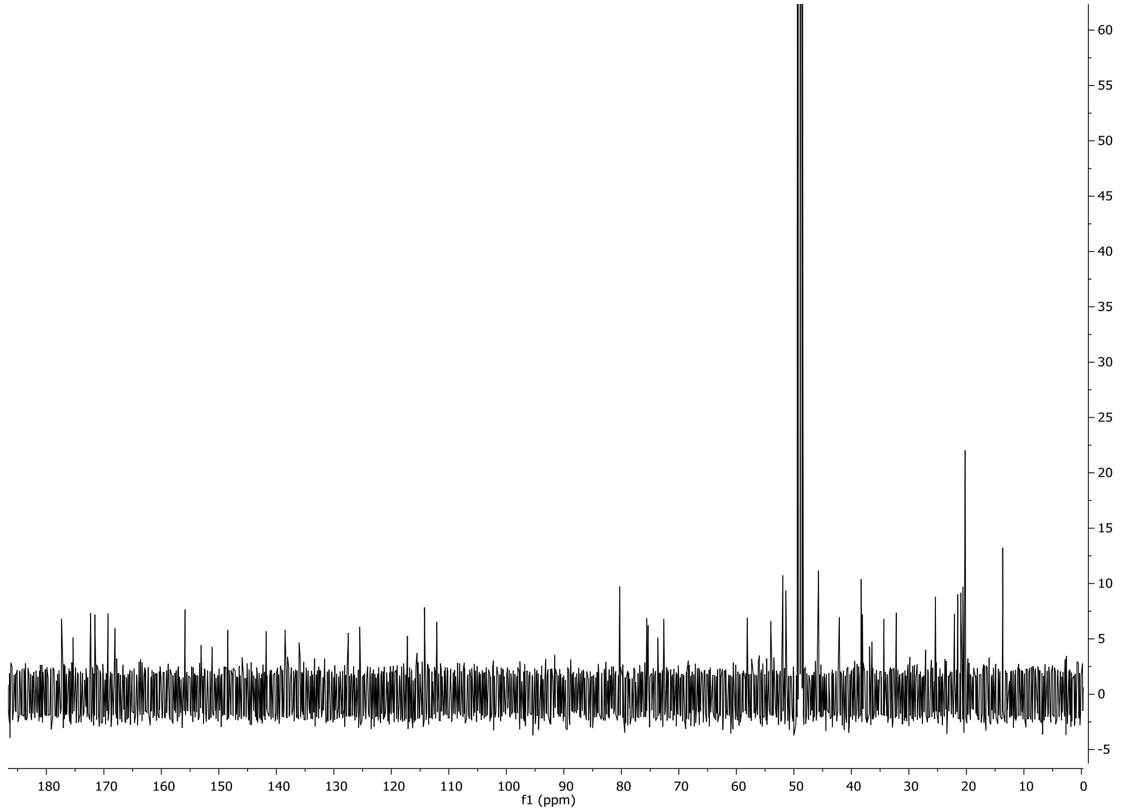

Supplementary Figure 25. ^{13}C NMR spectrum of **21** in methanol-d_4

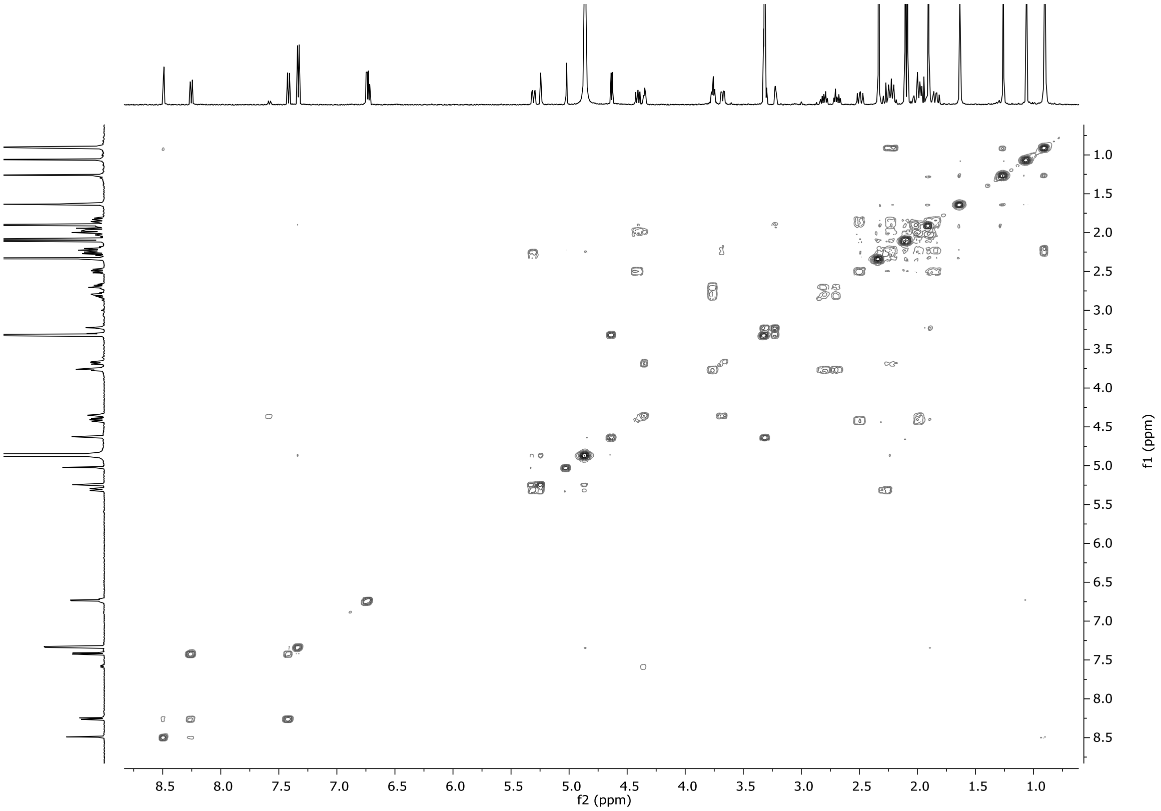

Supplementary Figure 26. ^{1}H-^{1}H COSY NMR spectrum of **21** in methanol-d_4

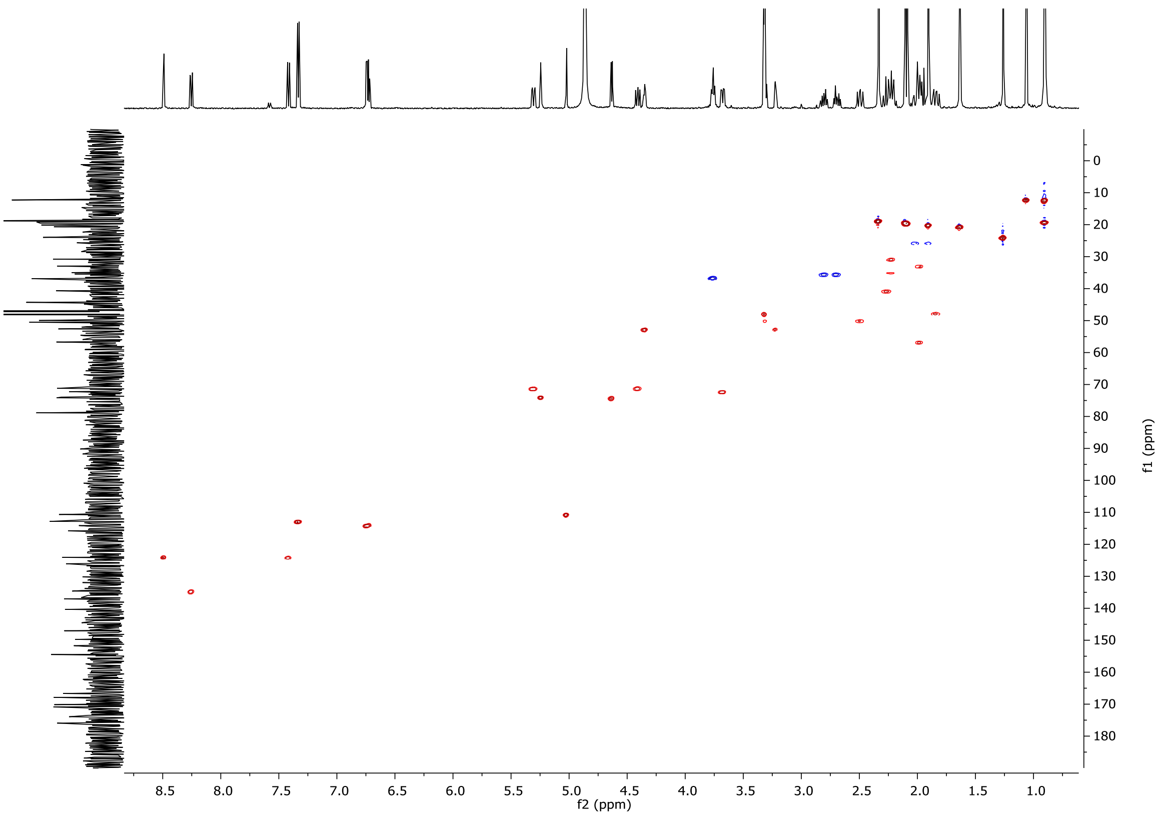

Supplementary Figure 27. HSQC NMR spectrum of **21** in methanol-d_4

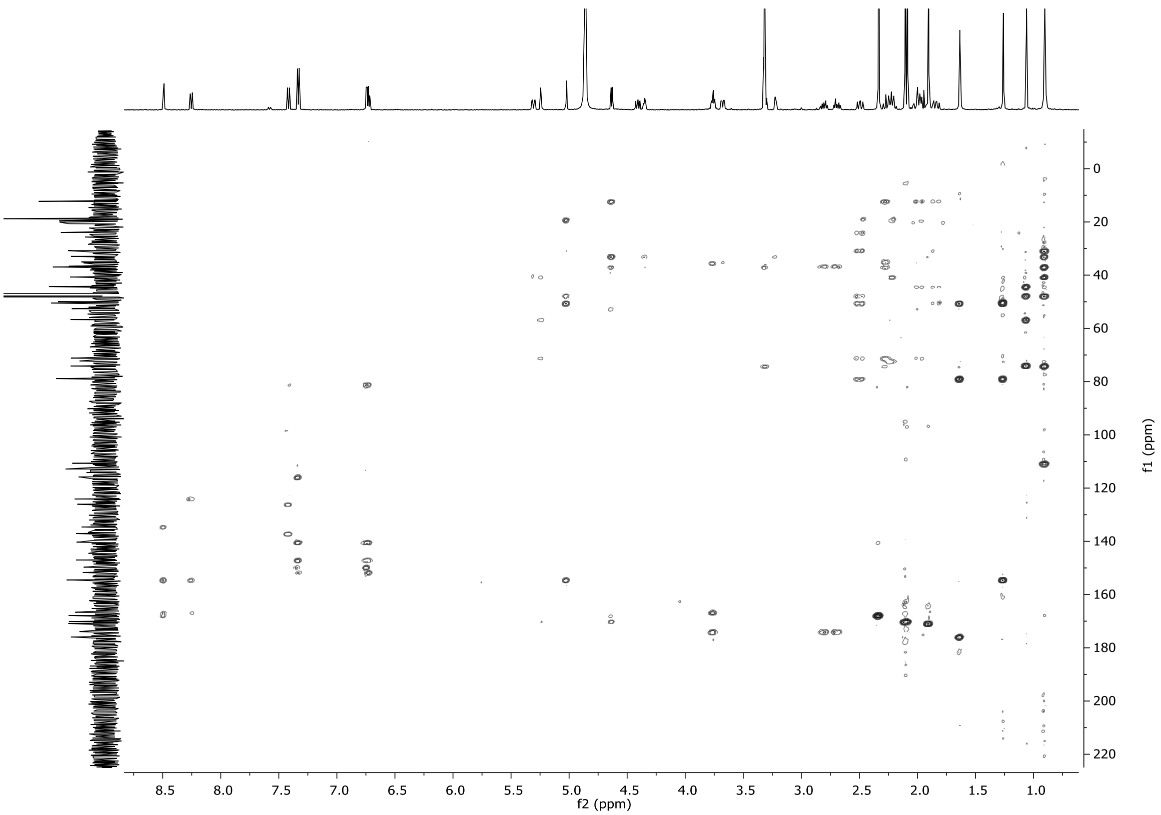

Supplementary Figure 28. HMBC NMR spectrum of **21** in methanol-d_4

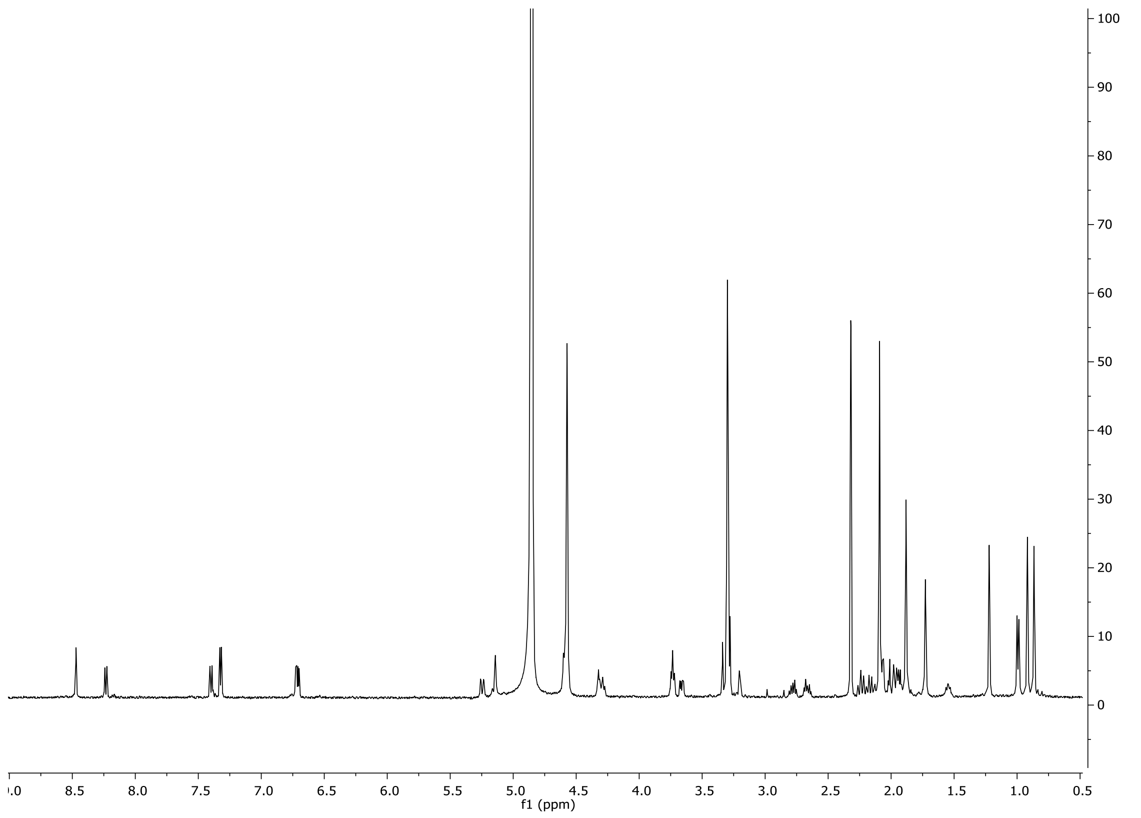

Supplementary Figure 29. ^{1}H NMR spectrum of **4** in methanol-d_4

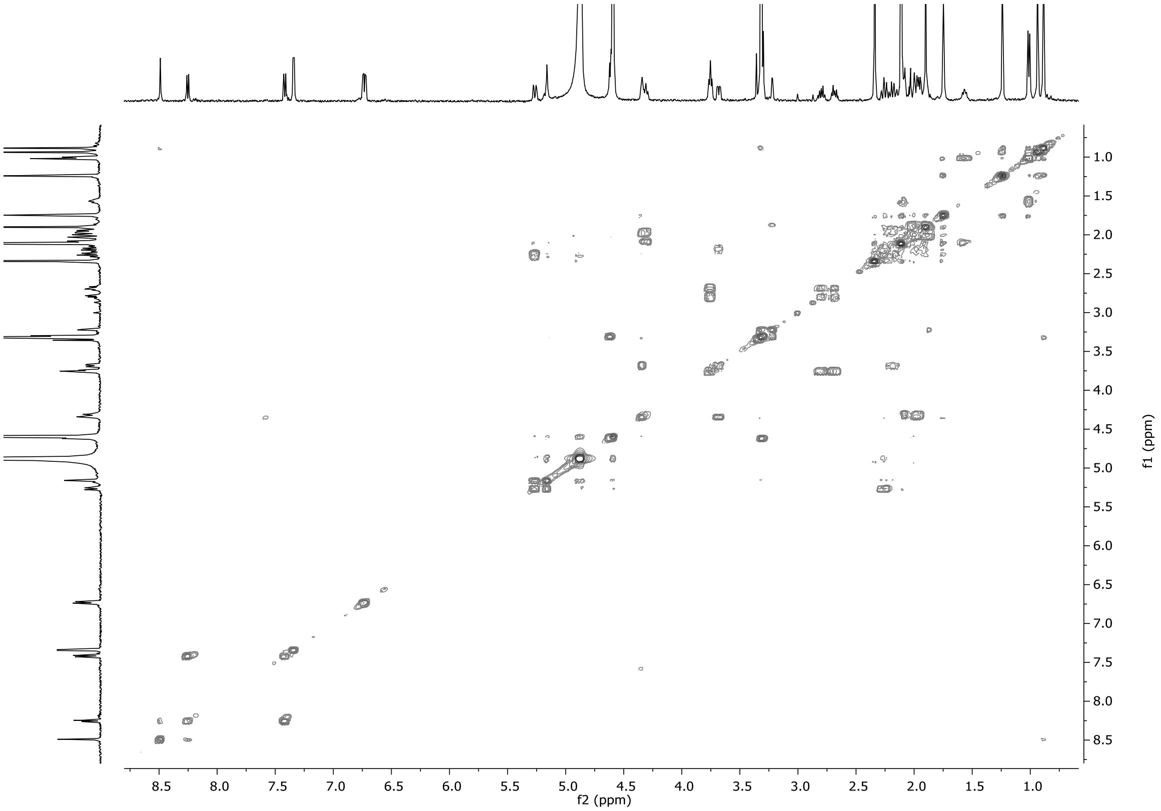

Supplementary Figure 30. ^{1}H-^{1}H COSY NMR spectrum of **4** in methanol-d_4

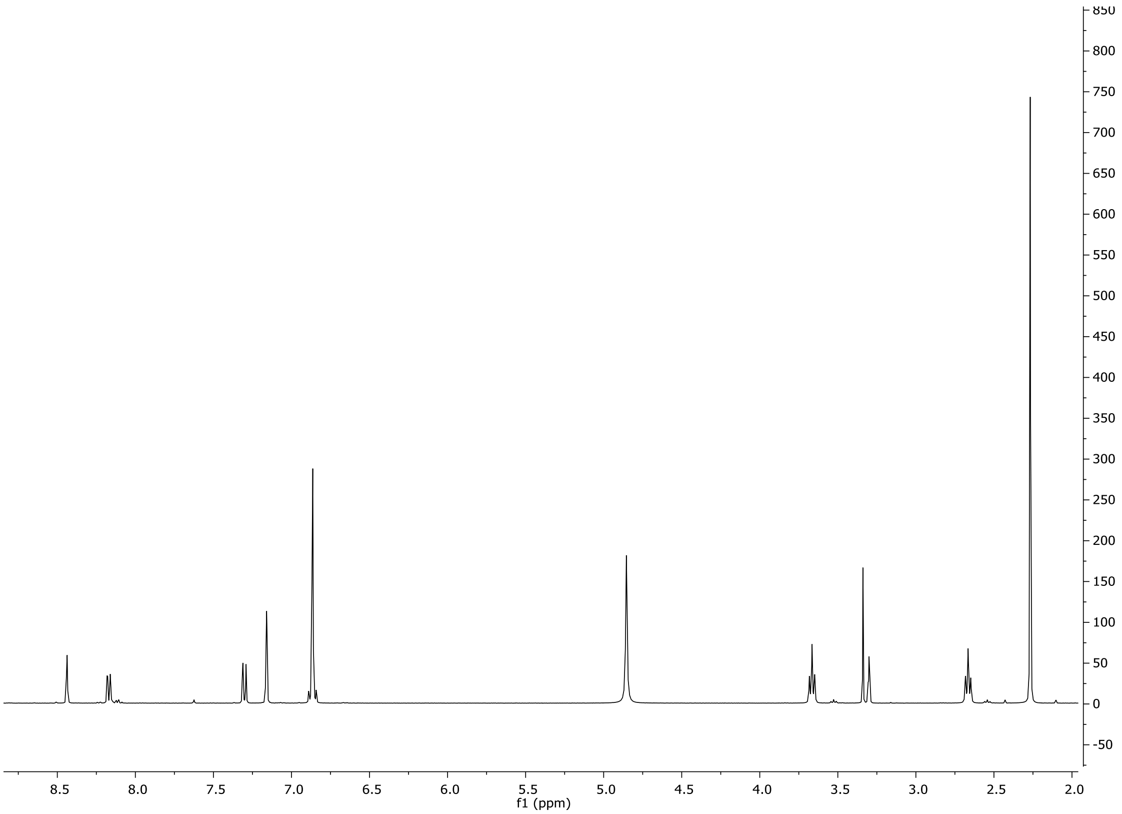

Supplementary Figure 31. ^{1}H NMR spectrum of **22** in methanol-d_4

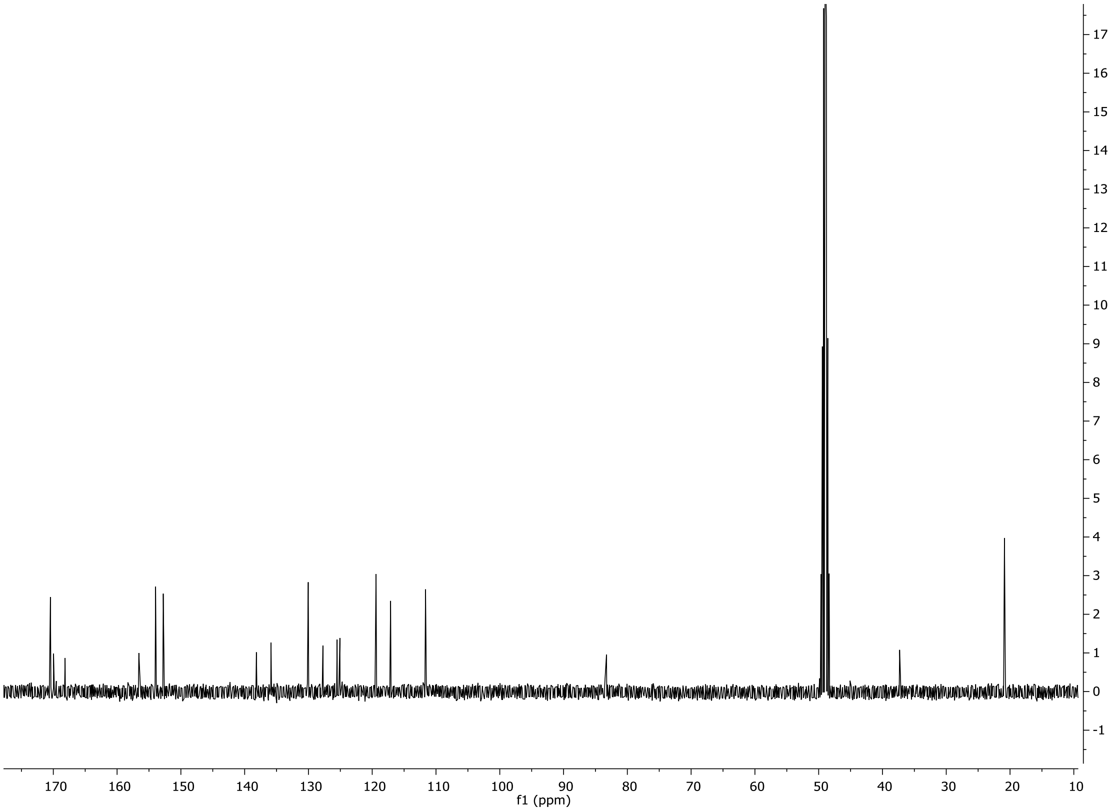

Supplementary Figure 32. ^{13}C NMR spectrum of **22** in methanol-d_4

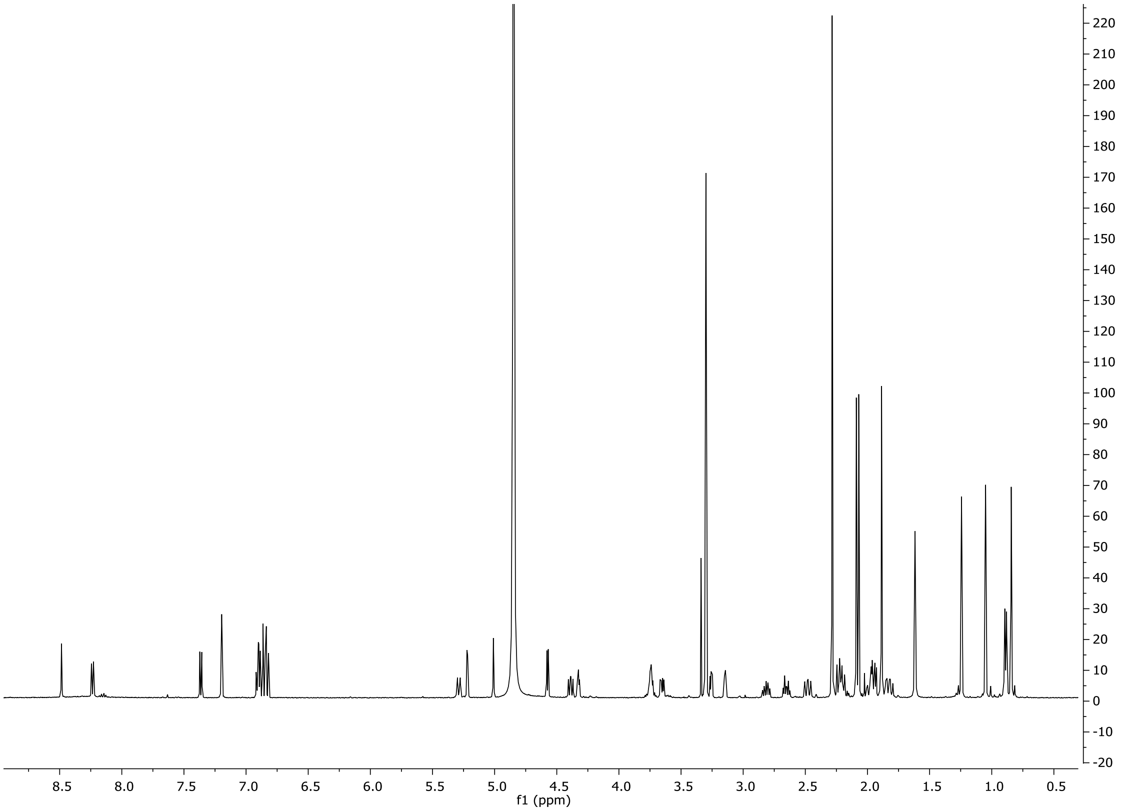

Supplementary Figure 33. ^{1}H NMR spectrum of **23** in methanol-d_4

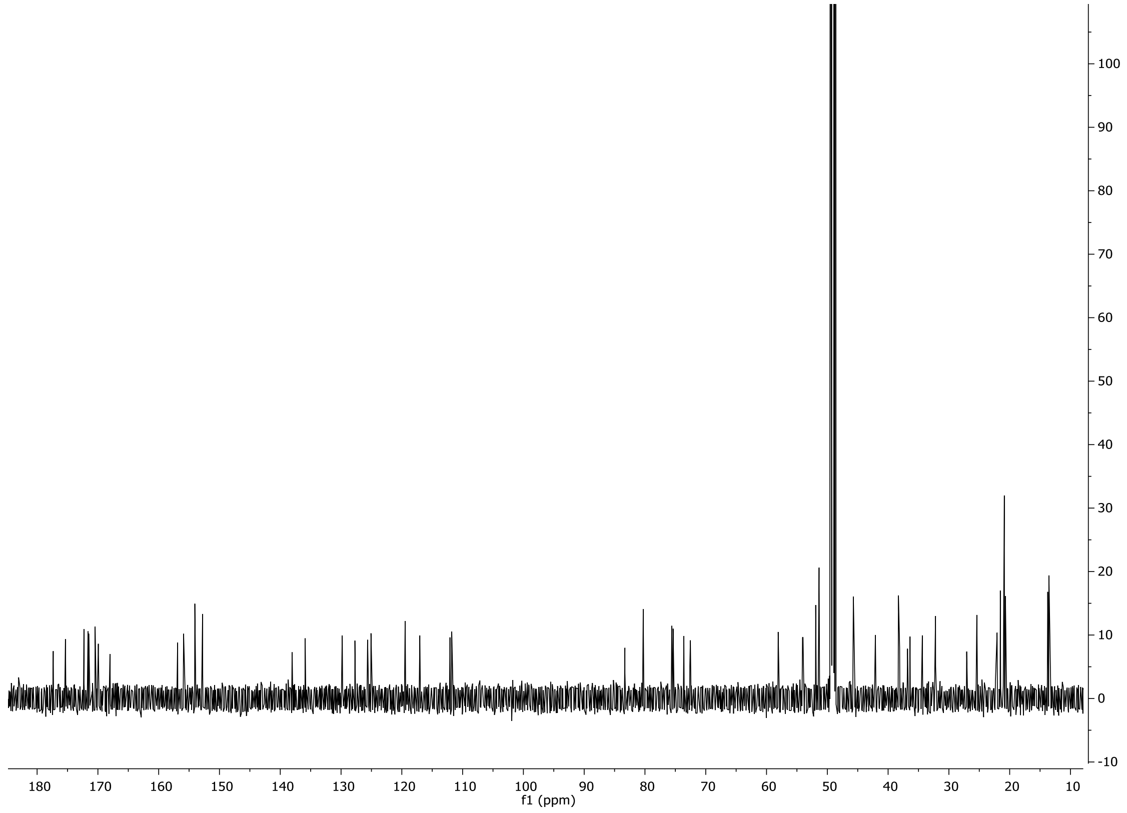

Supplementary Figure 34. ^{13}C NMR spectrum of **23** in methanol-d_4

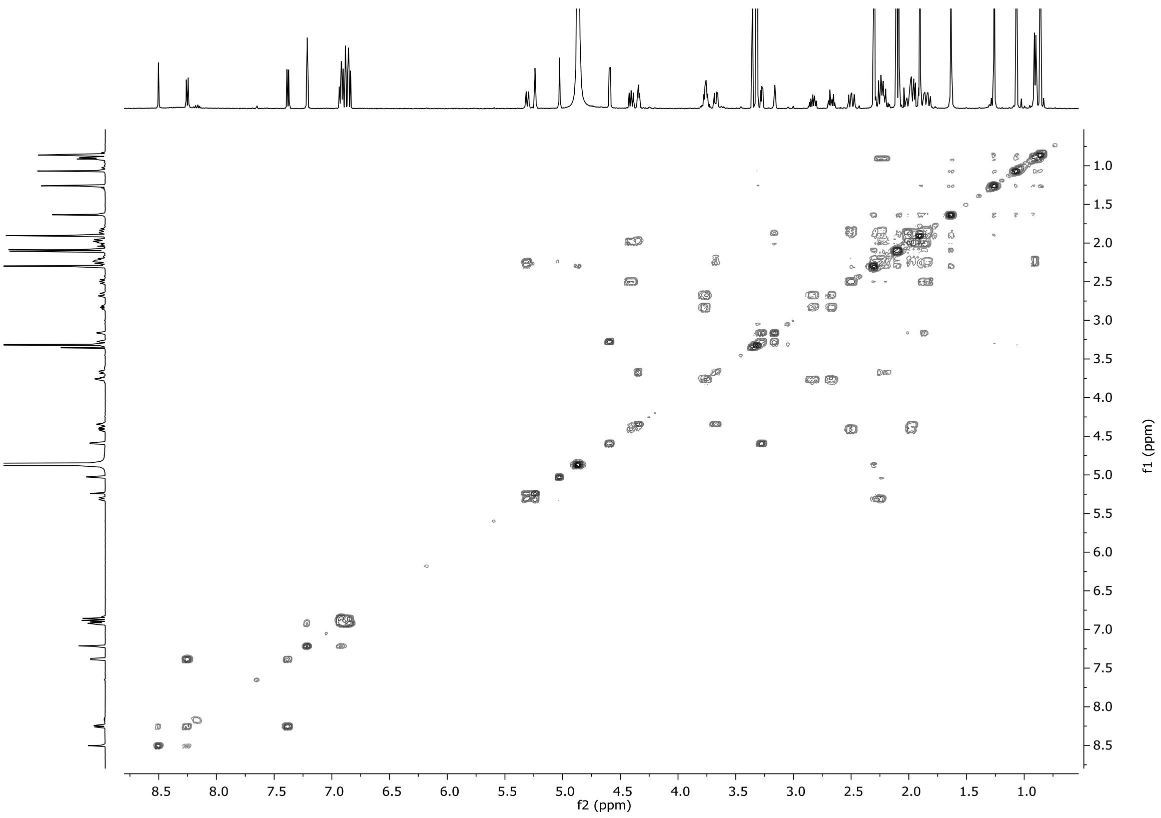

Supplementary Figure 35. ^1H-^1H COSY spectrum of **23** in methanol-d_4

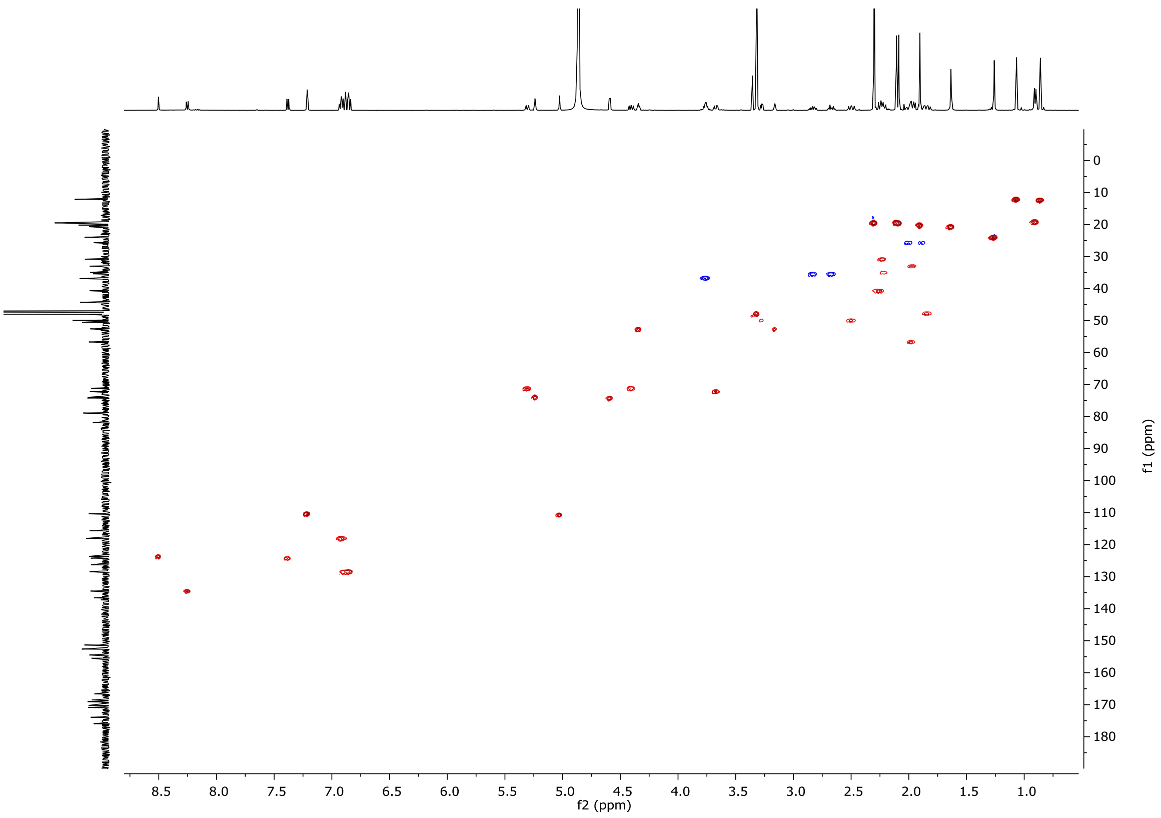

Supplementary Figure 36. HSQC spectrum of **23** in methanol-d_4

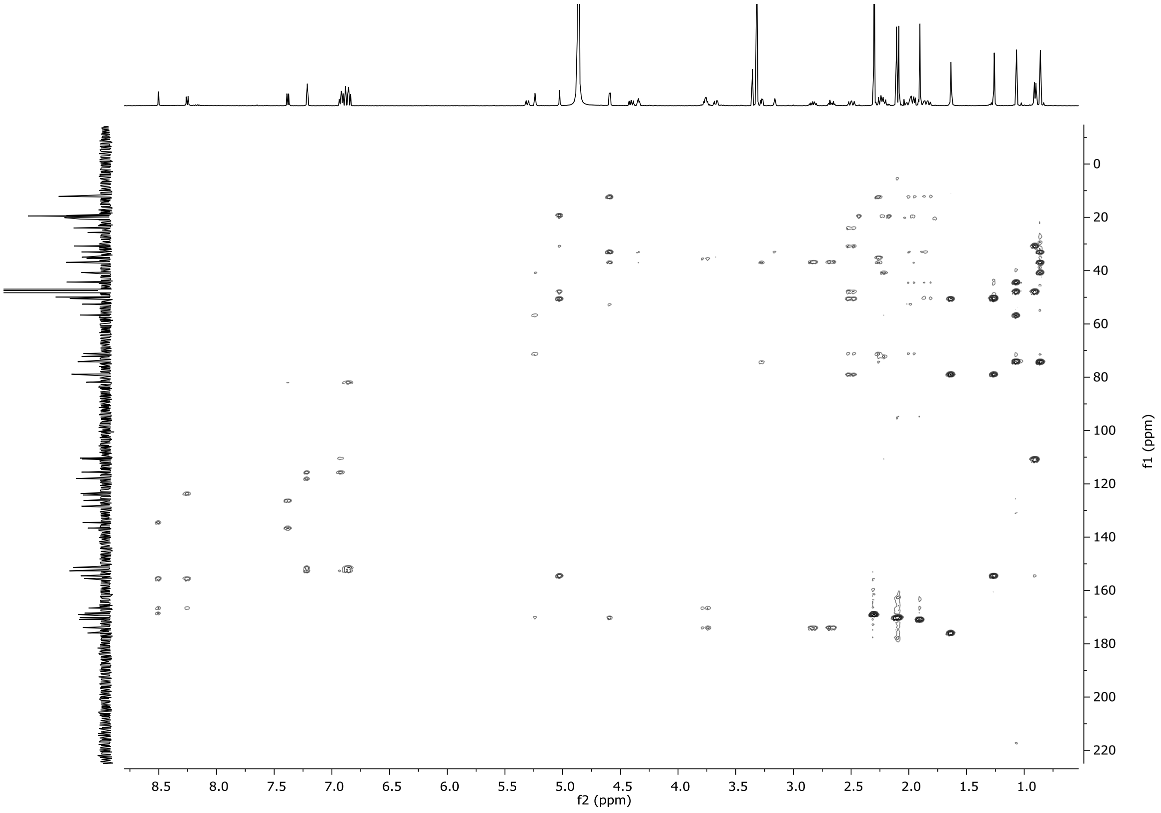

Supplementary Figure 37. HMBC spectrum of **23** in methanol-d_4

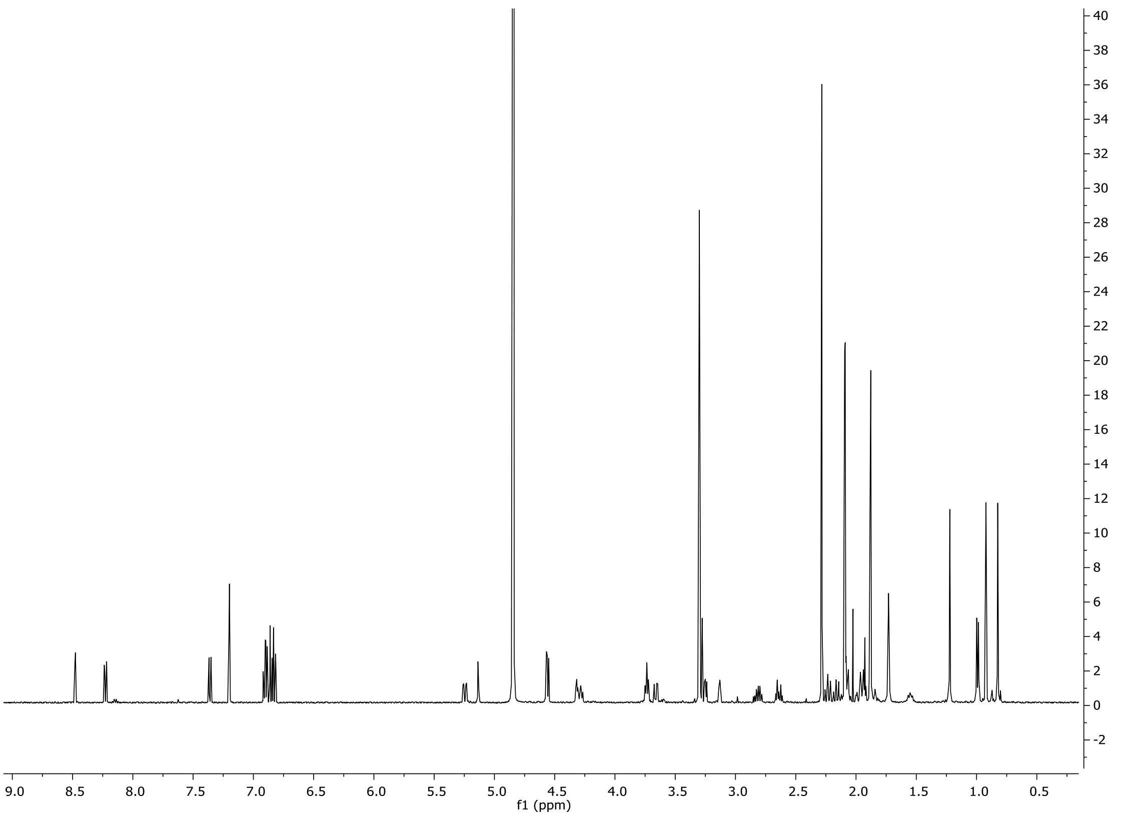

Supplementary Figure 38. ^{1}H NMR spectrum of **5** in methanol-d_4

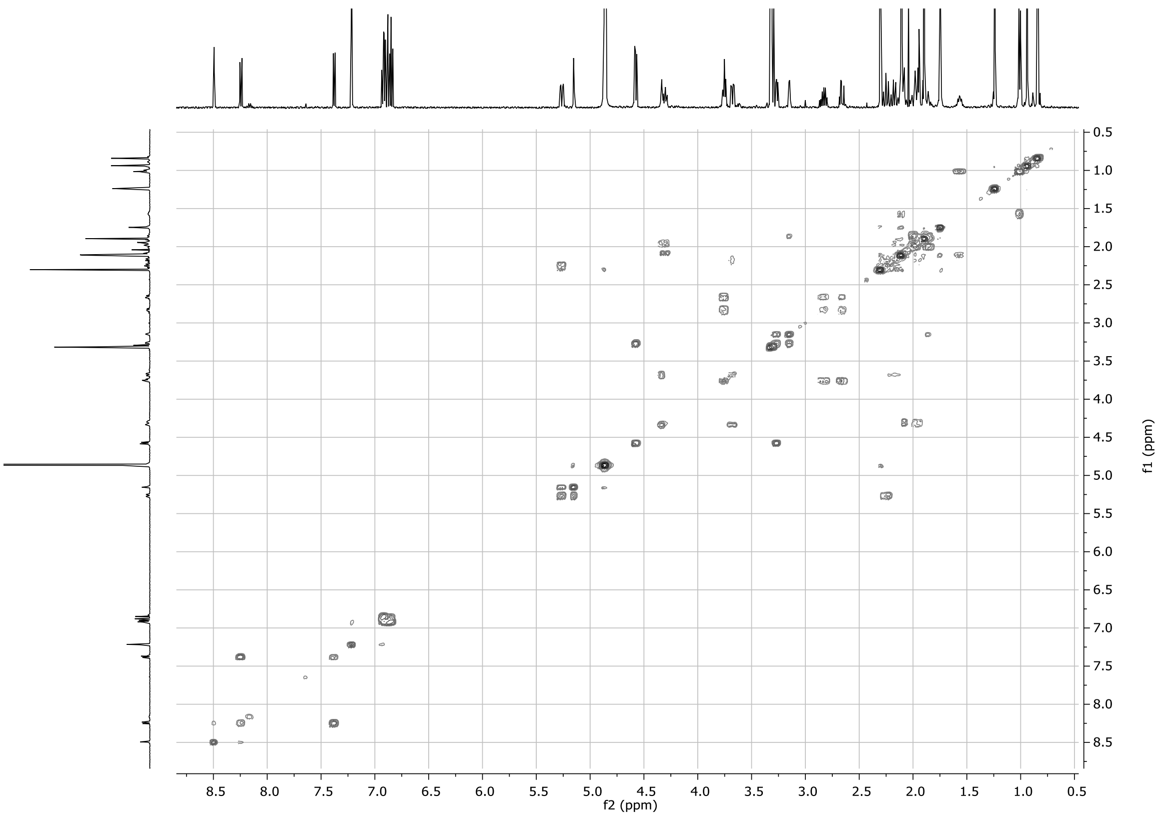

Supplementary Figure 39. ^{1}H-^{1}H COSY spectrum of **5** in methanol-*d*$_4$

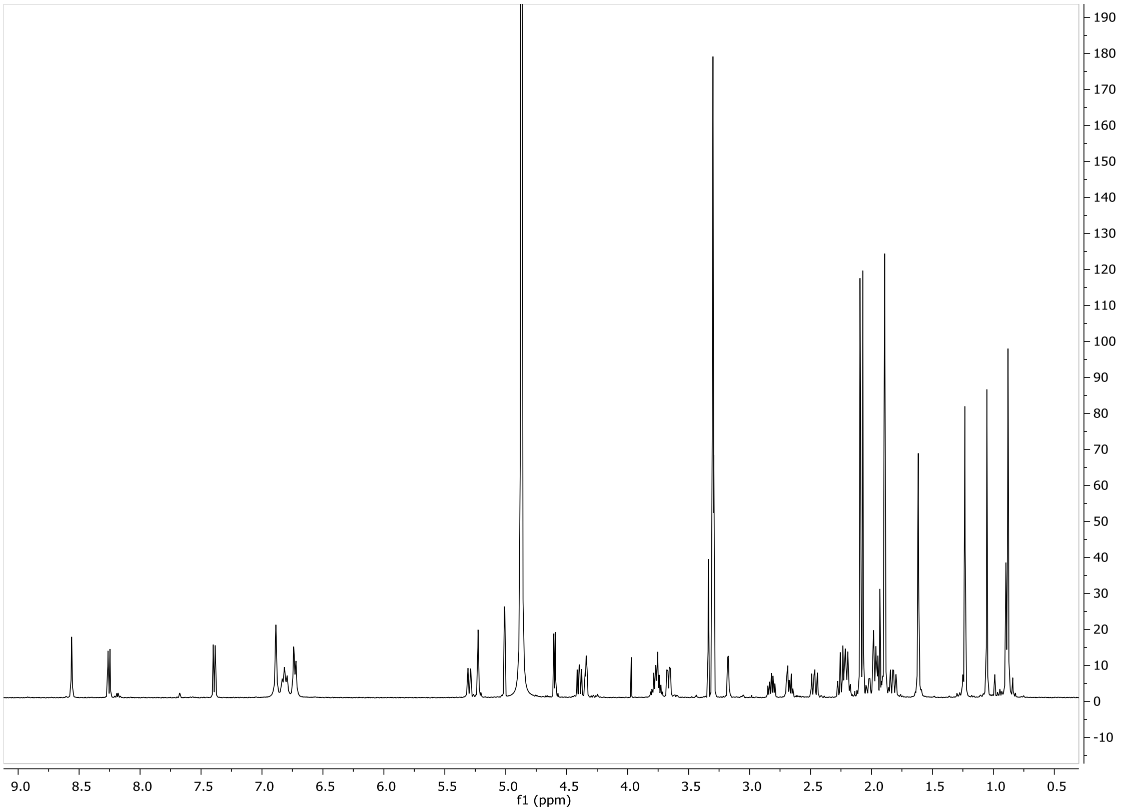

Supplementary Figure 40. ^{1}H NMR spectrum of **24** in methanol-d_4

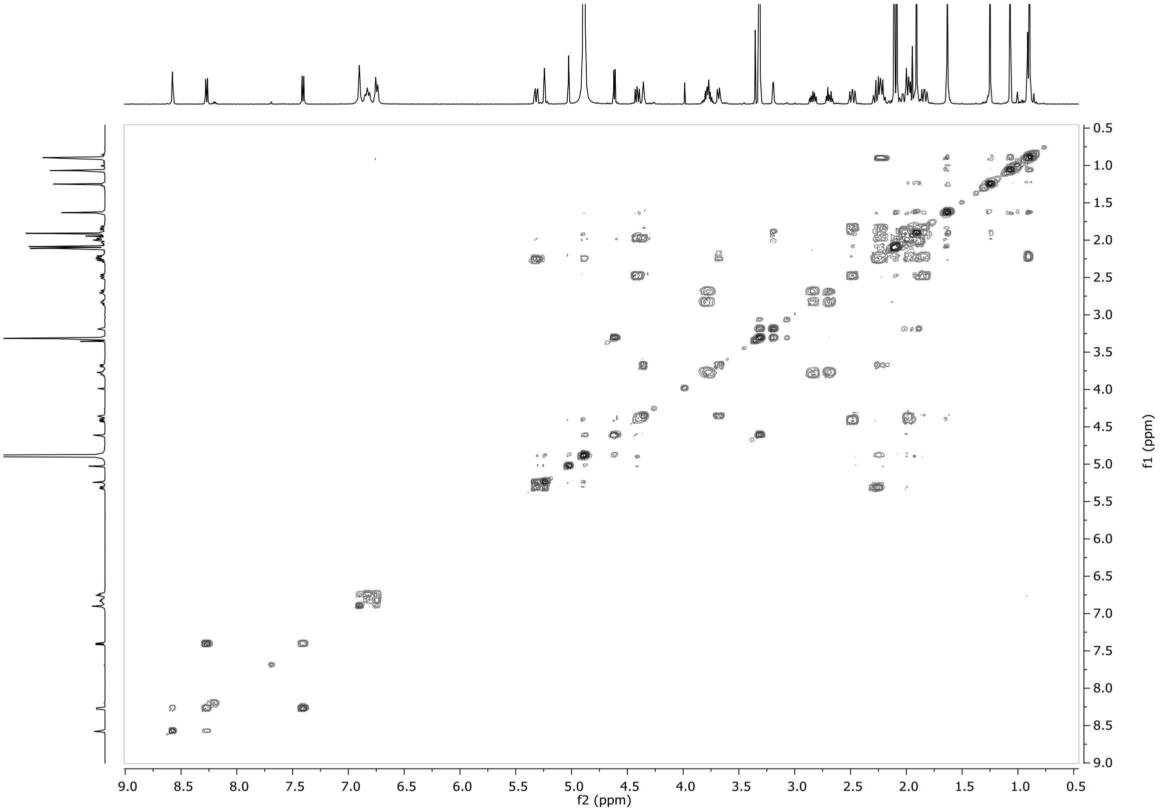

Supplementary Figure 41. ^1H-^1H COSY spectrum of **24** in methanol-d_4

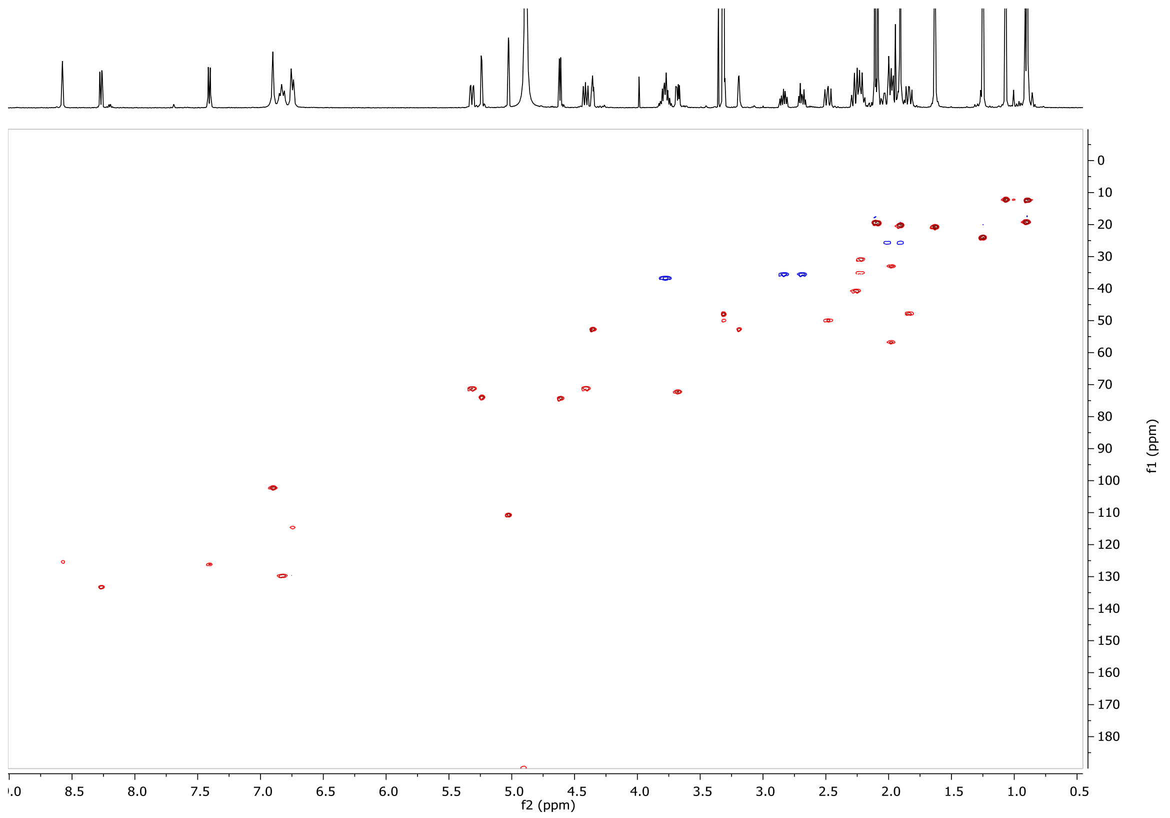

Supplementary Figure 42. HSQC spectrum of **24** in methanol-d_4

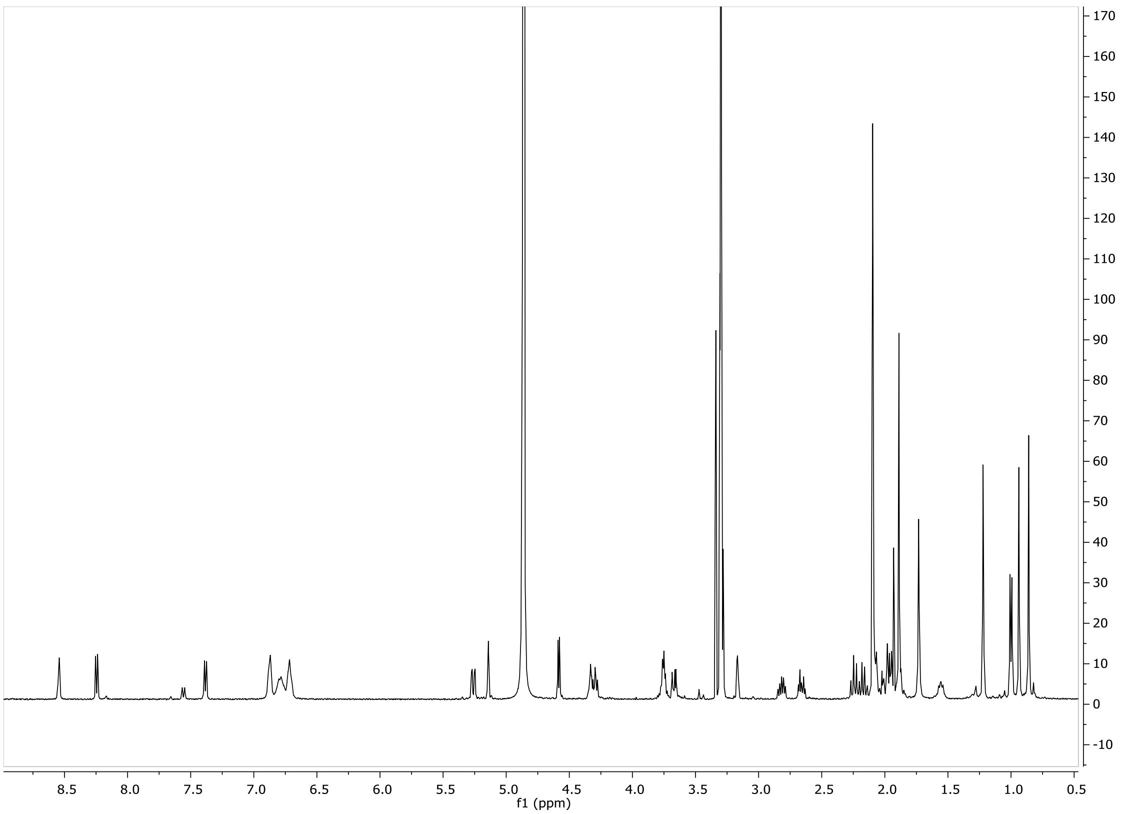

Supplementary Figure 43. ^{1}H NMR spectrum of **6** in methanol-d_4

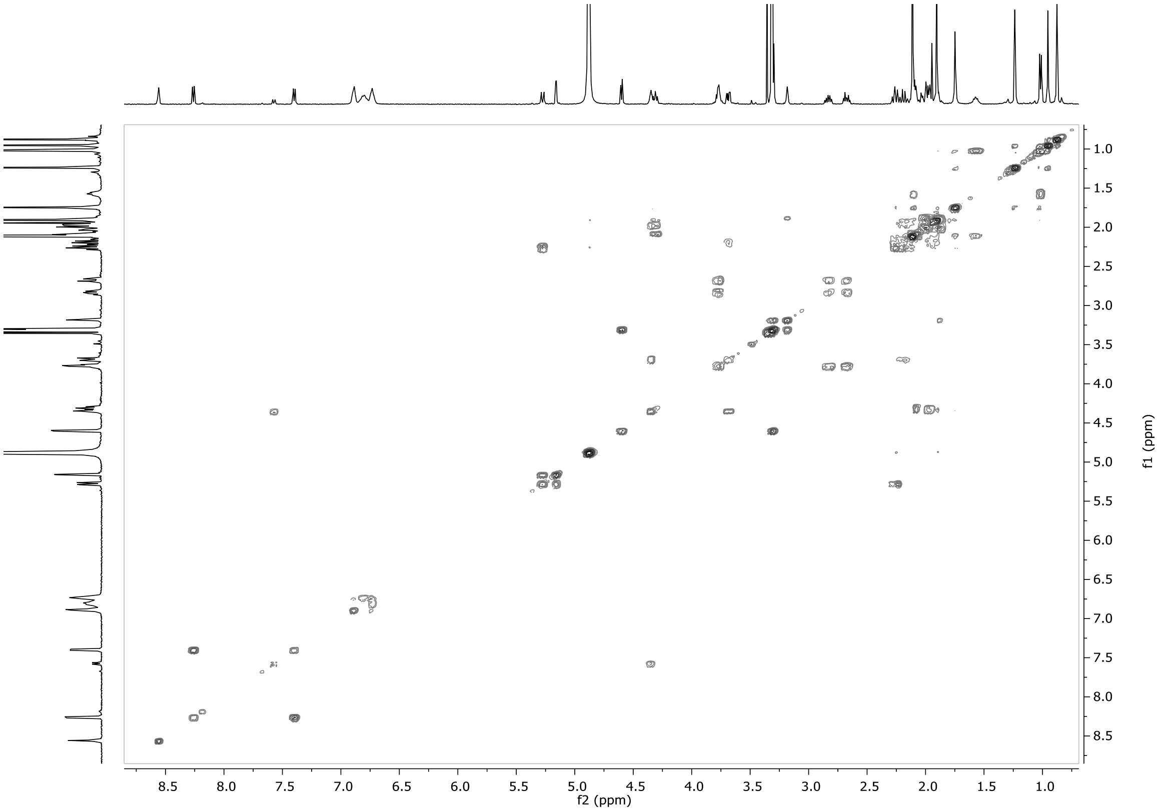

Supplementary Figure 44. ^{1}H-^{1}H COSY spectrum of **6** in methanol-d_4

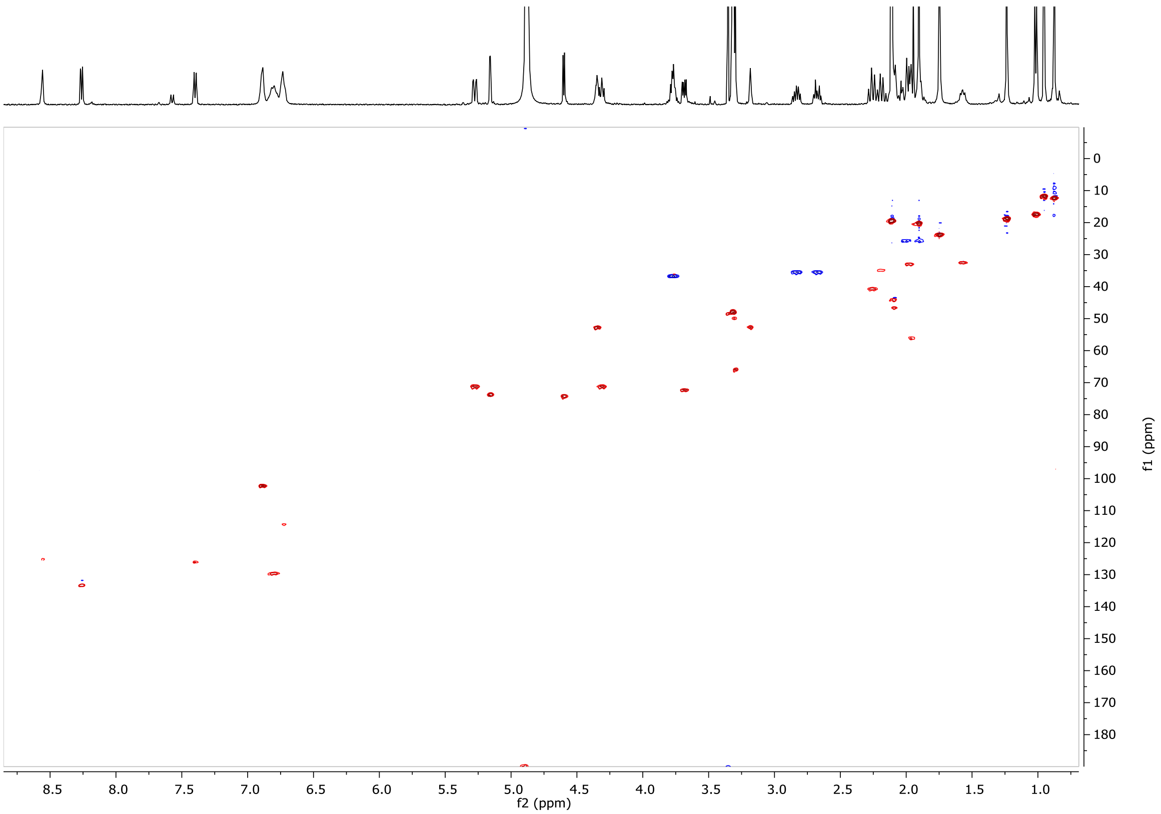

Supplementary Figure 45. HSQC spectrum of **6** in methanol-d_4

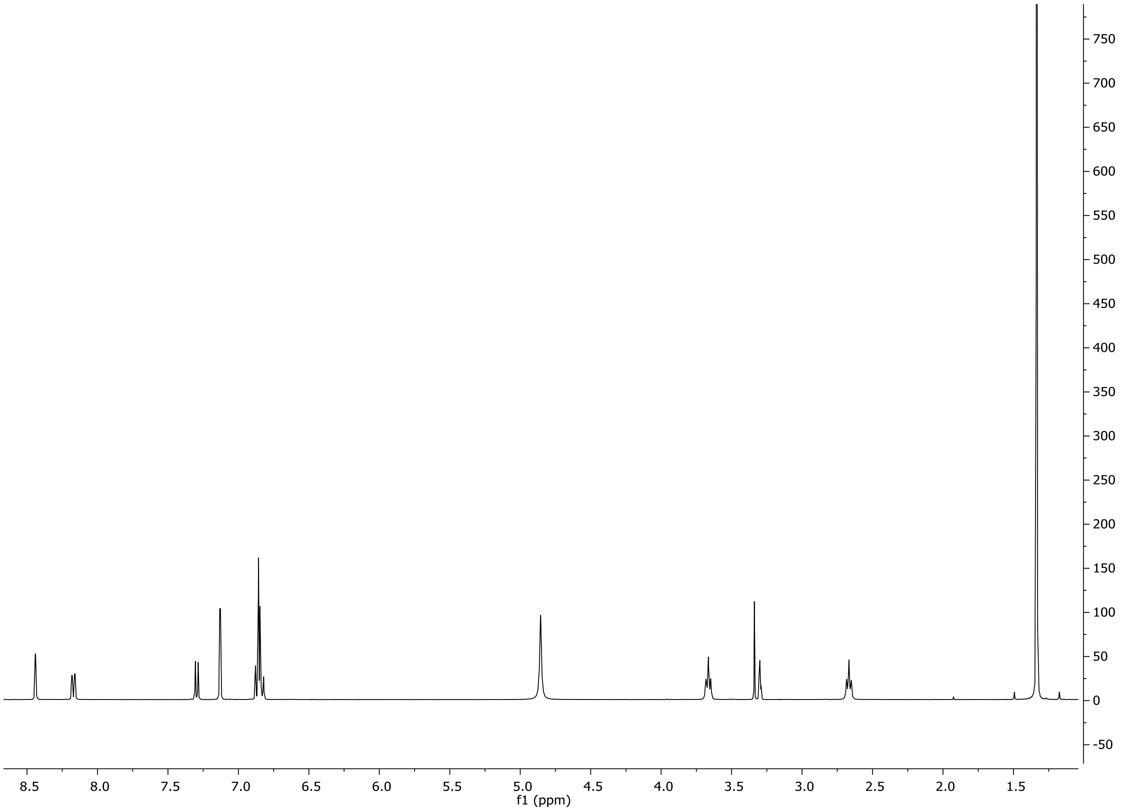

Supplementary Figure 46. ^{1}H NMR spectrum of **25** in methanol-d_4

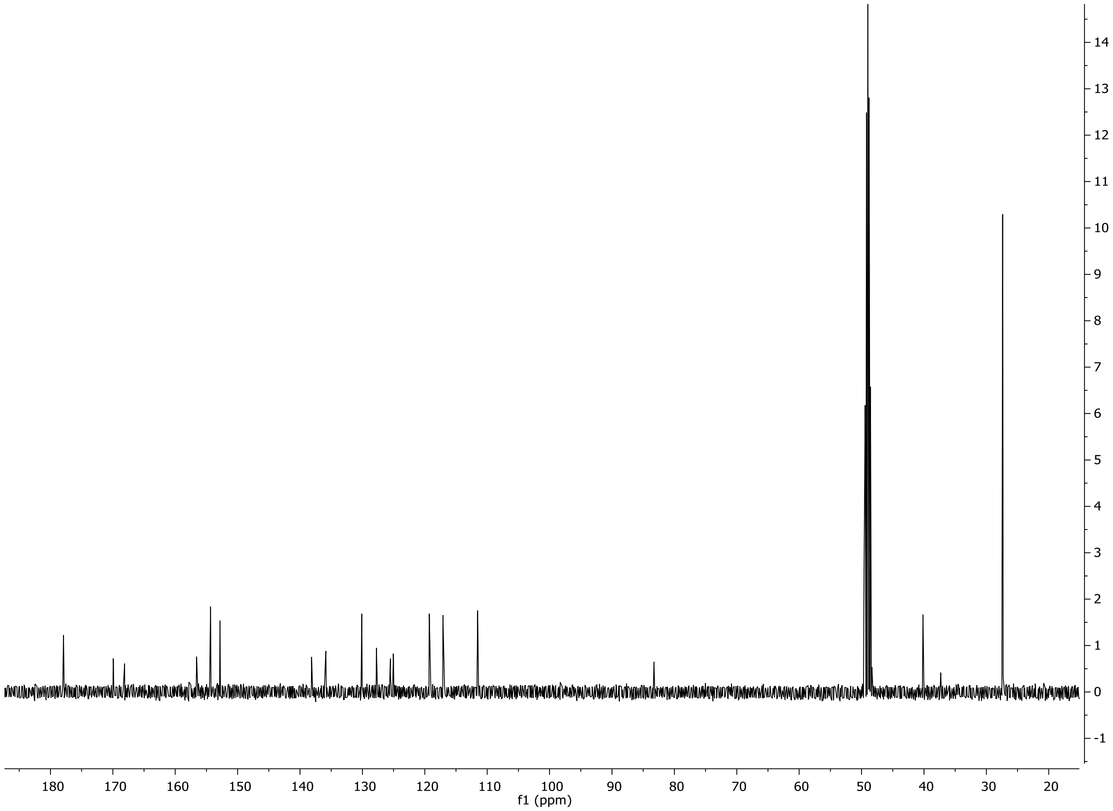

Supplementary Figure 47. ^{13}C NMR spectrum of **25** in methanol-d_4

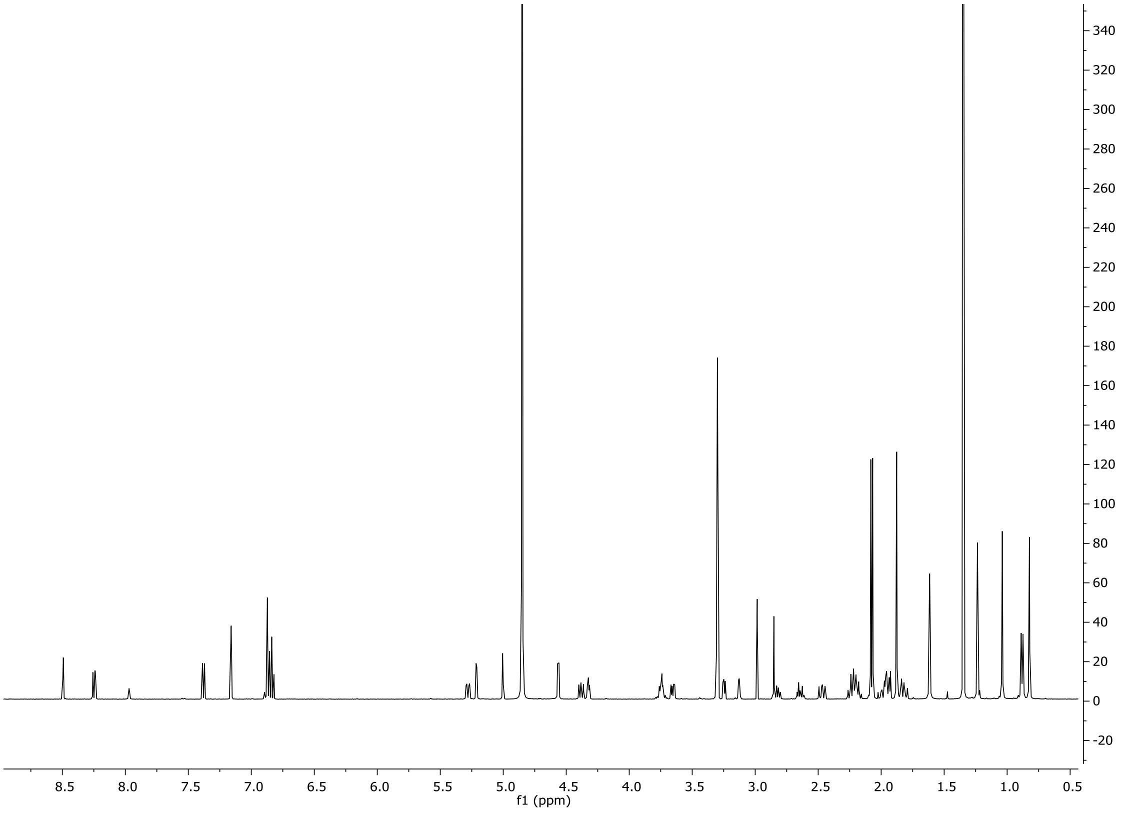

Supplementary Figure 48. ^{1}H NMR spectrum of **7** in methanol-d_4

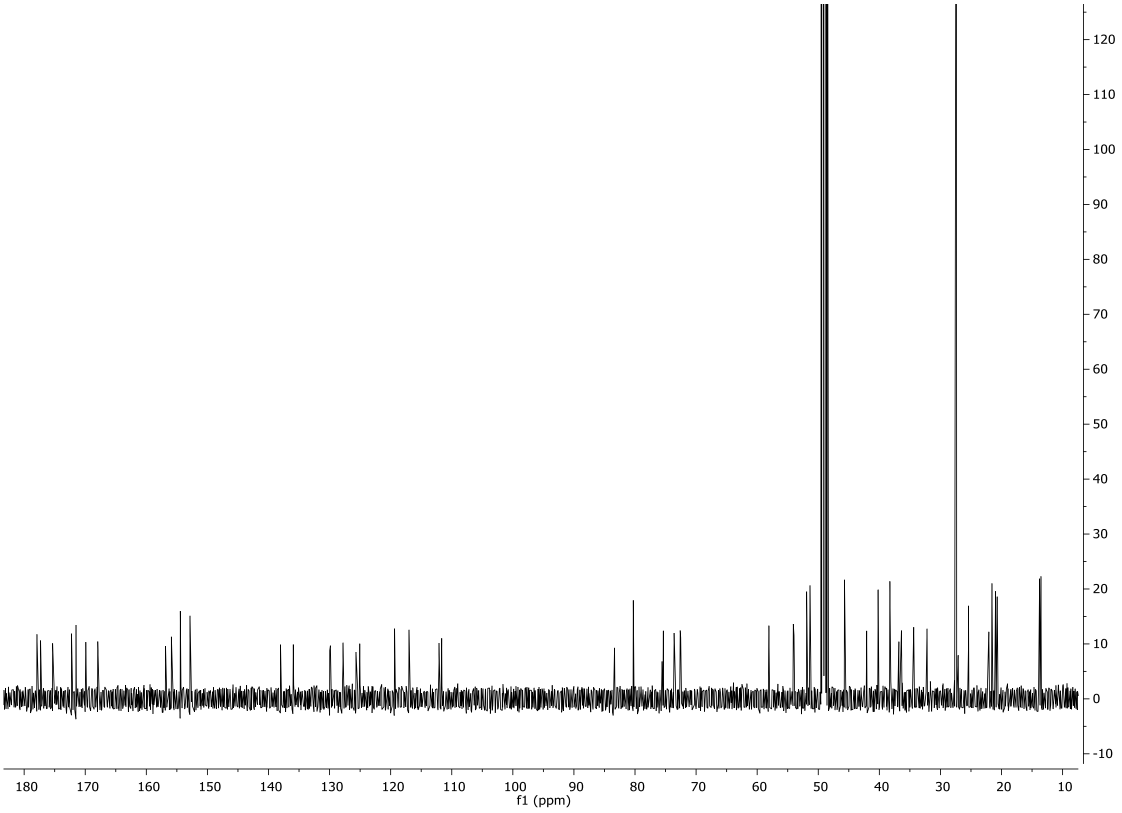

Supplementary Figure 49. ^{13}C NMR spectrum of **7** in methanol-d_4

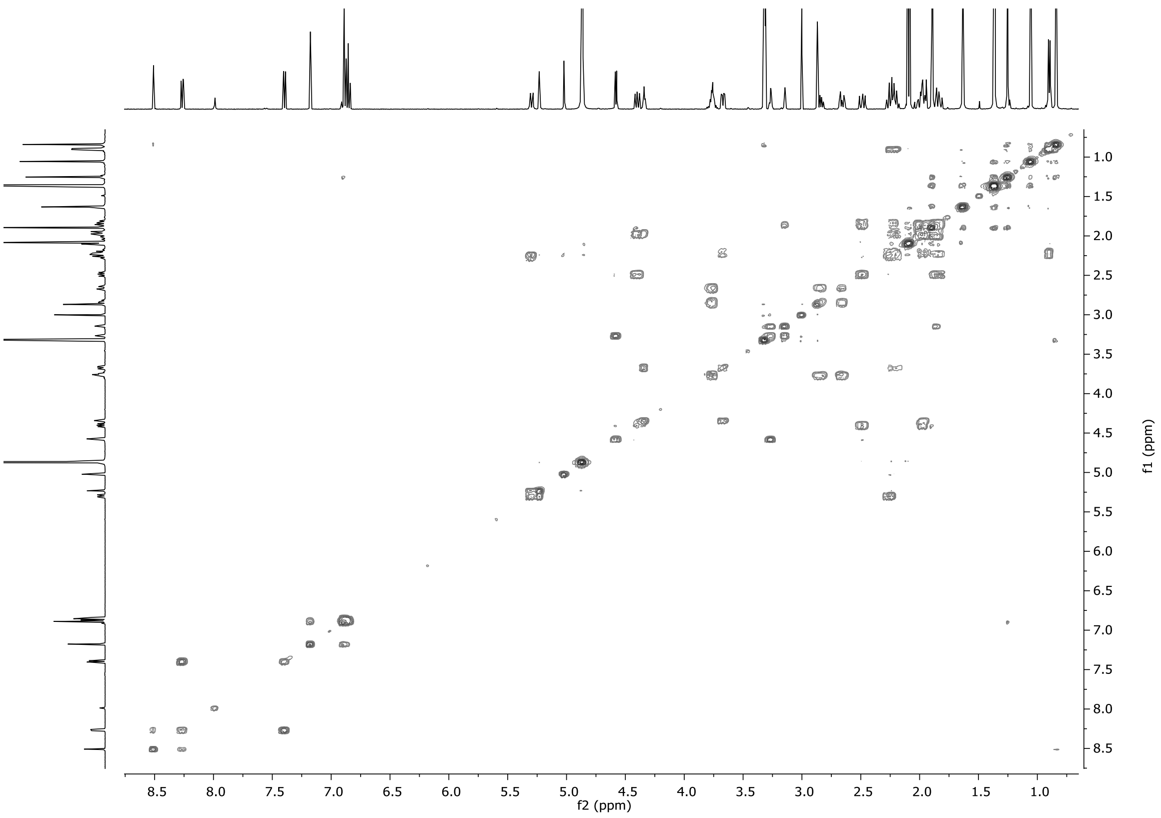

Supplementary Figure 50. ^{1}H-^{1}H COSY spectrum of **7** in methanol-d_4

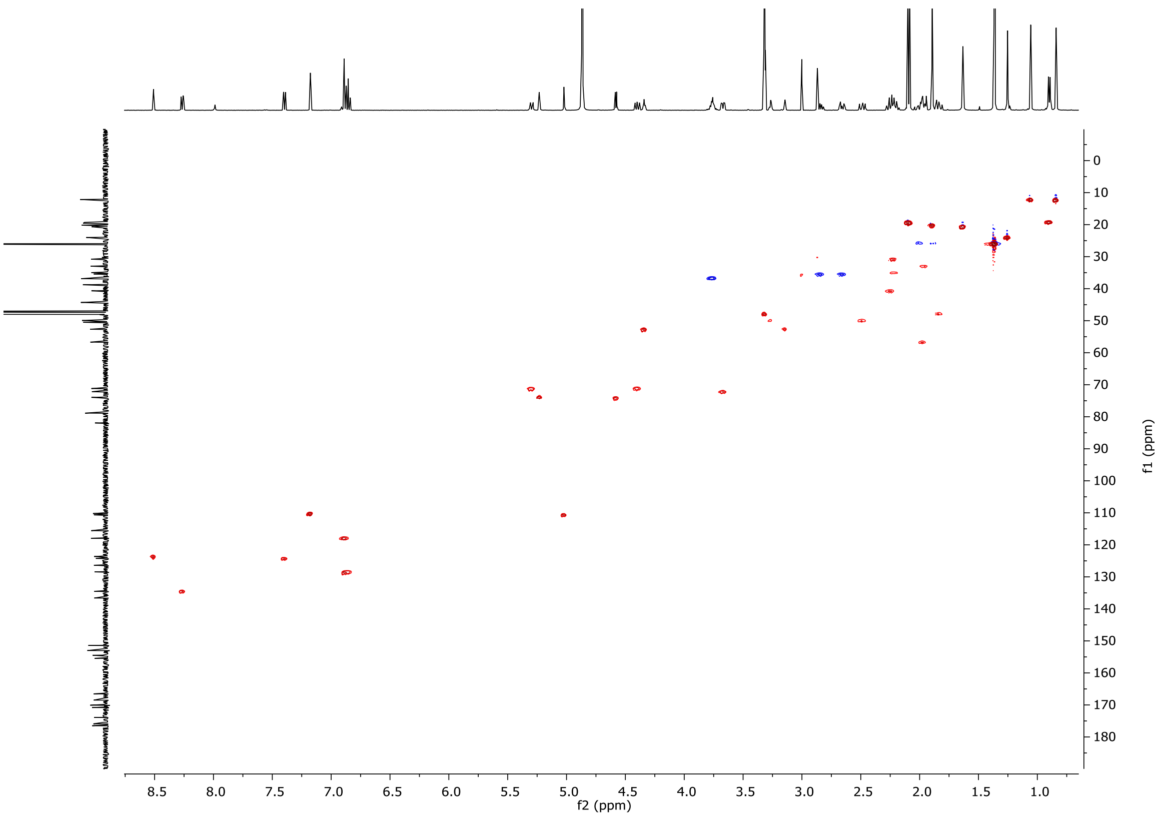

Supplementary Figure 51. HSQC spectrum of **7** in methanol-d_4

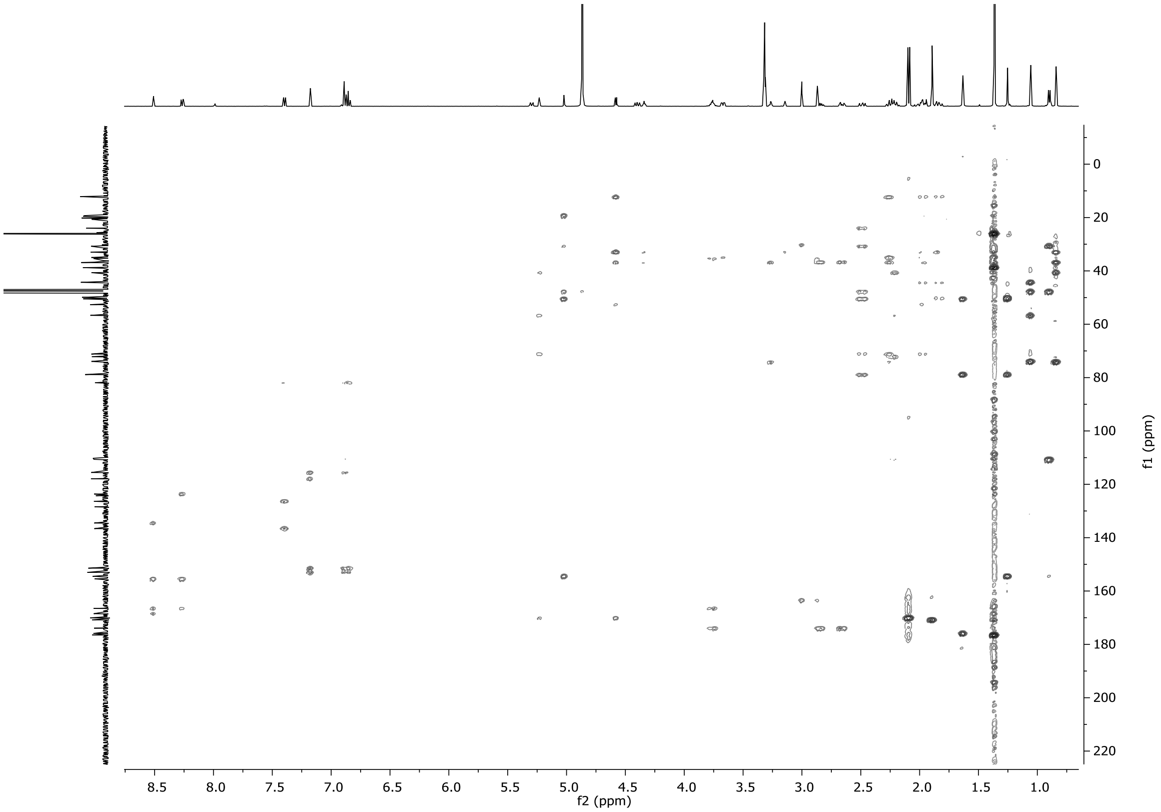

Supplementary Figure 52. HMBC spectrum of **7** in methanol-d_4

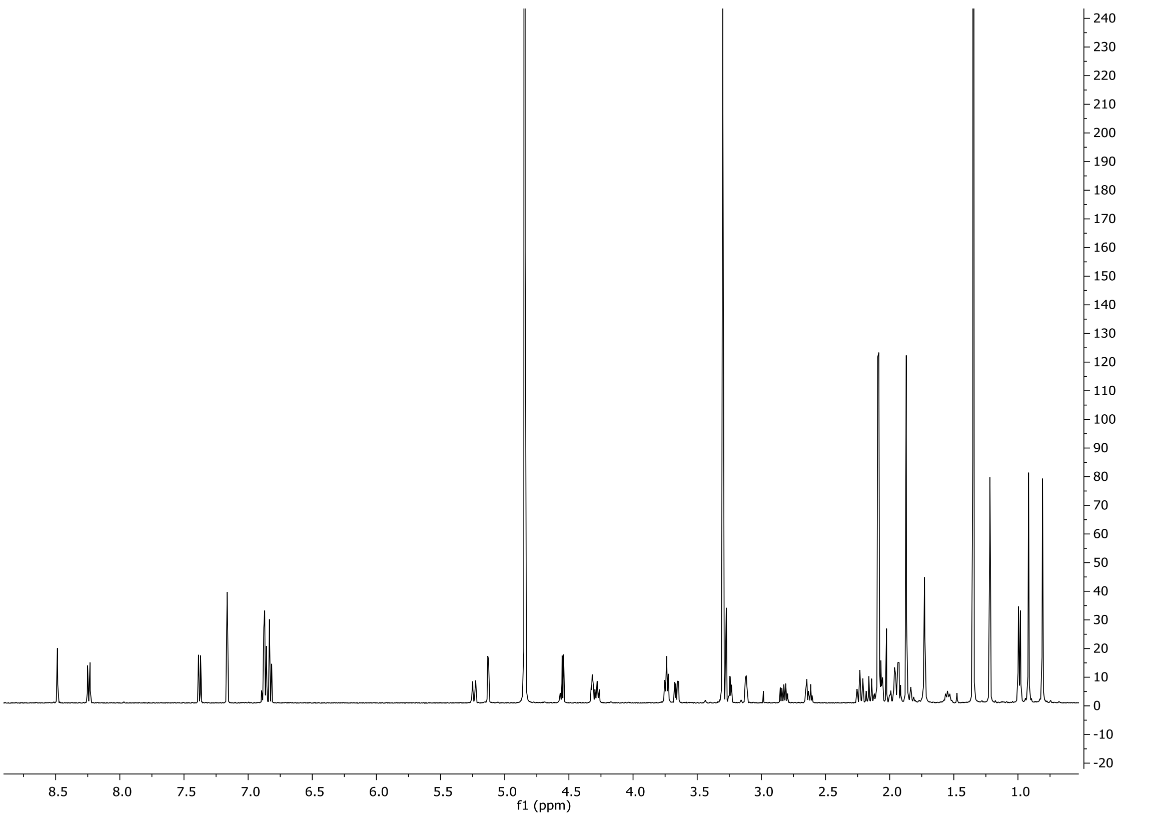

Supplementary Figure 53. ^{1}H NMR spectrum of **8** in methanol-d_4

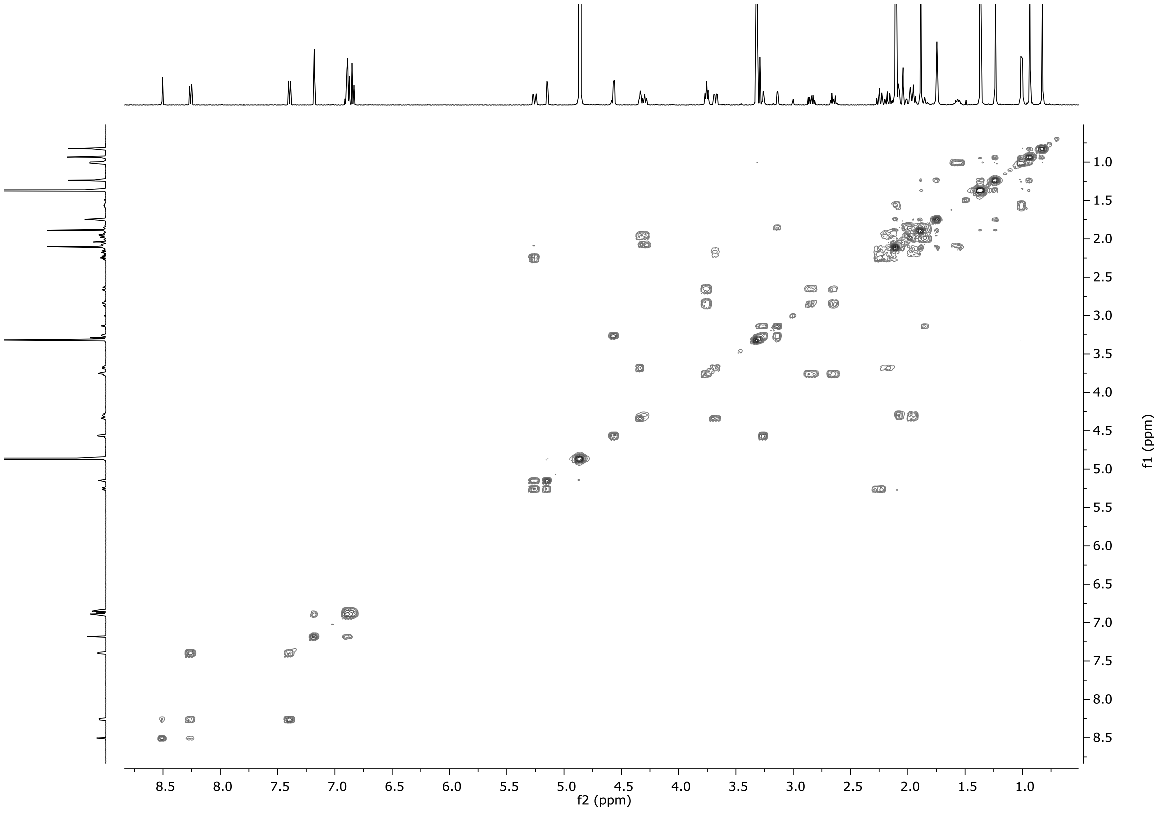

Supplementary Figure 54. ^{1}H-^{1}H COSY spectrum of **8** in methanol-d_4

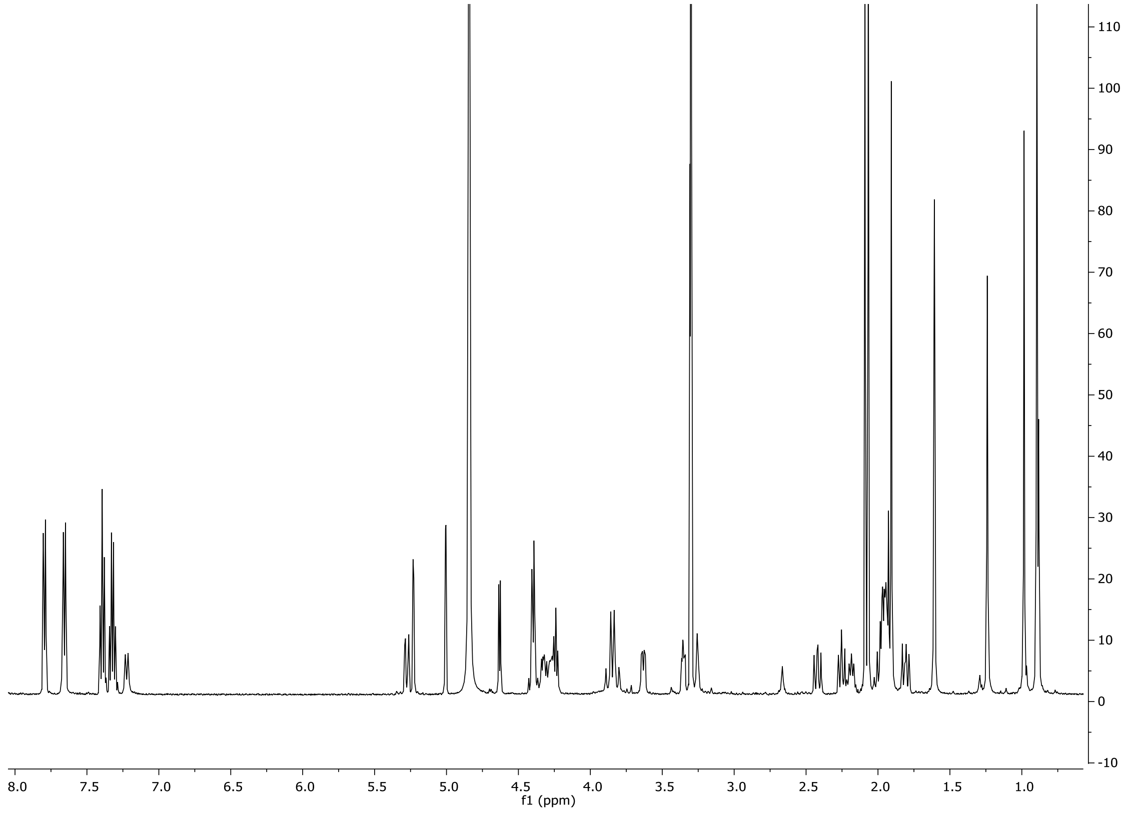

Supplementary Figure 55. ^{1}H NMR spectrum of **26** in methanol-d_4

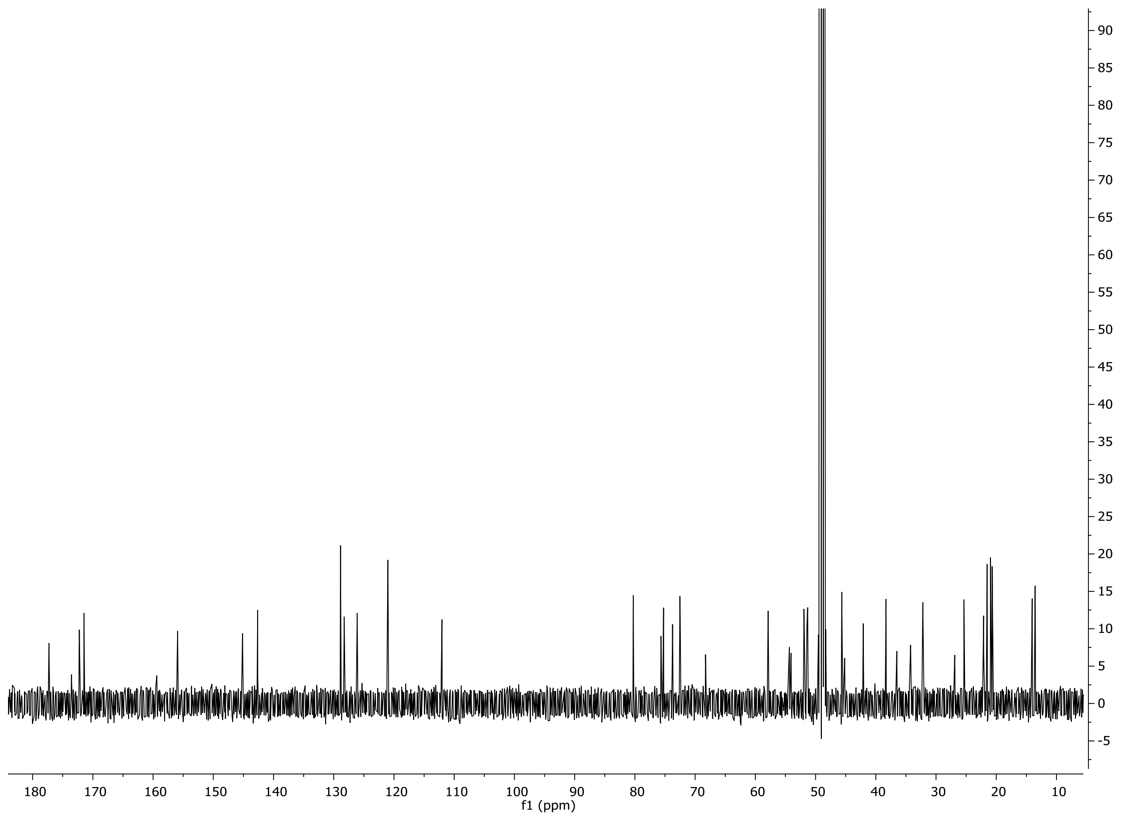

Supplementary Figure 56. ^{13}C NMR spectrum of **26** in methanol-d_4

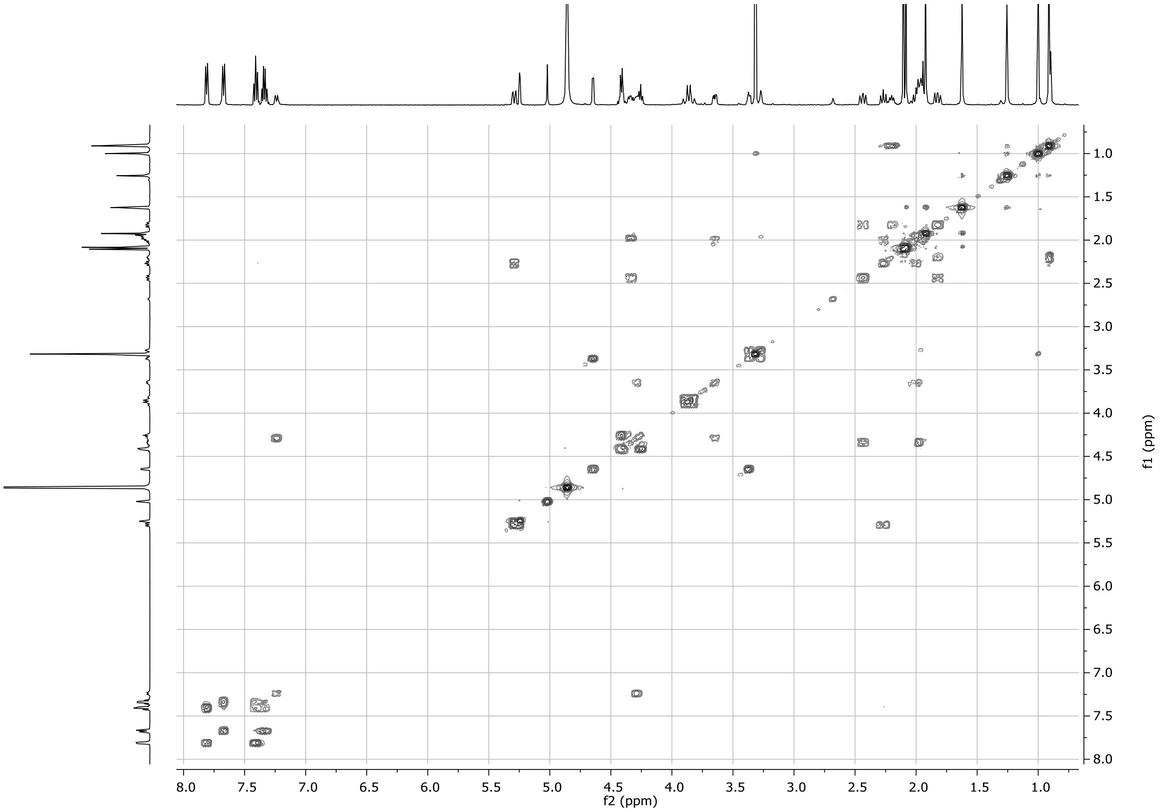

Supplementary Figure 57. ^{1}H-^{1}H COSY spectrum of **26** in methanol-d_4

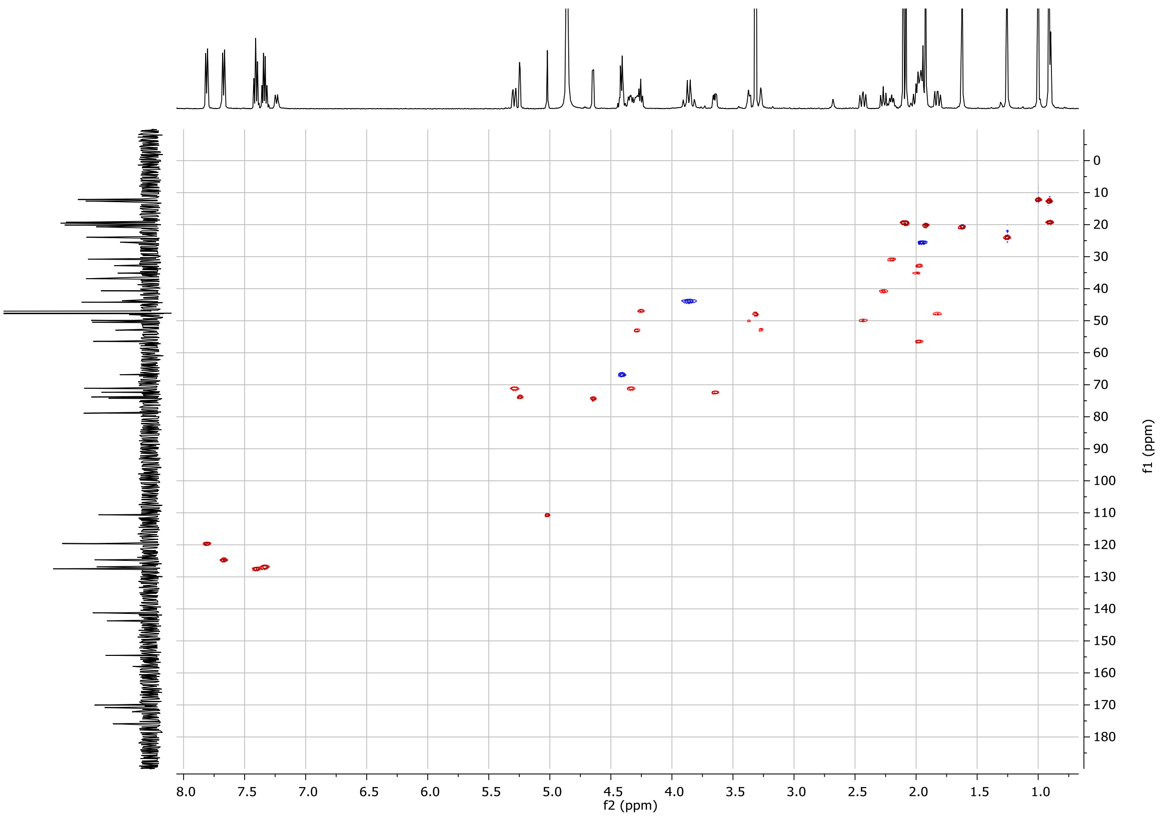

Supplementary Figure 58. HSQC spectrum of **26** in methanol-d_4

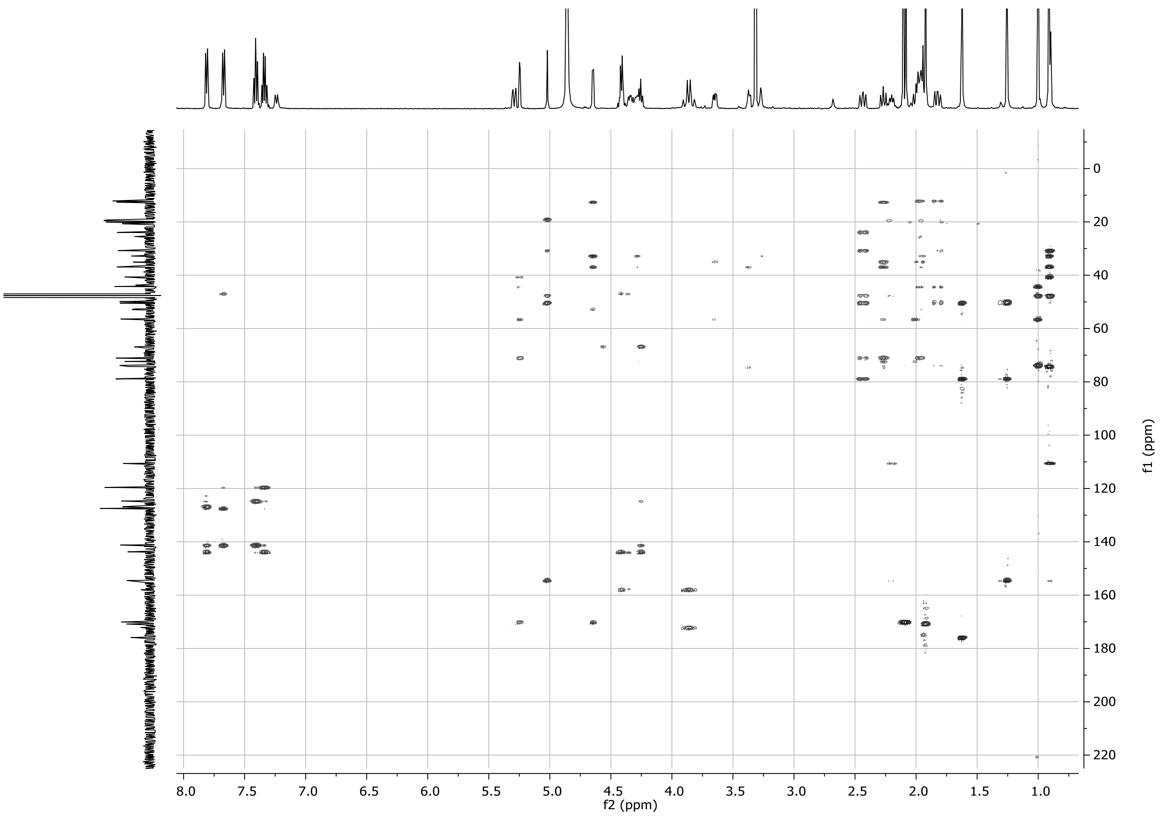

Supplementary Figure 59. HMBC spectrum of **26** in methanol-d_4

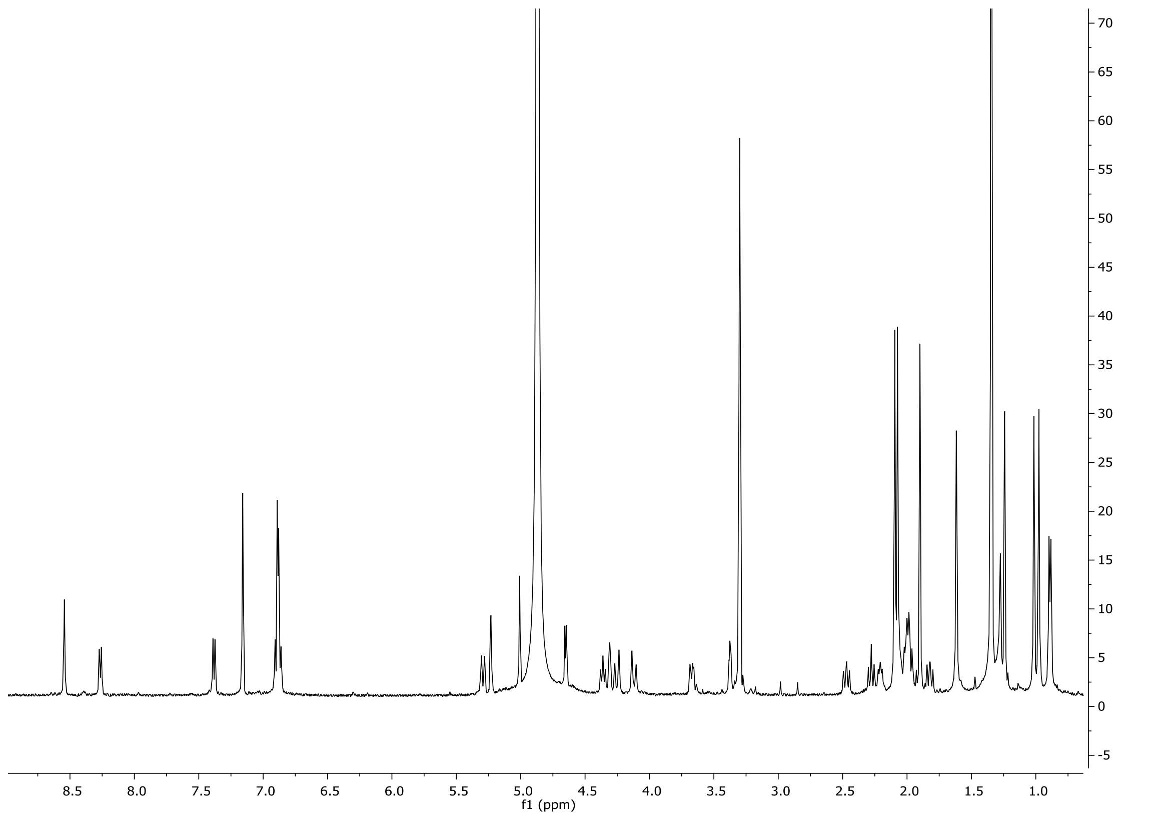

Supplementary Figure 60. ^{1}H NMR spectrum of **27** in methanol-d_4

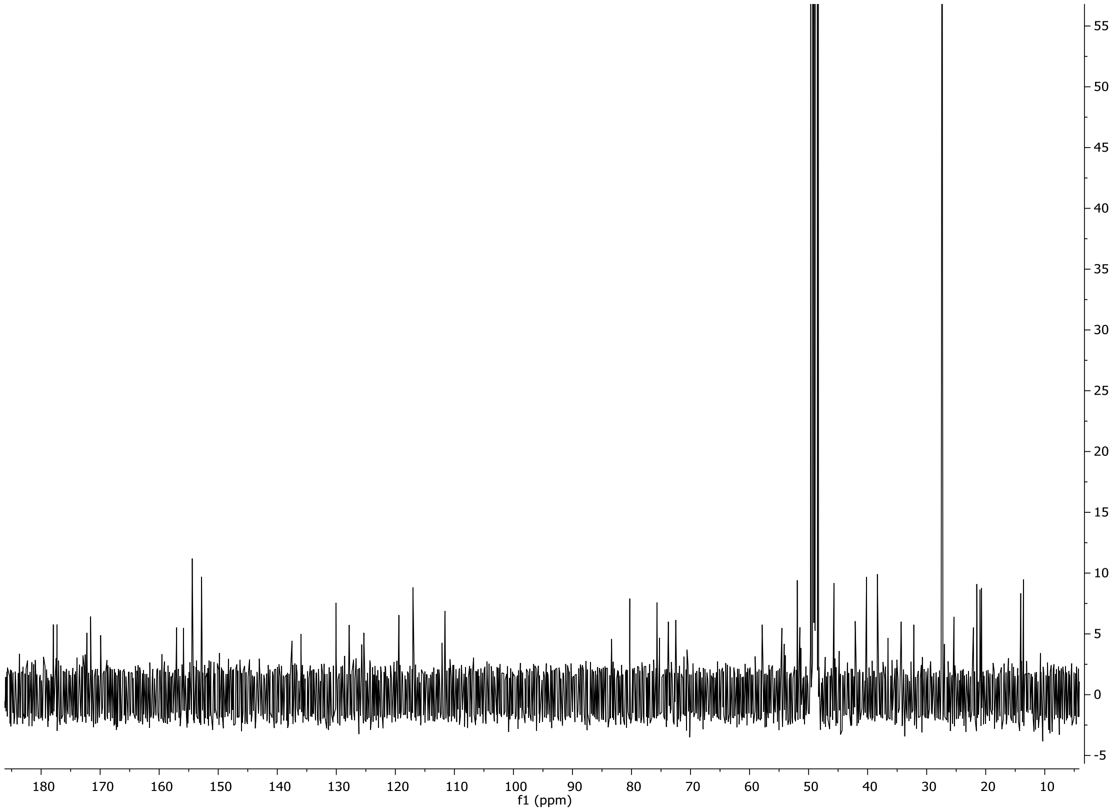

Supplementary Figure 61. ^{13}C NMR spectrum of **27** in methanol-d_4

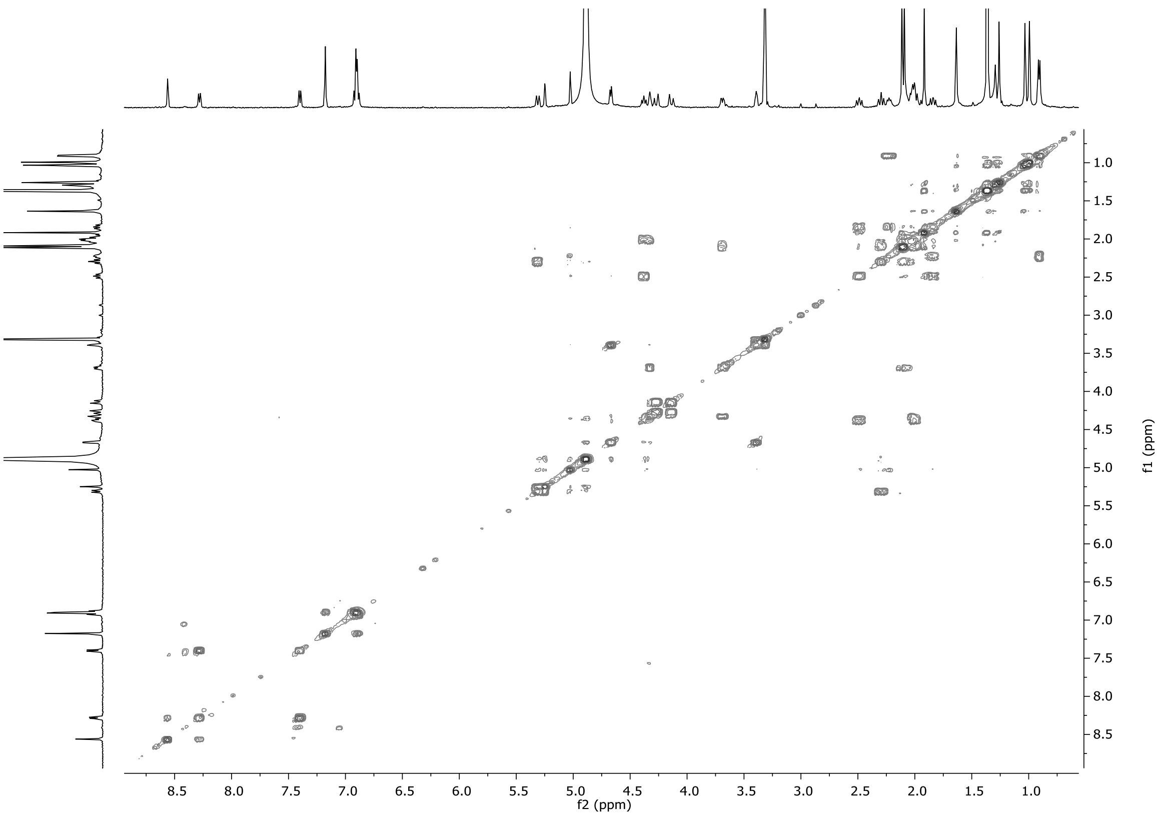

Supplementary Figure 62. ^{1}H-^{1}H COSY spectrum of **27** in methanol-d_4

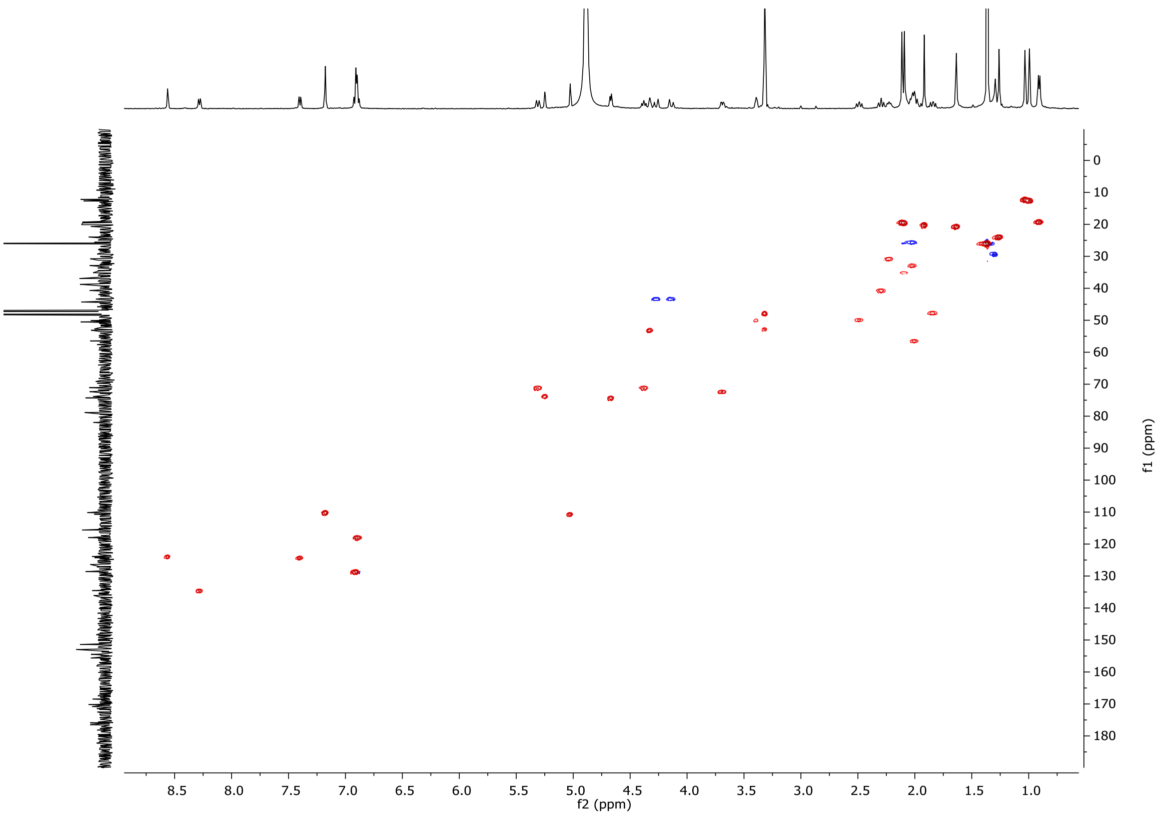

Supplementary Figure 63. HSQC spectrum of **27** in methanol-d_4

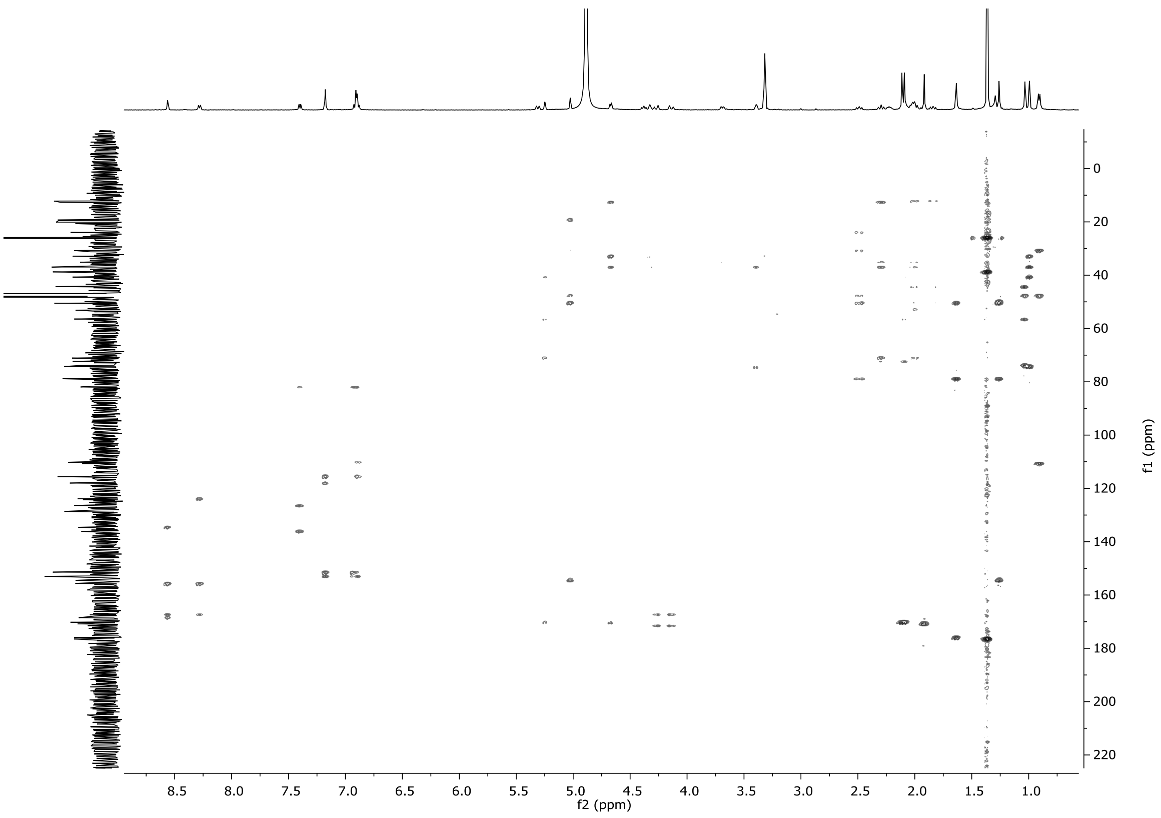

Supplementary Figure 64. HMBC spectrum of **27** in methanol-d_4

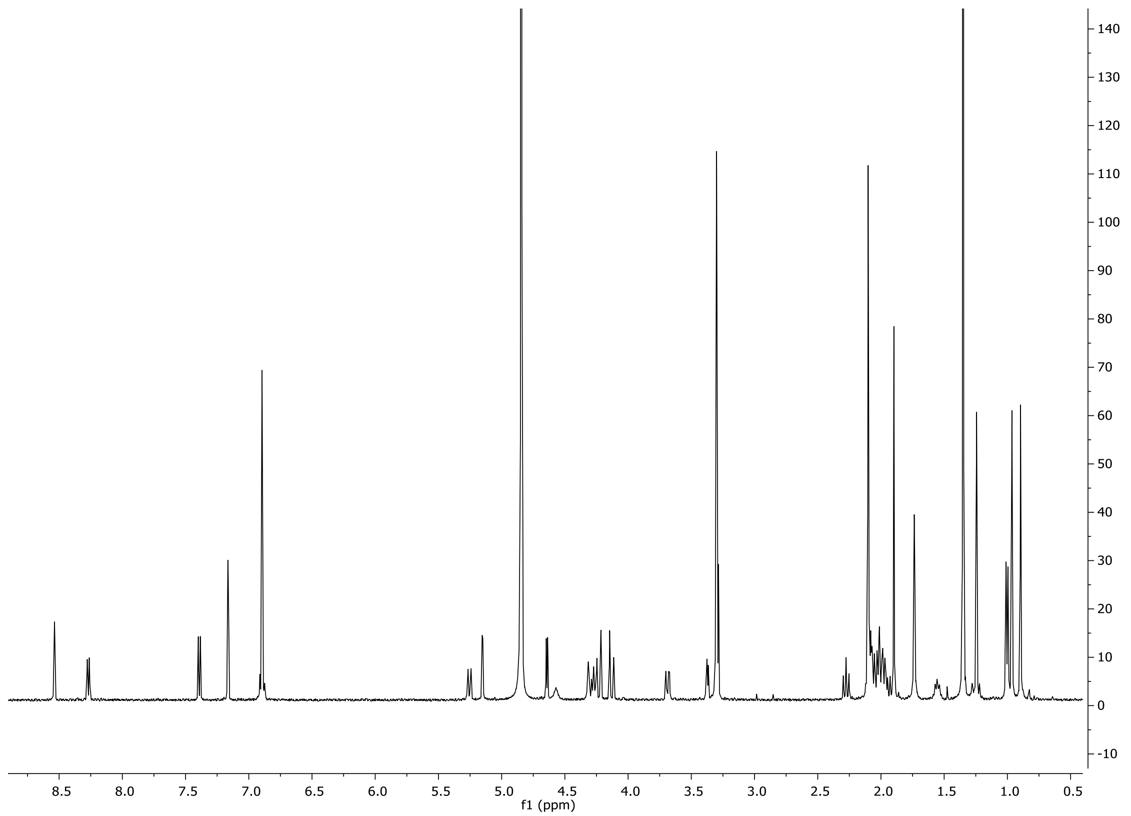

Supplementary Figure 65. ^{1}H NMR spectrum of **9** in methanol-d_4

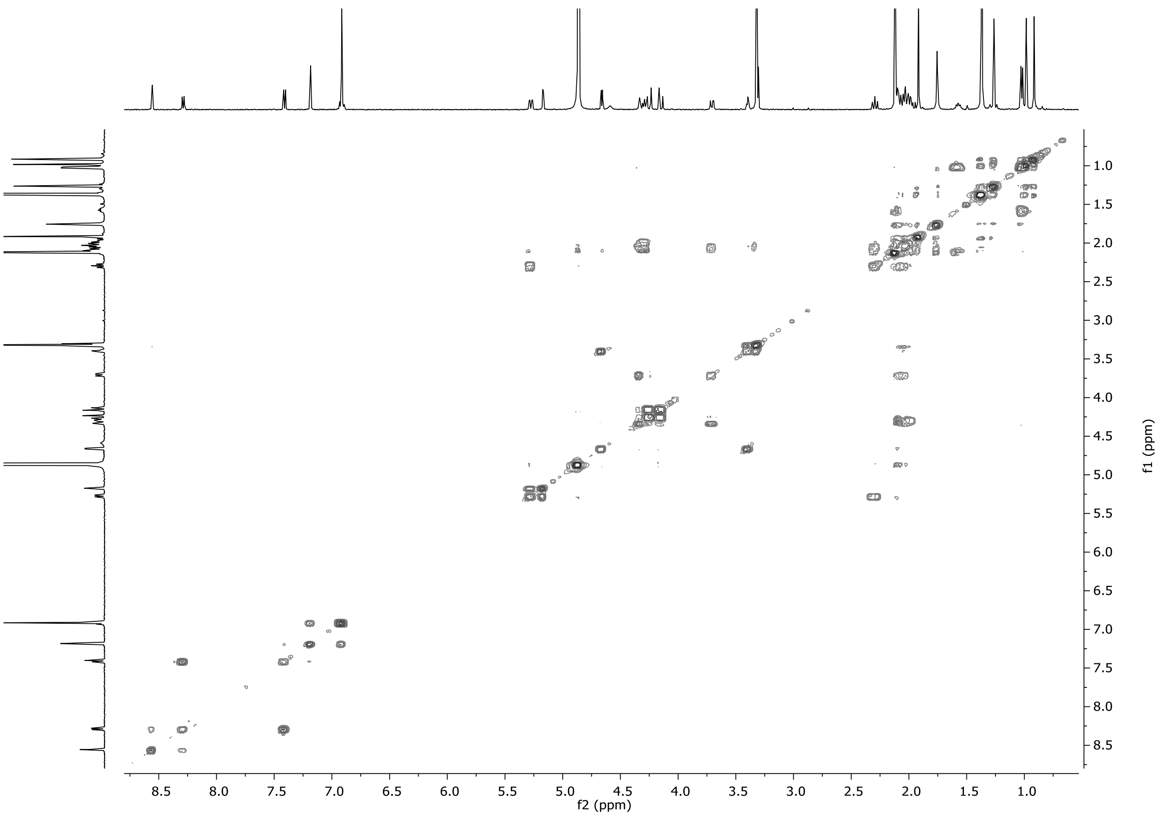

Supplementary Figure 66. ^{1}H-^{1}H COSY spectrum of **9** in methanol-*d*$_4$

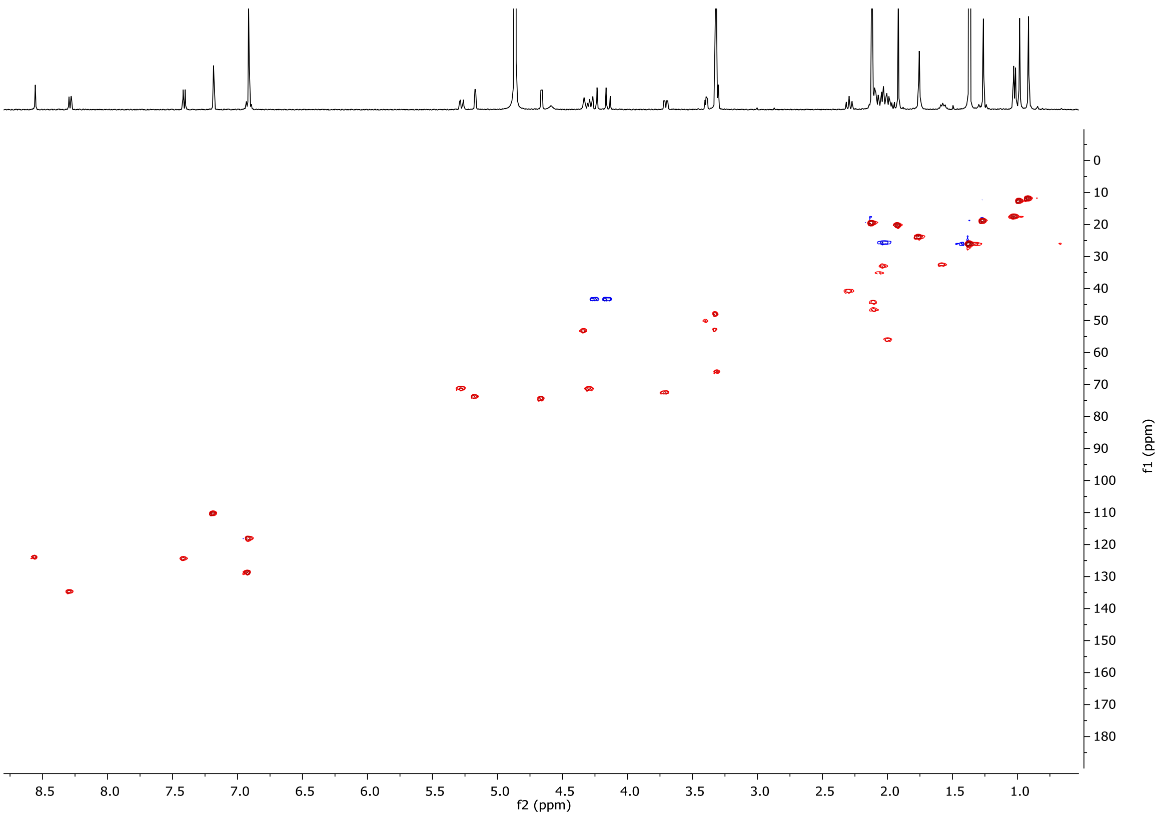

Supplementary Figure 67. HSQC spectrum of **9** in methanol-d_4

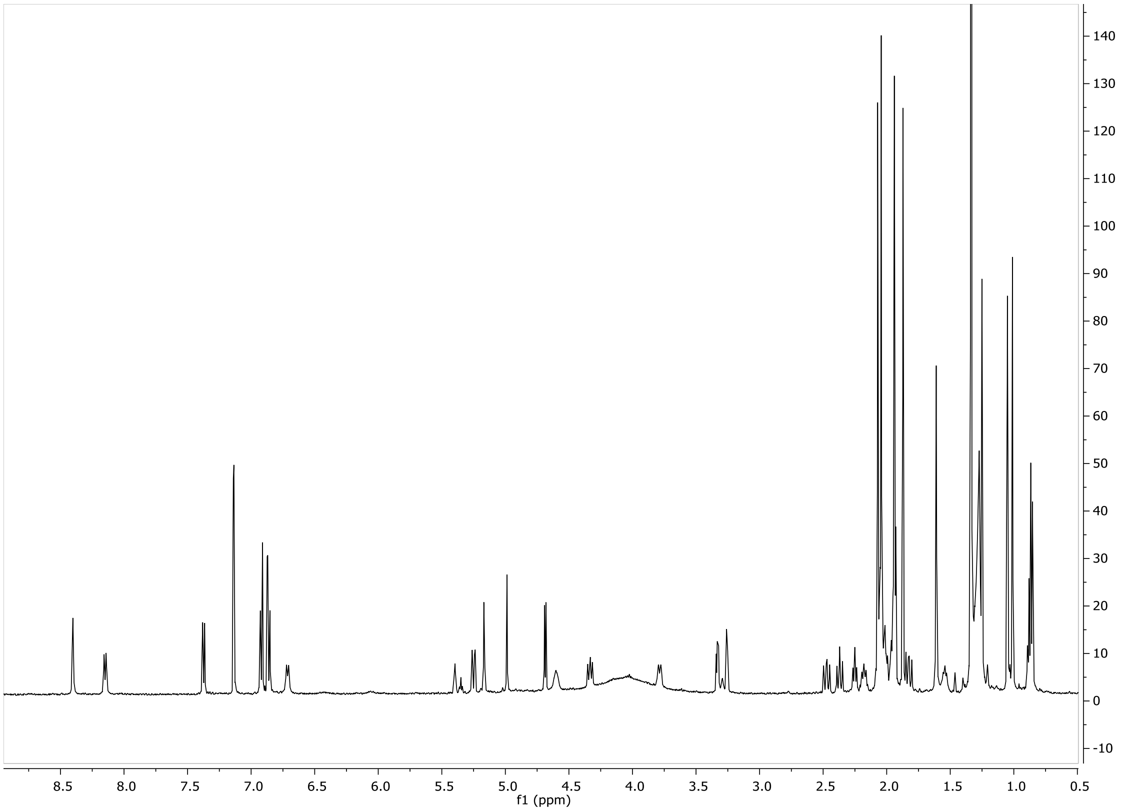

Supplementary Figure 68. ^{1}H NMR spectrum of **10** in acetonitrile-d_3

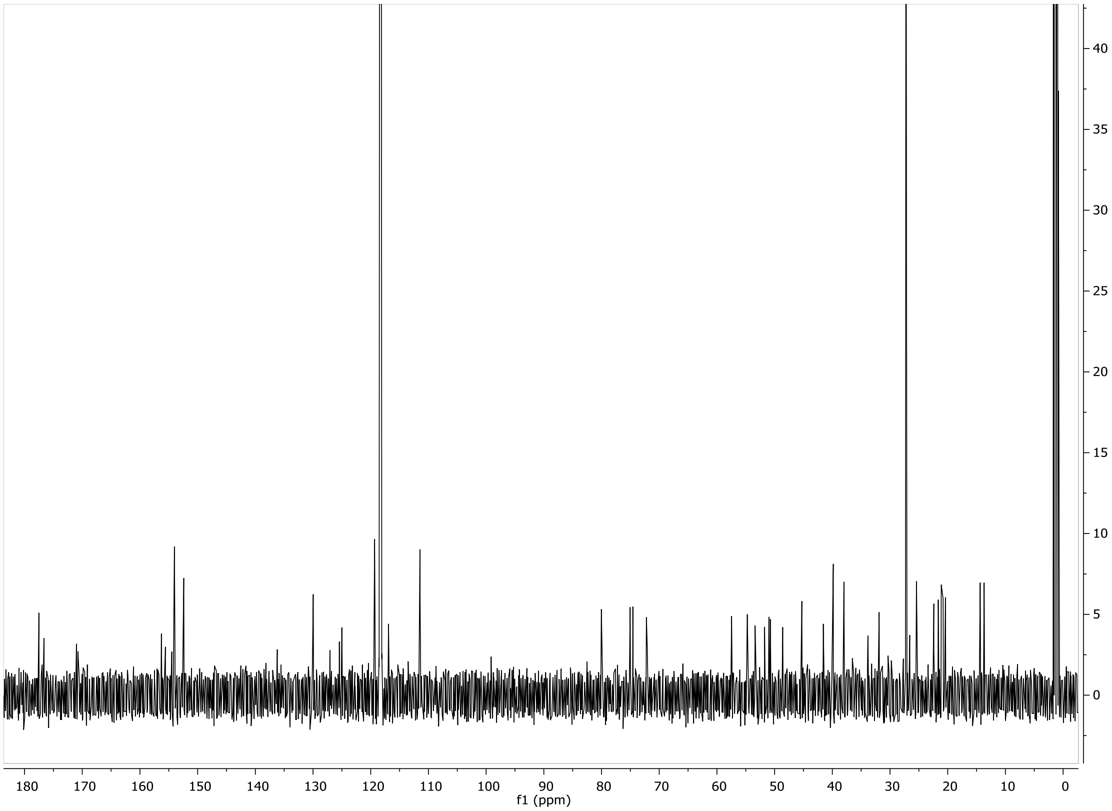

Supplementary Figure 69. ^{13}C NMR spectrum of **10** in acetonitrile-d_3

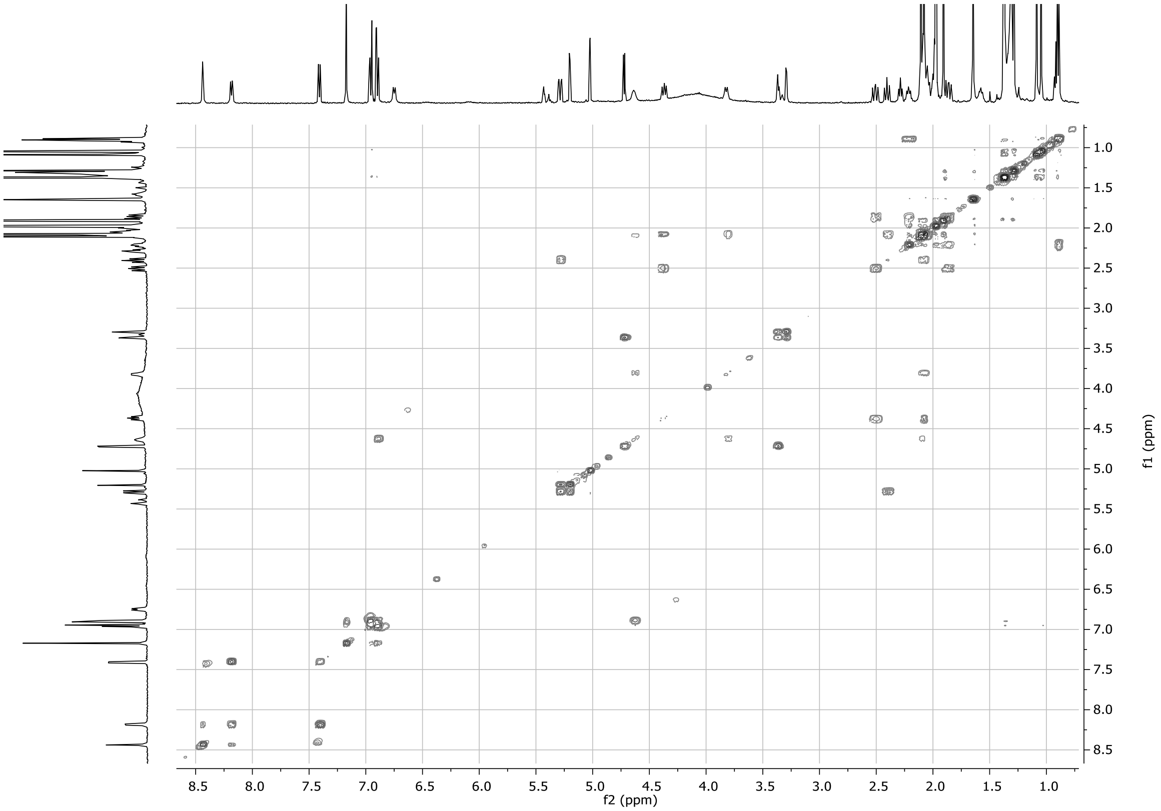

Supplementary Figure 70. ^{1}H-^{1}H COSY spectrum of **10** in acetonitrile-*d*$_3$

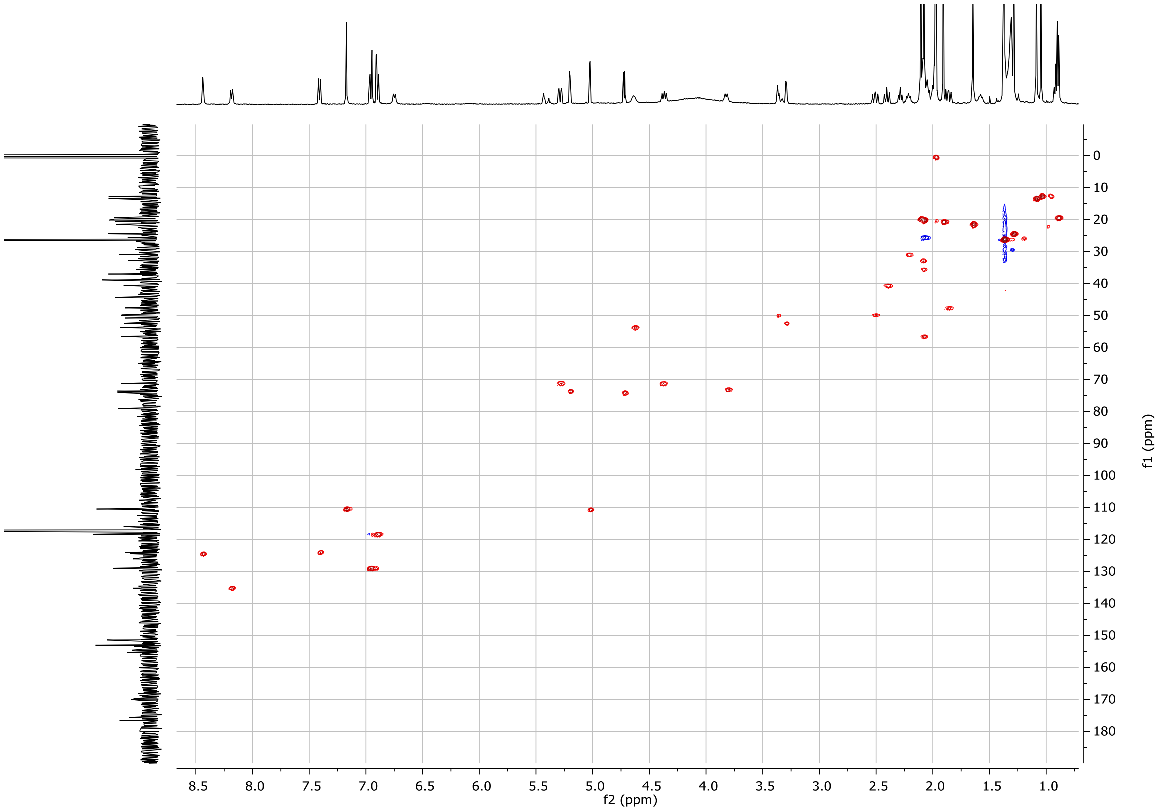

Supplementary Figure 71. HSQC spectrum of **10** in acetonitrile-d_3

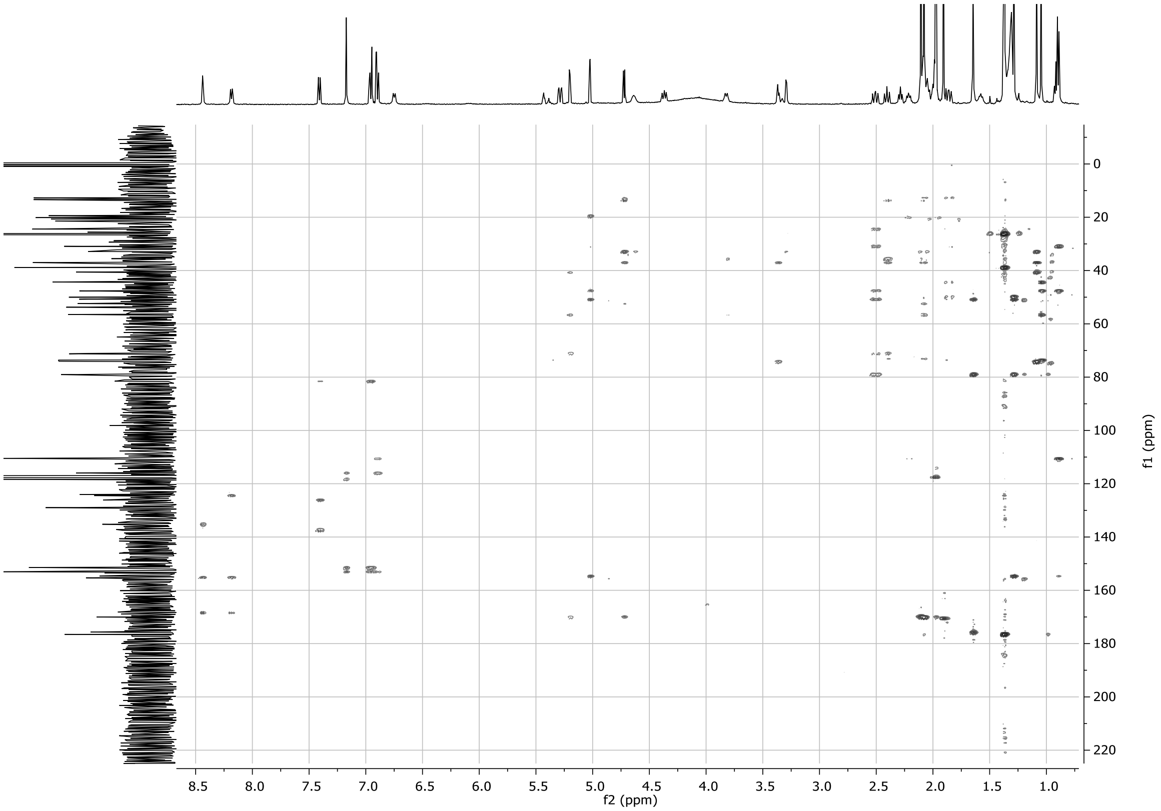

Supplementary Figure 72. HMBC spectrum of **10** in acetonitrile-d_3

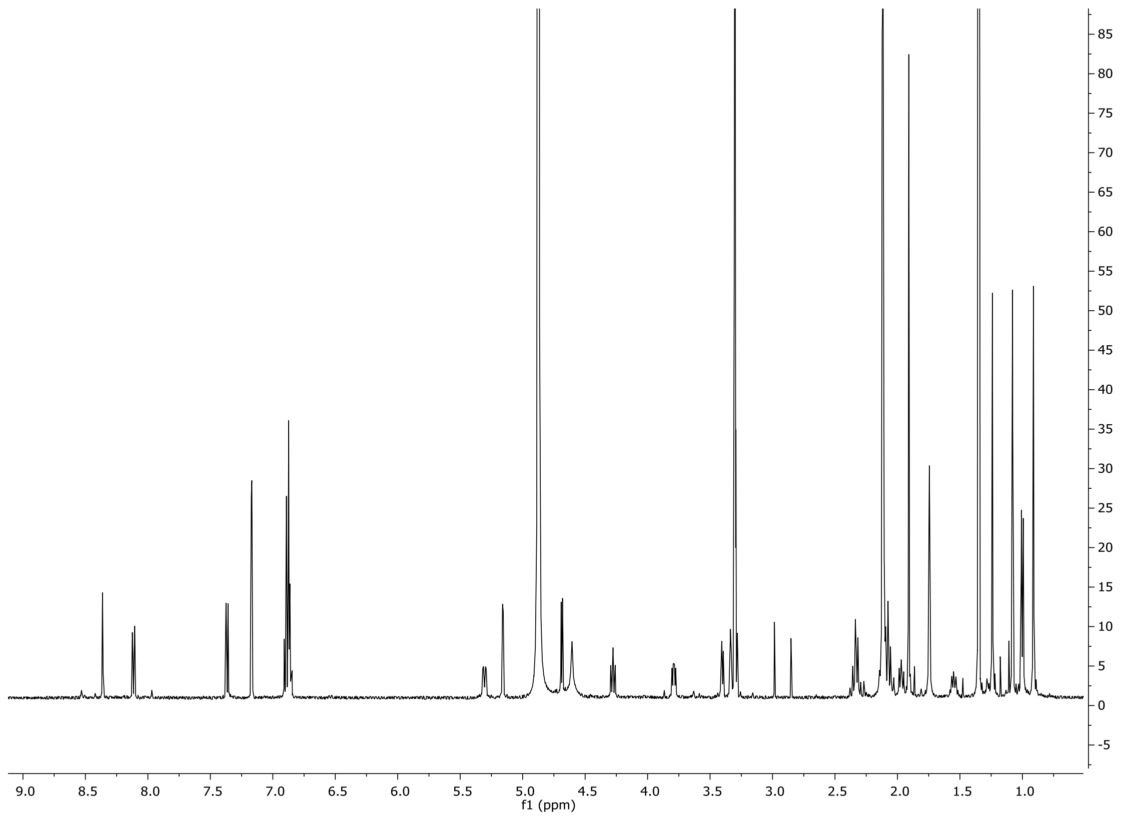

Supplementary Figure 73. ^{1}H NMR spectrum of **11** in methanol-d_4

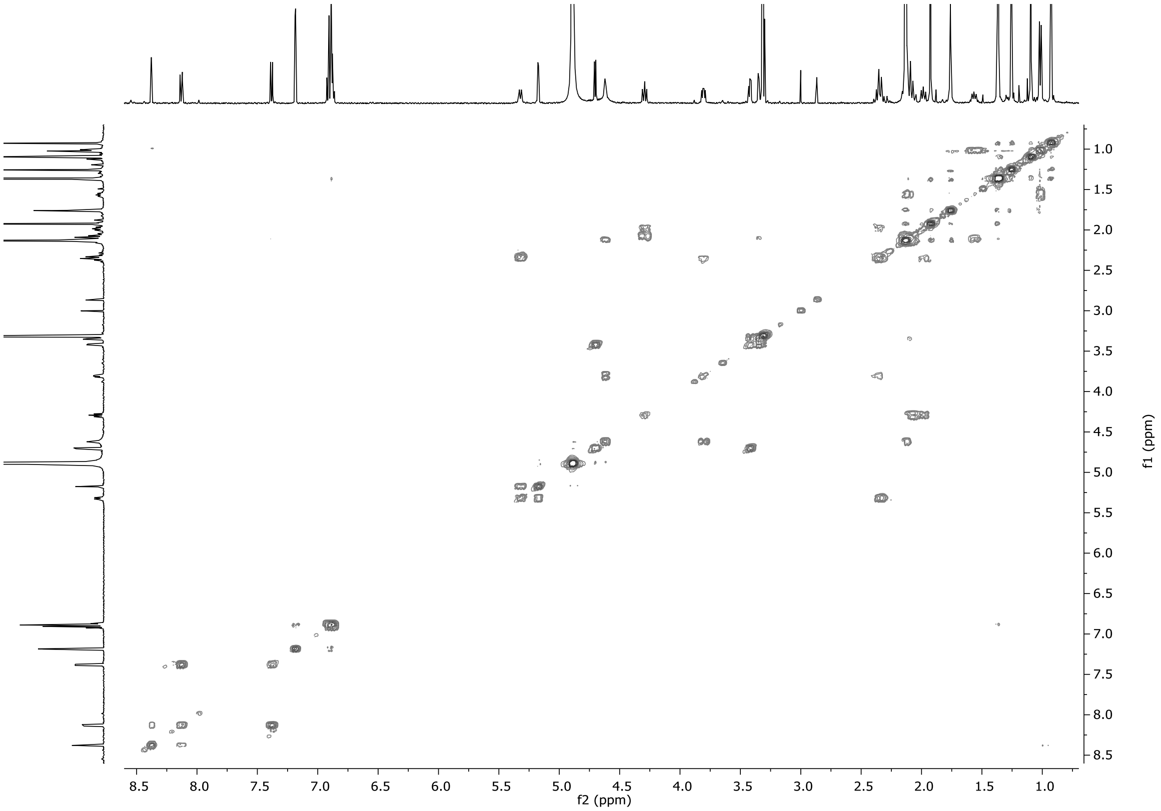

Supplementary Figure 74. ^{1}H-^{1}H COSY spectrum of **11** in methanol-d_4

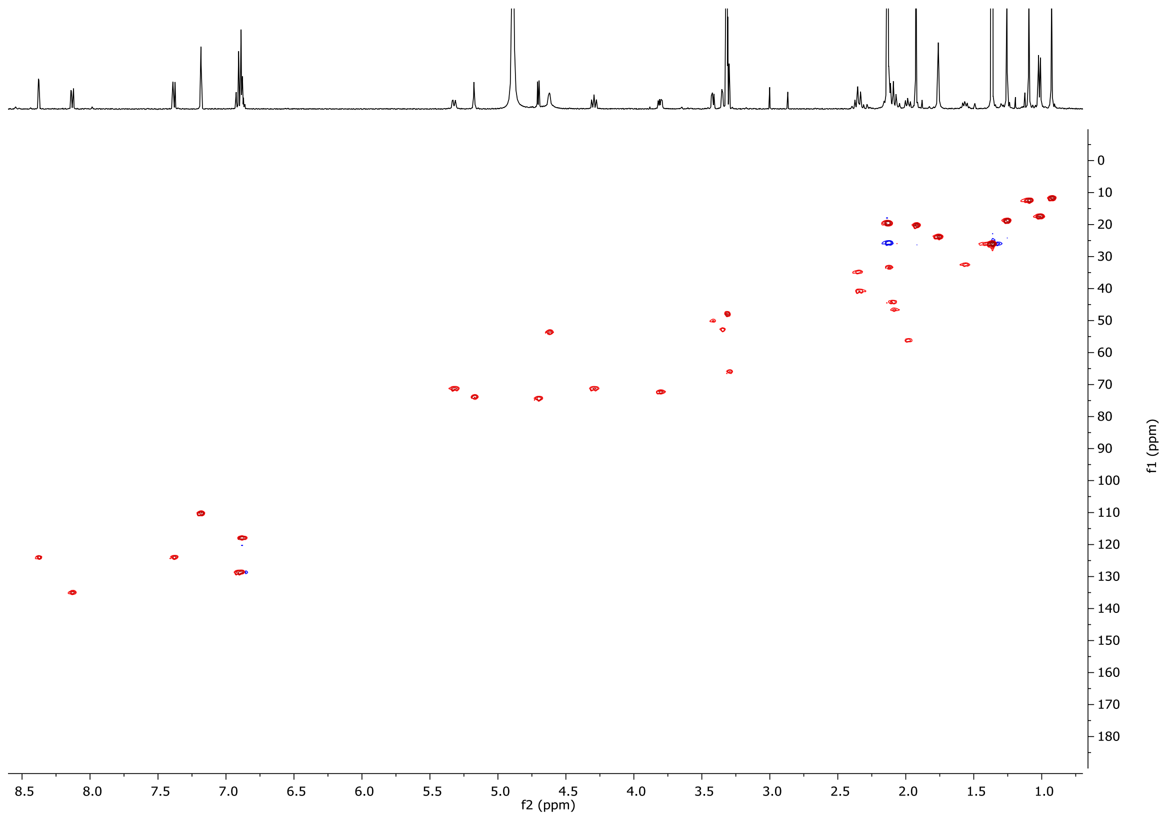

Supplementary Figure 75. HSQC spectrum of **11** in methanol-d_4

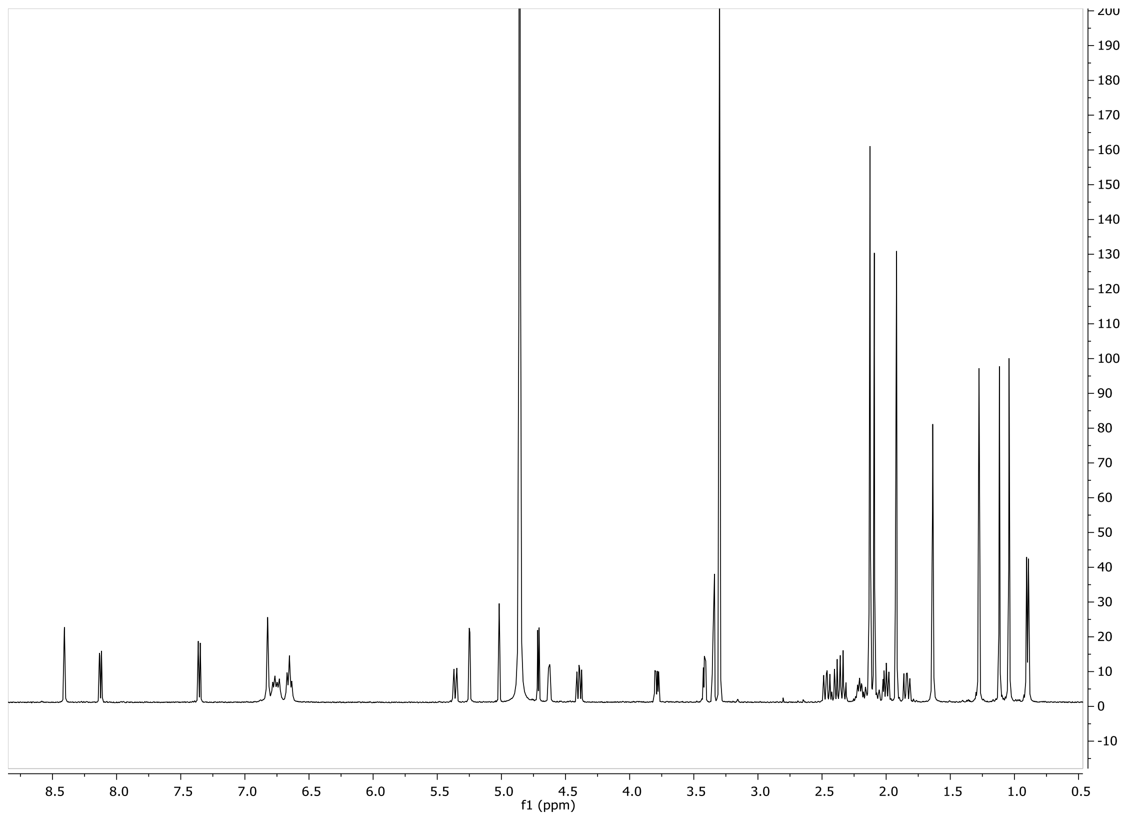

Supplementary Figure 76. ^{1}H NMR spectrum of **28** in methanol-d_4

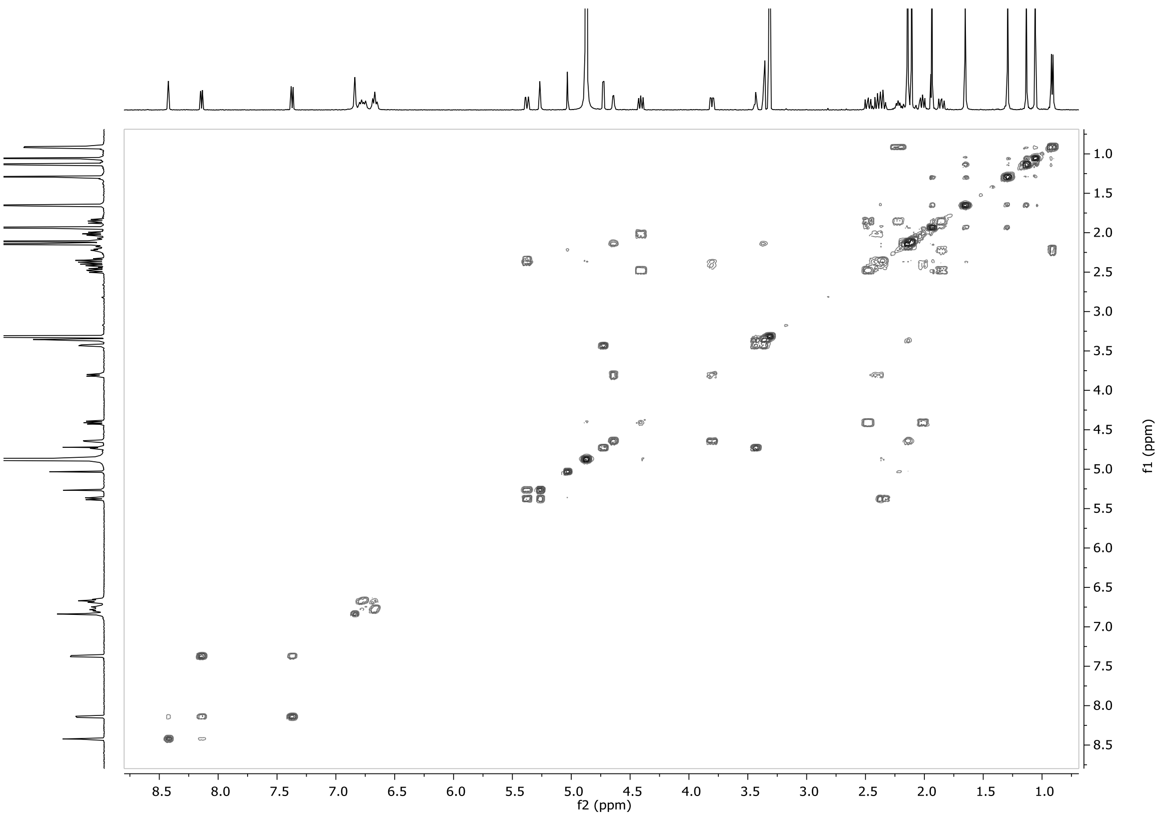

Supplementary Figure 77. ^{1}H-^{1}H COSY spectrum of **28** in methanol-d_4

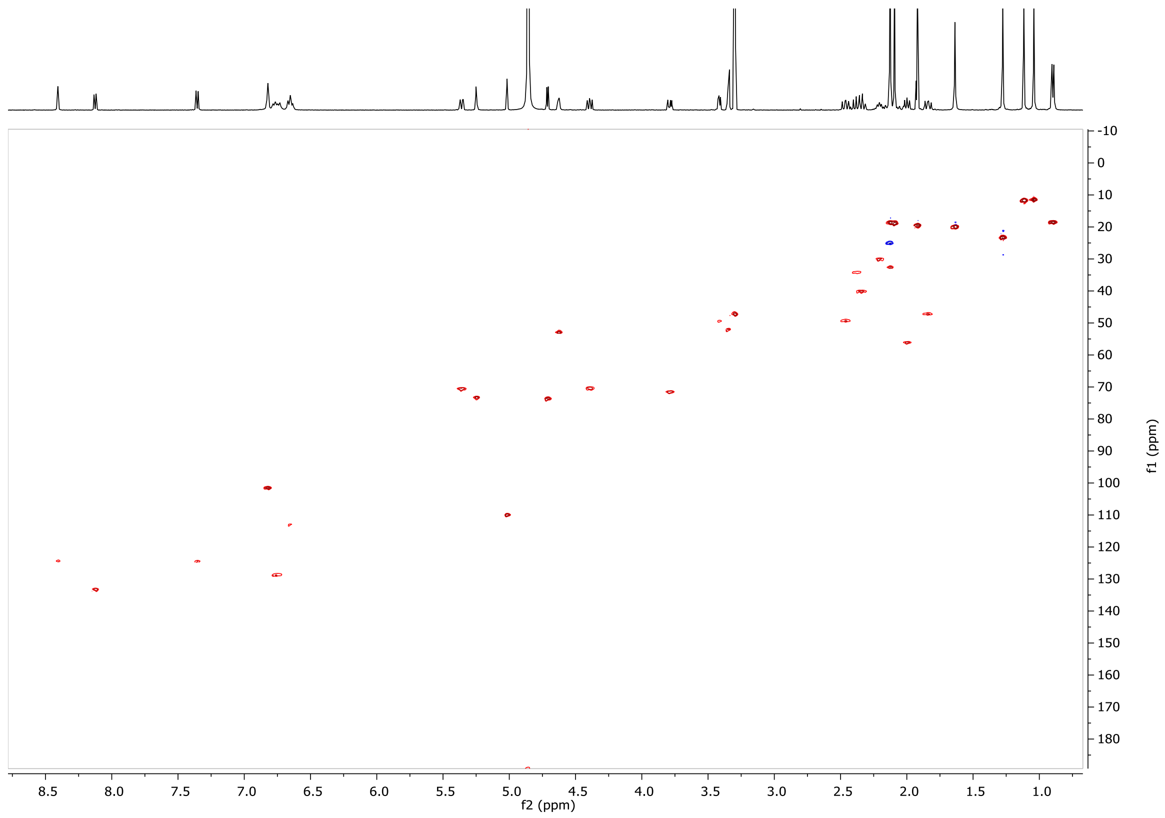

Supplementary Figure 78. HSQC spectrum of **28** in methanol-d_4

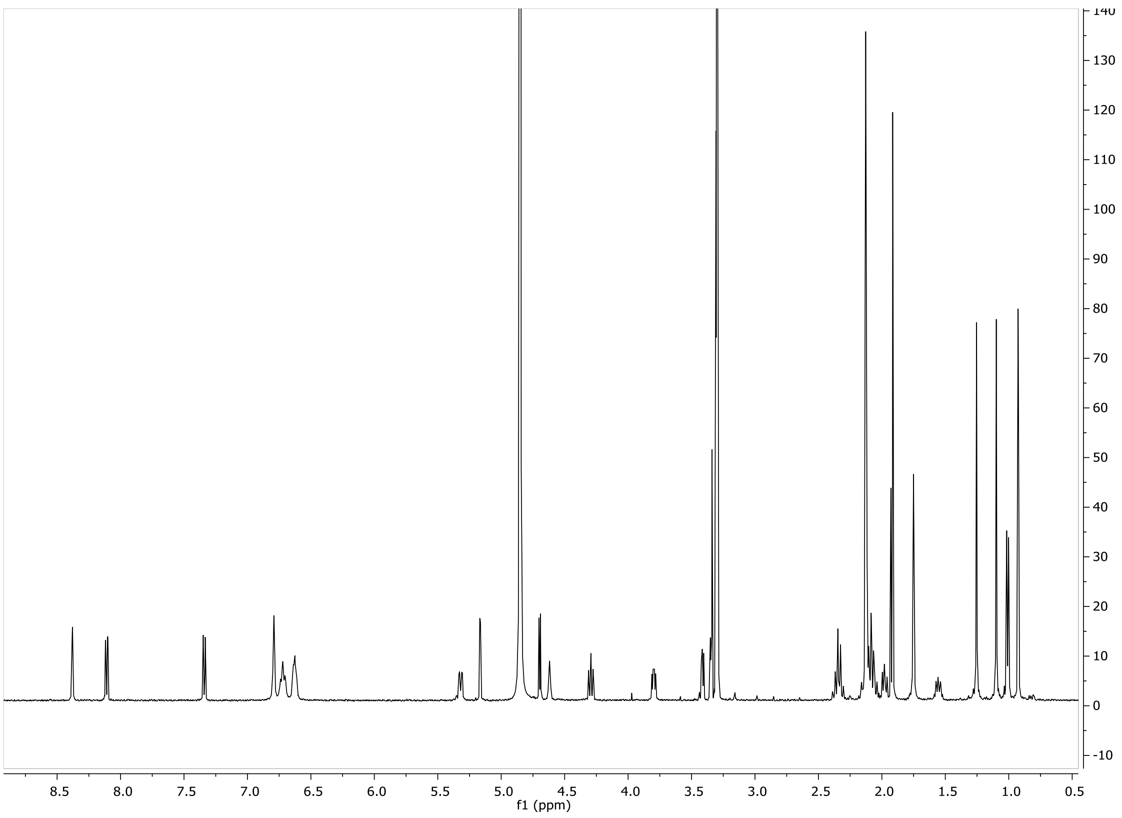

Supplementary Figure 79. ^{1}H NMR spectrum of **12** in methanol-d_4

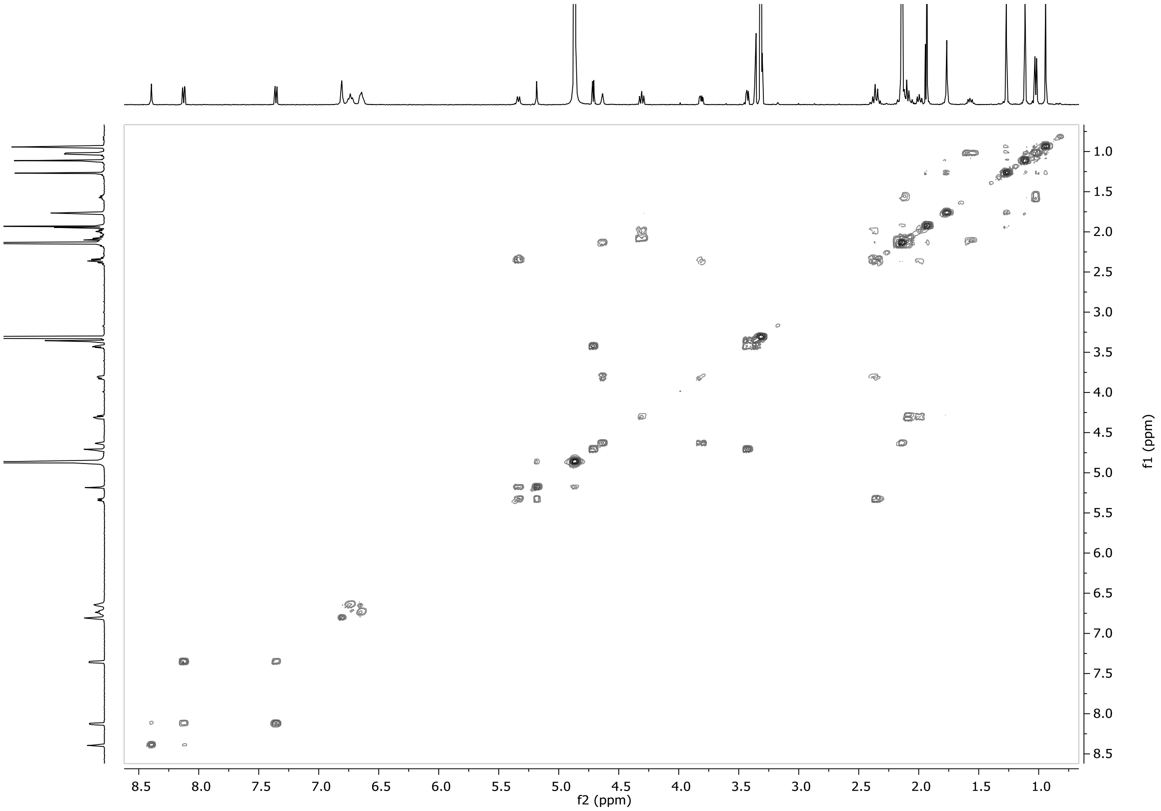

Supplementary Figure 80. ^{1}H-^{1}H COSY spectrum of **12** in methanol-*d*$_4$

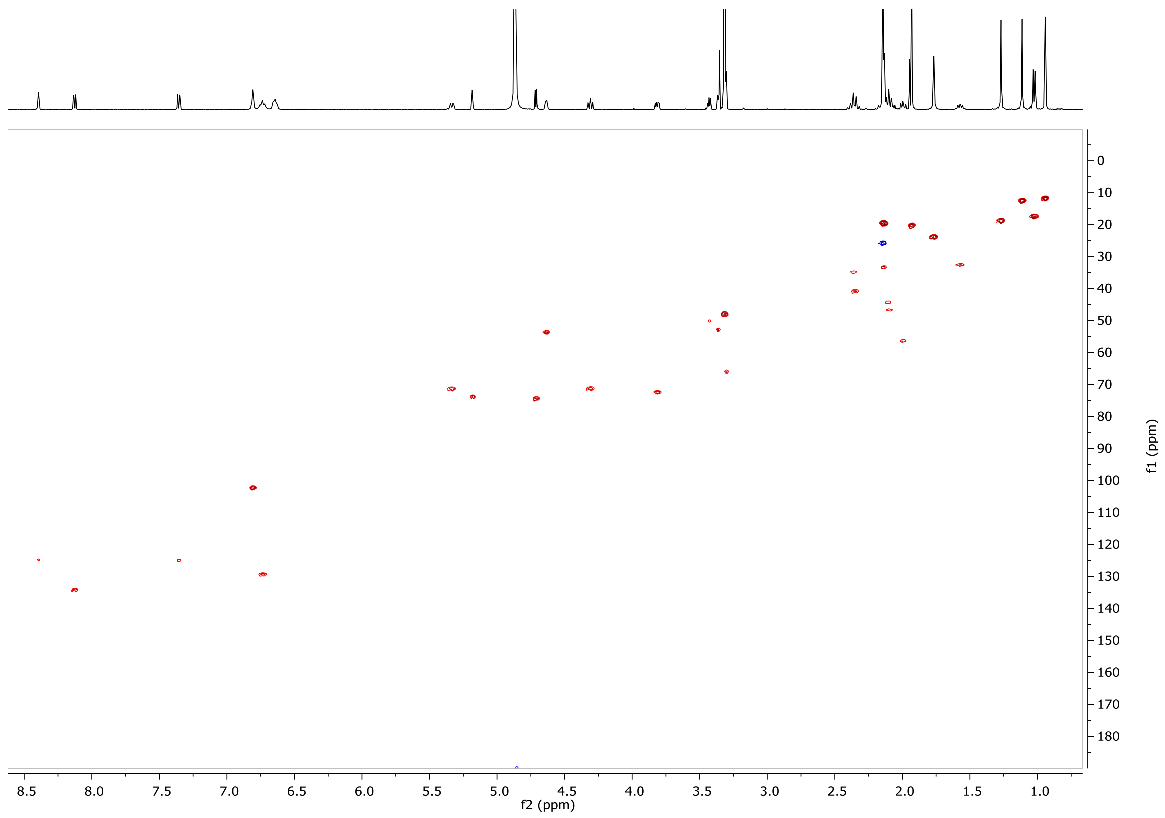

Supplementary Figure 81. HSQC spectrum of **12** in methanol-d_4

Supplementary Tables

Supplementary Table 1. ^{1}H (400 MHz) and ^{13}C (100 NHz) NMR data of TFA-14 (Methanol-*d*$_4$)

No.	6S-^{13}C	6S-^{1}H (*J*)	6R-^{13}C	6R-^{1}H (*J*)
1	75.2	4.69, d (5.4)	73.9	4.74, d (5.6)
2	51.2	3.43, m	51.1	3.47, m
3	53.4	3.40, m	52.9	3.38, m
4	26.4	2.19, m	26.6	2.25, m
		2.02, m		1.83, m
5	32.1	2.17, m	34.2	1.90, m
6	57.8	3.46, m	58.0	2.97, t (11.6)
7	71.6	3.78, m	74.3	3.32, m
8	35.9	2.05, m	40.1	1.88, m
9	41.5	2.34, m	41.6	2.31, m
10	37.9		39.4	
11	72.3	5.30, dd (11.6, 2.9)	72.1	5.32, dd (11.7, 2.8)
12	75.0	5.25, m	75.2	5.24, m
13	45.6		45.2	
14	57.3	2.05, m	57.8	2.00, m
15	72.1	4.49, m	72.1	4.49, m
16	51.9	2.50, dd (9.9, 13.6)	51.6	2.51, dd (10.0, 13.5)
17	49.2	1.82, m	49.2	1.82, m
18	13.5	1.03, s	13.3	1.00, s
19	13.8	0.97, s	13.8	0.97, s
20	20.6	0.90, d (7.1)	20.5	0.89, d (7.1)
21	31.9	2.23, m	31.9	2.23, m
22	112.1	5.04, m	112.1	5.04, m
23	156.4		156.6	
24	51.3		51.5	
25	80.1		80.1	
26	177.5		177.4	
27	21.4	1.64, s	21.4	1.63, s
28	25.4	1.32, s	25.3	1.32, s
1-OAc	171.3		171.3	
	20.6	2.10, s	20.6	2.10, s
11-OAc	172.2		172.2	
	21.4	1.91, s	21.6	1.91, s
12-OAc	171.4		171.4	
	20.9	2.08, s	20.9	2.08, s

Supplementary Table 2. ^{1}H (J) (500 MHz) NMR data of **21** and **4** (Methanol-d_4)

No.	**21**	**4**
1	4.61, d (5.6)	4.60, d (5.6)
2	3.29, m	3.29, m
3	3.21, m	3.20, m
4	1.99, m	2.00, m
	1.89, m	1.86, m
5	1.96, m	1.94, m
6	4.33, br t (4.1)	4.32, br t (4.0)
7	3.65, dd (10.3, 4.1)	3.66, dd (10.5, 4.0)
8	2.21, m	2.17, m
9	2.25, t (11.1)	2.24, t (11.1)
11	5.28, dd (11.1, 2.8)	5.24, br d (11.1)
12	5.22, d (2.8)	5.14, br s
14	1.96, m	1.94, m
15	4.39, dd (10.4, 8.4)	4.29, br t (8.4)
16	2.47, dd (13.4, 10.4)	2.07, m
17	1.82, dd (13.4, 9.8)	2.07, m
18	1.04, s	0.86, s
19	0.89, s	0.92, s
20	0.89, d (7.1)	0.99, d (7.2)
21	2.20, m	1.55, m
22	5.00, d (1.6)	3.33, d (1.3)
27	1.62, s	1.73, s
28	1.24, s	1.22, s
30	2.79, m	2.78, m
	2.67, m	2.66, m
31	3.74, m	3.73, m
34	8.47, d (1.6)	8.47, s
39	8.23, dd (8.1, 1.6)	8.23, d (8.1)
40	7.39, d (8.1)	7.39, d (8.1)
42,48	6.72, d (4.9)	6.72, d (4.9)
	6.70, d (4.9)	6.70, d (4.9)
45,51	7.32, s	7.33 s
	7.31, s	7.32, s
1-OAc	2.09, s	2.09, s
11-OAc	1.89, s	1.88, s
12-OAc	2.07, s	2.09, s
44,50-OAc	2.32, s	2.32, s

Supplementary Table 3. ^{1}H (J) (500 MHz) NMR data of **23** and **5** (Methanol-d_4)

No.	**23**	**5**
1	4.57, d (5.6)	4.56, d (5.6)
2	3.26, dd (5.6, 3.8)	3.25, dd (5.6, 3.8)
3	3.14, m	3.13, m
4	1.99, m	2.00, m
	1.87, m	1.86, m
5	1.95, m	1.95, m
6	4.33, br t (4.6)	4.32, br t (4.0)
7	3.65, dd (10.2, 4.6)	3.66, dd (10.5, 4.0)
8	2.21, m	2.17, m
9	2.25, t (11.2)	2.23, t (11.5)
11	5.29, dd (11.2, 2.9)	5.24, dd (11.5, 2.8)
12	5.22, d (2.9)	5.13, d (2.8)
14	1.96, m	1.95, m
15	4.39, dd (10.4, 8.4)	4.29, br t (8.4)
16	2.48, dd (13.5, 10.4)	2.07, m
17	1.82, dd (13.4, 9.8)	2.09, m
18	1.05, s	0.82, s
19	0.84, s	0.92, s
20	0.89, d (7.1)	0.99, d (7.4)
21	2.20, m	1.55, m
22	5.01, d (1.6)	3.33, d (1.3)
27	1.62, s	1.73, s
28	1.24, s	1.22, s
30	2.82, m	2.81, m
	2.65, m	2.64, m
31	3.74, m	3.74, m
34	8.48, d (1.6)	8.47, d (1.6)
39	8.23, dd (8.1, 1.6)	8.23, dd (8.1,1.6)
40	7.36, d (8.1)	7.36, d (8.1)
42,48	6.85, d (8.7)	6.85, d (8.7)
	6.83, d (8.7)	6.83, d (8.7)
43,49	6.89, dd (8.7, 2.3)	6.89, dd (8.7, 2.3)
	6.91, dd (8.7, 2.3)	6.91, dd (8.7, 2.3)
45,51	7.20, d (2.3)	7.21, d (2.3)
		7.22, d (2.4)
1-OAc	2.09, s	2.09, s
11-OAc	1.89, s	1.88, s
12-OAc	2.07, s	2.09, s
44,50-OAc	2.28, s	2.28, s

Supplementary Table 4. ^{1}H (J) (500 MHz) NMR data of **24** and **6** (Methanol-d_4)

No.	**24**	**6**
1	4.59, d (5.6)	4.58, d (5.6)
2	3.28, m	3.28, m
3	3.17, m	3.17, m
4	1.99, m	1.99, m
	1.88, m	1.87, m
5	1.96, m	1.96, m
6	4.34, br t (4.6)	4.33, br t (4.6)
7	3.66, dd (10.2, 4.6)	3.66, dd (10.5, 4.6)
8	2.21, m	2.17, m
9	2.26, t (11.2)	2.25, t (11.2)
11	5.31, dd (11.2, 2.9)	5.26, dd (11.2, 2.8)
12	5.22, d (2.9)	5.14, d (2.8)
14	1.97, m	1.95, m
15	4.39, dd (10.5, 8.4)	4.29, br t (8.6)
16	2.46, dd (13.5, 10.5)	2.07, m
17	1.82, dd (13.5, 9.9)	2.09, m
18	1.05, s	0.86, s
19	0.88, s	0.94, s
20	0.89, d (7.1)	1.00, d (7.4)
21	2.20, m	1.56, m
22	5.01, d (1.6)	3.34, s
27	1.62, s	1.73, s
28	1.23, s	1.22, s
30	2.82, m	2.82, m
	2.67, m	2.67, m
31	3.76, m	3.75, m
34	8.56, s	8.54, s
39	8.25, dd (8.1, 1.6)	8.24, dd (8.1,1.6)
40	7.39, d (8.1)	7.38, d (8.1)
42,48	6.83, d (9.0)	6.79, m
	6.80, d (9.0)	
43,49	6.73, d (9.0)	6.72, m
45,51	6.88, s	6.87, br s
1-OAc	2.09, s	2.10, s
11-OAc	1.89, s	1.89, s
12-OAc	2.07, s	2.10, s

Supplementary Table 5. ^{1}H (J) (500 MHz) NMR data of **7** and **8** (Methanol-d_4)

No.	**7**	**8**
1	4.56, d (5.6)	4.54, d (5.6)
2	3.25, dd (5.6, 3.8)	3.24, dd (5.6, 3.8)
3	3.13, m	3.12, m
4	1.98, m	1.98, m
	1.87, m	1.87, m
5	1.96, m	1.96, m
6	4.32, br t (4.6)	4.32, br t (4.6)
7	3.65, dd (10.2, 4.6)	3.65, dd (10.5, 4.6)
8	2.20, m	2.15, m
9	2.24, t (11.2)	2.23, t (11.2)
11	5.28, dd (11.2, 2.9)	5.24, dd (11.2, 2.8)
12	5.21, d (2.9)	5.13, d (2.8)
14	1.95, m	1.95, m
15	4.38, dd (10.5, 8.4)	4.28, br t (8.8)
16	2.46, dd (13.5, 10.5)	2.07, m
17	1.81, dd (13.5, 9.7)	2.09, m
18	1.04, s	0.81, s
19	0.82, s	0.92, s
20	0.87, d (7.1)	0.99, d (7.4)
21	2.20, m	1.54, m
22	5.00, d (1.6)	3.27, s
27	1.62, s	1.73, s
28	1.24, s	1.22, s
30	2.82, m	2.82, m
	2.64, m	2.63, m
31	3.74, m	3.74, m
34	8.49, d, (1.6)	8.49, d (1.6)
39	8.25, dd (8.1, 1.6)	8.24, dd (8.1,1.6)
40	7.38, d (8.1)	7.38, d (8.1)
42,48	6.86, d (8.7)	6.86, d (8.7)
	6.83, d (8.7)	6.82, d (8.7)
43,49	6.86, dd (8.7, 2.2)	6.86, dd (8.7, 2.2)
45,51	7.16, br s	7.16, d (2.2)
1-OAc	2.08, s	2.09, s
11-OAc	1.88, s	1.87, s
12-OAc	2.07, s	2.08, s
44,50-OPiv	1.35, s	1.35, s

Supplementary Table 6. ^{1}H (J) (500 MHz) NMR data of **27** and **9** (Methanol-d_4)

No.	**27**	**9**
1	4.65, d (5.6)	4.64, d (5.6)
2	3.37, m	3.38, dd (5.6, 3.8)
3	3.30, m	3.30, m
4	2.00, m	1.98, m
		1.87, m
5	1.98, m	2.01, m
6	4.31, br s	4.31, br s
7	3.67, dd (10.6, 4.3)	3.69, dd (10.6, 4.3)
8	2.06, m	2.05, m
9	2.28, t (11.2)	2.28, t (11.2)
11	5.28, d (11.2)	5.25, dd (11.2, 2.8)
12	5.23, s	5.15, d (2.8)
14	1.98, m	1.97, m
15	4.36, br t (9.3)	4.27, br t (8.6)
16	2.47, br t (12.1)	2.08, m
17	1.82, dd (13.5, 10.2)	2.09, m
18	1.01, s	0.89, s
19	0.98, s	0.96, s
20	0.89, d (7.0)	1.00, d (7.3)
21	2.21, m	1.55, m
22	5.01, s	3.28, s
27	1.62, s	1.74, s
28	1.24, s	1.24, s
30	4.25, d (16.0)	4.23, d (16.0)
	4.11, d (16.0)	4.12, d (16.0)
33	8.54, s	8.54, d (1.6)
38	8.25, d (8.0)	8.27, dd (8.1,1.6)
39	7.38, d (8.0)	7.38, d (8.1)
41,47	6.90, d (8.7)	6.89, m
42,48	6.87, d (8.7)	6.89, m
44,50	7.16, br s	7.16, d (2.2)
1-OAc	2.10, s	2.10, s
11-OAc	1.90, s	1.90, s
12-OAc	2.07, s	2.10, s
43,49-OPiv	1.35, s	1.35, s

Supplementary Table 7. ^{1}H (J) (500 MHz) NMR data of **10** (Acetonitrile-d_3) and **11** (Methanol-d_4)

No.	**10**	**11**
1	4.69, d (5.6)	4.69, d (5.6)
2	3.33, dd (5.6, 3.8)	3.40, dd (5.6, 3.8)
3	3.26, m	3.34, m
4	2.03, m	2.11, m
5	2.06, m	2.12, m
6	4.60, br s	4.60, br s
7	3.78, d (10.0)	3.78, dd (10.0, 4.8)
8	2.04, m	2.33, m
9	2.37, t (10.9)	2.32, t (11.3)
11	5.25, dd (10.9, 2.8)	5.30, dd (11.3, 2.9)
12	5.16, d (2.8)	5.16, d (2.9)
14	2.04, m	1.97, t (8.9)
15	4.33, dd (10.4, 8.4)	4.28, t (8.9)
16	2.47, dd (13.5, 10.4)	2.06, m
17	1.82, dd (13.5, 10.8)	2.08, m
18	1.01, s	0.91, s
19	1.05, s	1.08, s
20	0.86, d (7.0)	1.00, d (7.3)
21	2.18, m	1.55, m
22	4.99, d (1.7)	3.28, s
27	1.61, s	1.74, s
28	1.25, s	1.24, s
31	8.40, s	8.36, d (1.6)
36	8.14, d (8.0)	8.11, dd (8.1,1.6)
37	7.37, d (8.0)	7.36, d (8.1)
39,45	6.92, d (8.7)	6.90, d (8.7)
		6.88, d (8.7)
40,46	6.86, dd (8.7, 2.2)	6.85, dd (8.7, 2.2)
42,48	7.14, d (2.2)	7.17, d (2.2)
1-OAc	2.07, s	2.12, s
11-OAc	1.87, s	1.91, s
12-OAc	2.04, s	2.12, s
41,47-OPiv	1.34, s	1.35, s

Supplementary Table 8. ^{1}H (J) (500 MHz) NMR data of **28** (Methanol-d_4) and **12** (Methanol-d_4)

No.	**28**	**12**
1	4.71, d (5.6)	4.69, d (5.6)
2	3.41, dd (5.6, 3.8)	3.41, dd (5.6, 3.8)
3	3.34, m	3.35, m
4	2.13, m	2.13, m
5	2.12, m	2.12, m
6	4.62, br s	4.62, br s
7	3.78, dd (10.2, 4.7)	3.79, dd (10.1, 4.7)
8	2.38, m	2.35, m
9	2.33, t (11.3)	2.33, t (11.2)
11	5.36, dd (11.3, 2.8)	5.32, dd (11.2, 2.9)
12	5.24, d (2.8)	5.16, d (2.9)
14	2.00, dd (10.1, 8.1)	1.98, t (8.9)
15	4.39, dd (10.4, 8.1)	4.29, t (8.9)
16	2.46, dd (13.5, 10.4)	2.06, m
17	1.84, dd (13.5, 9.7)	2.08, m
18	1.04, s	0.93, s
19	1.12, s	1.10, s
20	0.90, d (7.1)	1.00, d (7.4)
21	2.20, m	1.55, m
22	5.01, d (1.5)	3.34, s
27	1.64, s	1.75, s
28	1.28, s	1.25, s
31	8.40, s	8.38, s
36	8.12, d (8.0)	8.10, dd (8.0,1.6)
37	7.35, d (8.0)	7.34, d (8.0)
39,45	6.76, d (8.7)	6.73, d (8.7)
	6.75, d (8.7)	6.71, d (8.7)
40,46	6.66, d (8.7)	6.63, d (8.7)
	6.64, d (8.7)	6.61, d (8.7)
42,48	6.82, s	6.79, s
1-OAc	2.12, s	2.13, s
11-OAc	1.92, s	1.91, s
12-OAc	2.09, s	2.12, s

Supplementary Table 9. Primer list for site-directed mutagenesis.

Primer Name	Primer sequence (5'->3')	Tm (°C)	Nucleotide #
K19A For	caaccagatcggtgccgcgttctgggaggtgatc	78.68	34
K19A Rev	gatcacctcccagaacgcggcaccgatctggttg	78.68	34
L217A For	gatatctgcttccgcactgcgaagctgaccacaccaac	80.05	38
L217A Rev	gttggtgtggtcagcttcgcagtgcggaagcagatatc	80.05	38
L219A For	cttccgcactctgaaggcgaccacaccaacctac	78.68	34
L219A Rev	gtaggttggtgtggtcgccttcagagtgcggaag	78.68	34
D226A For	caccaacctacggggctctgaaccaccttgt	78.98	31
D226A Rev	acaaggtggttcagagccccgtaggttggtg	78.98	31
D226N For	cacaccaacctacgggaatctgaaccaccttgt	79.14	33
D226N Rev	acaaggtggttcagattcccgtaggttggtgtg	79.14	33
T223A For	gaagctgaccacaccagcctacggggatct	78.9	30
T223A Rev	agatccccgtaggctggtgtggtcagcttc	78.9	30
H229A For	ctacggggatctgaacgcccttgtctcagccacc	79.88	34
H229A Rev	ggtggctgagacaagggcgttcagatccccgtag	79.88	34
R278A For	ccctctcaccagcgctggaagccagcag	78.07	28
R278A Rev	ctgctggcttccagcgctggtgagaggg	78.07	28

Supplementary Table 10. Multiply adjusted P values for the binding of **11** to ectopically expressed mutant tubulin (Fig. 7b, d) or endogenously expressed tubulin in the mutant expressing lines (Fig. 7c, e). Statistical analysis was performed using a one-way ANOVA (20 degrees of freedom) with Tukey's posthoc test. n = 3 for all conditions other than WT and D226A controls, where n = 5.

Comparison	Adjusted P values for binding to ectopically expressed tubulin	Adjusted P values for binding to endogenous tubulin
WT vs. K19A	< 0.0001	> 0.9999
WT vs. L217A	0.2353	0.9986
WT vs. L219A	0.0002	0.9998
WT vs. T223A	0.0169	> 0.9999
WT vs. D226A	< 0.0001	0.9155
WT vs. H229A	0.0043	> 0.9999
WT vs. R278A	0.9961	> 0.9999
K19A vs. L217A	0.0264	0.9992
K19A vs. L219A	0.9976	0.9999
K19A vs. T223A	0.2622	> 0.9999
K19A vs. D226A	> 0.9999	0.9548
K19A vs. H229A	0.5605	> 0.9999
K19A vs. R278A	< 0.0001	> 0.9999
L217A vs. L219A	0.0973	> 0.9999
L217A vs. T223A	0.9209	> 0.9999
L217A vs. D226A	0.0102	0.9997
L217A vs. H229A	0.6462	0.9999
L217A vs. R278A	0.1267	0.9993
L219A vs. T223A	0.6095	> 0.9999
L219A vs. D226A	0.995	0.9982
L219A vs. H229A	0.9009	> 0.9999
L219A vs. R278A	0.0002	> 0.9999
T223A vs. D226A	0.1579	0.9938
T223A vs. H229A	0.999	> 0.9999
T223A vs. R278A	0.0107	> 0.9999
D226A vs. H229A	0.4228	0.98
D226A vs. R278A	< 0.0001	0.9583
H229A vs. R278A	0.0031	> 0.9999

Supplementary Table 11. Key atom distances in the lowest-energy model of β-tubulin docked to the taccalonolide probe **12**.

Residue	Residue atom	Residue atom to probe C22 (Å)	Probe atom	Probe atom to probe C22 (Å)
D226	O6840 (-δO-C22-)[a]	1.4	-	-
L219	H3390 (-δCH_3)[b]	4.5	C21[e]	2.5
K19	H395 (-ζNH_3^+)[a]	6.4	C25-OH[d]	4.8
H229	N3539 (-δN=)[a]	7.0	C15-OH[d]	5.2
	H6836 (-εNH-)[a]	8.7	C1-O-Ac[d]	8.7
L217	H3347 (-γCH_2-)[b]	8.3	C12-O-(C=O)-CH_3[e]	5.2
R278	H4313 (-εNH-)[a]	9.8	C11-O-(C=O)-CH_3[d]	8.8
T223	H3450 (-γOH)[c]	4.6	C26=O[d]	4.4

[a]Residue atoms (red) directly connect to (covalent) or interact (via H-bonds) with probe.
[b]Residue atoms (red) that interact with probe via hydrophobic interactions have shortest distance to C22.
[c]Residue atoms (red) indirectly interact (via H_2O bridge) with probe.
[d]Probe atoms (red) directly (via H-bonds or hydrophobic interactions) or indirectly (via H_2O bridge) interact with receptor.
[e]Probe atoms (red) that interact with a receptor residue via hydrophobic interactions have shortest distance to C22

Supplementary Methods

General methods

NMR data were obtained on Varian VNMR spectrometers (400 and 500 MHz for ^{1}H, 100 and 125 MHz for ^{13}C) with broad band and triple resonance probes. Preparative HPLC separations were performed on a Shimadzu system using a SCL-10A VP controller and a Gemini 5 μm C18 or a Kinetex F5 5 μm C18 column (110 Å, 250 × 21.2 mm) with flow rate of 10 mL/min. Semi-preparative HPLC separations were performed on a Waters 1525 system using a 2998 PDA detector and Gemini 5 μm C18 or a Kinetex F5 5 μm C18 column (110 Å, 250 × 10.0 mm) with flow rate of 4 mL/min. All solvents were of ACS grade or better. HRESIMS (high-resolution electrospray ionization mass spectrometry) data were collected on an Agilent 6538 high-mass-resolution QTOF mass spectrometer. X-ray Intensity data were collected using a diffractometer with a Bruker APEX ccd area detector and graphite-monochromated Mo Kα radiation (λ = 0.71073 Å).

Compound characterization and purity

Both 1D (^{1}H and ^{13}C) and 2D (^{1}H-^{1}H COSY, HSQC, and HMBC) NMR data were collected for key new compounds to confirm their structures and assign the ^{1}H NMR data (Supplementary Tables 1-8). In case of new compounds with sensitive structural features (i.e. 22,23-epoxide) and/or low quantities (< 2 mg), time-consuming ^{13}C NMR data were not collected to avoid potential structural decompositions under the NMR conditions. Instead, besides ^{1}H NMR data, the

fast 2D NMR data (^{1}H-^{1}H COSY and HSQC) were also collected to confirm their structures and unambiguously assign their ^{1}H NMR data (Supplementary Tables 2-8). 1D and 2D NMR spectra for all synthetic compounds were attached as references and evidence of compound purity (95% or better). HRESIMS data were collected and listed for key new compounds to confirm their identity.

Reductive amination of taccalonolide B (13)

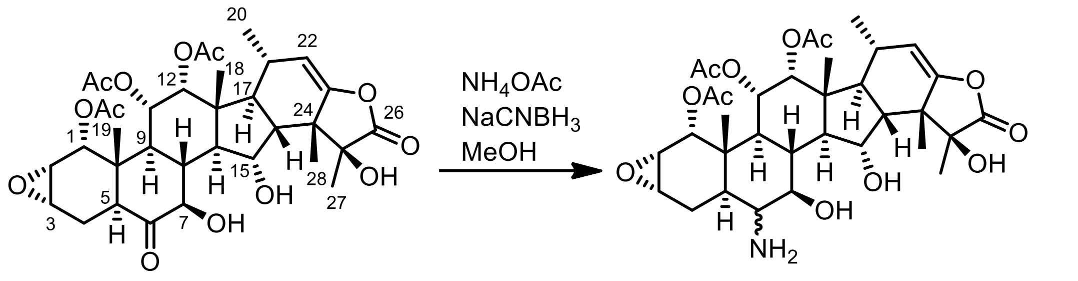

Taccalonolide B (**13**)

6-NH_2-taccalonolide B (**14**) (6*S* : 6*R* = 3 : 1)

Supplementary Figure 82. Synthesis of 6-NH_2-taccalonolide B (**14**)

Taccalonolide B (**13**, 50 mg, 1 equiv) was mixed with ammonium acetate (165 mg, 30 equiv), sodium cyanoborohydride (45 mg, 10 equiv), and 4 Å molecular sieves (500 mg) in anhydrous methanol (MeOH) (4 mL). The reactant mixture was stirred at 35°C overnight and the solvent was removed in vacuo. The residue was purified by preparative HPLC using a Luna 5 µm C18 column [isocratic, 40% acetonitrile (MeCN) in 0.1% trifluoroacetic acid (TFA)] to yield the TFA salt **TFA-14** (45 mg) which was identified as a 3 : 1 mixture of the 6*S* and 6*R* epimers by analysis of 1D and 2D NMR data. The TFA salt was then stirred with the AMBERLYST™ A21 resin (450 mg) in methanol (4 mL) for 2 h at room

temperature. The resin was filtered and washed with methanol (4 mL x 2). The combined methanol filtrates were evaporated in vacuo to yield the free amine 6-NH_2-taccalonolide B (**14**) (33 mg).

6-NH_2-taccalonolide B (**14**): colorless solid; ^1H and ^{13}C NMR data of **TFA-14**, see Supplementary Table 1; HRESIMS (m/z): $[M+H]^+$ calcd. for $C_{34}H_{48}NO_{12}$, 662.3177; found 662.3177.

Synthesis of 5-carboxyfluorescein dipivalate (16)

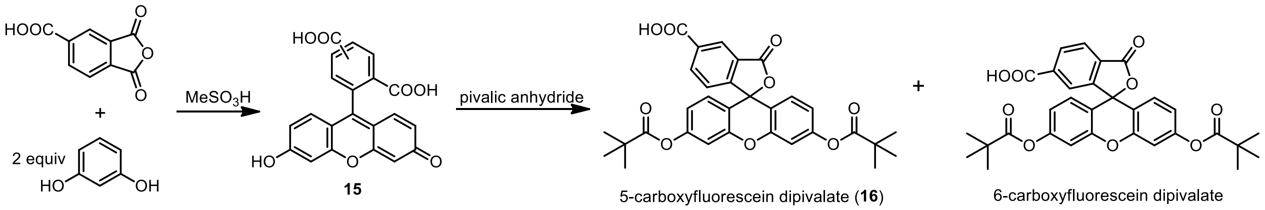

Supplementary Figure 83. Synthesis of 5-carboxyfluorescein dipivalate (**16**)

Trimellitic anhydride (436 mg, 1 equiv) and resorcinol (500 mg, 2 equiv) were stirred in methanesulfonic acid (12 mL) at 80°C for 24 h. The resulted mixture was poured into ice water (H_2O). The precipitate was collected and washed with water to yield the crude 5(6)-carboxyfluorescein (**15**) (820 mg).

5(6)-carboxyfluorescein (**15**)[1]: yellow powder; ^1H NMR (500 MHz, methanol-d_4): δ 8.59 (d, J = 1.5 Hz, 1H), 8.37 (dd, J = 8.0, 1.5 Hz, 1H), 8.31 (dd, J = 8.0, 1.5 Hz, 1H), 8.09 (d, J = 8.0 Hz, 1H), 7.74 (s, 1H), 7.30 (d, J = 8.0 Hz, 1H), 6.70-6.72 (d, J = 9.7 Hz, 4H), 6.60-6.42 (br s, 4H), 6.53-6.57 (m, 4H).

5(6)-carboxyfluorescein (**15**) (490 mg) was stirred in trimethylacetic anhydride (8 mL, 30 equiv) at 110°C for 3 h. The resulted mixture was then stirred in the mixed solution of water (15 mL) and THF (30 mL) at room temperature for 2 days. The solvents were removed in vacuo and the residue was purified by preparative HPLC using a Luna 5 µm C18 column (isocratic, 80% MeCN in 0.1% TFA) to yield 5-carboxyfluorescein dipivalate (**16**) (221 mg) and 6-carboxyfluorescein dipivalate (198 mg).

5-carboxyfluorescein dipivalate (**16**)[2]: yellow powder; ^{1}H NMR (400 MHz, methanol-d_4): δ 8.56 (s, 1H), 8.23 (d, J = 8.0 Hz, 1H), 7.12 (dd, J = 8.0, 1.6 Hz, 1H), 7.05 (br s, 2H), 6.75-6.80 (m, 4H), 1.28 (s, 18H); ^{13}C NMR (100 MHz, methanol-d_4): δ 177.7, 169.6, 167.6, 157.6, 154.1, 152.6, 137.6, 134.4, 130.0, 127.6, 127.5, 125.3, 119.1, 116.8, 111.4, 83.1, 40.0, 27.4.

Synthesis of 5-carboxyfluorescein diacetate (17)

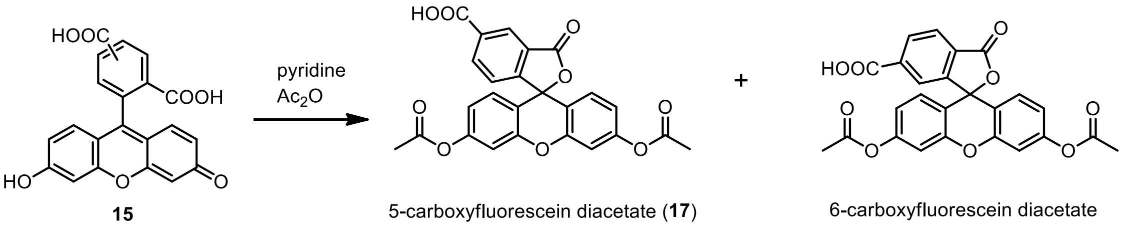

Supplementary Figure 84. Synthesis of 5-carboxyfluorescein diacetate (**17**)

5(6)-carboxyfluorescein (**15**) (320 mg) and pyridine (260 µL) were stirred in acetic anhydride (5 mL) at 80°C for 5 min. The solvents were removed in vacuo and the residue was purified by preparative HPLC using a Luna 5 µm C18 column

(isocratic, 50% MeCN in 0.1% formic acid) to yield 5-carboxyfluorescein diacetate (**17**) (161 mg) and 6-carboxyfluorescein diacetate (152 mg).

5-carboxyfluorescein diacetate (**17**): yellow powder; ^{1}H NMR (400 MHz, methanol-*d*4): δ 8.61 (s, 1H), 8.37 (d, J = 8.0 Hz, 1H), 7.32 (d, J = 8.0 Hz, 1H), 7.18 (br s, 2H), 6.88 (br s, 4H), 2.28 (s, 6H).

Synthesis of 5(6)-carboxy-Oregon Green 488 (19)

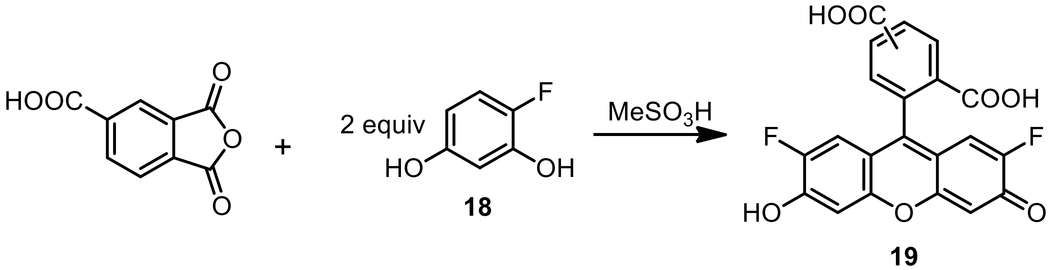

Supplementary Figure 85. Synthesis of 5(6)-carboxy-Oregon Green 488 (**19**)

Trimellitic anhydride (375 mg, 1 equiv) and 4-fluororesorcinol (**18**) (500 mg, 2 equiv) were stirred in methanesulfonic acid (12 mL) at 80°C for 24 h. The resulted mixture was poured into ice water. The precipitate was collected and washed with water to yield the crude 5(6)-carboxy-Oregon Green 488 (**19**) (930 mg).

5(6)-carboxy-Oregon Green 488 (**19**)[3]: yellow powder; ^{1}H NMR (500 MHz, methanol-*d*4): δ 8.61 (d, J = 1.5 Hz, 1H), 8.31 (dd, J = 8.0, 1.5 Hz, 1H), 8.25 (dd, J = 8.0, 1.5 Hz, 1H), 8.11 (d, J = 8.0 Hz, 1H), 7.81 (s, 1H), 7.30 (d, J = 8.0 Hz, 1H), 6.83 (s, 1H), 6.81 (s, 2H), 6.80 (s, 1H), 6.51 (s, 1H), 6.50 (s, 1H), 6.49 (s, 1H), 6.48 (s, 1H).

Synthesis of diacetyl-5-(2-carboxyethylaminocarbonyl) Oregon Green 488 (20)[4]

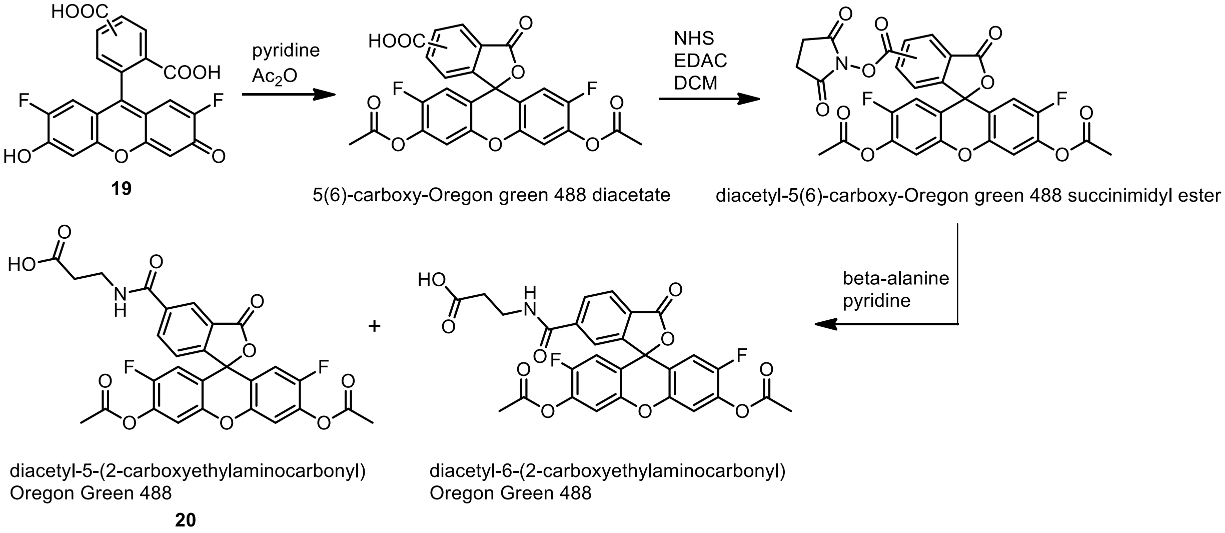

Supplementary Figure 86. Synthesis of diacetyl-5-(2 carboxyethylaminocarbonyl) Oregon Green 488 (**20**)

5(6)-carboxy-Oregon green (**19**) (167 mg) and pyridine (130 uL) were stirred in acetic anhydride (3.8 mL) at 80°C for 5 min. The solvents were removed in vacuo to yield the crude 5(6)-carboxy-Oregon green diacetate (198 mg). A portion of the crude 5(6)-carboxy-Oregon green diacetate (30 mg) was stirred with *N*-hydroxysuccinimide (NHS) (8.3 mg, 1.2 equiv) and 1-Ethyl-3-(3-dimethylaminopropyl)carbodiimide (EDAC) (14 mg, 1.2 equiv) in dichloromethane (DCM) (2 mL) at room temperature for 3 h and then beta-alanine (54 mg, 10 equiv) and pyridine (2 mL) were added into the mixture which was further stirred at 40°C overnight. The solvents were removed in vacuo and the residue was

purified by preparative HPLC using a Luna 5 µm C18 column (isocratic, 35% MeCN in 0.1% TFA) to yield diacetyl-5-(2-carboxyethylaminocarbonyl) Oregon Green 488 (**20**) (15.2 mg) and diacetyl-6-(2-carboxyethylaminocarbonyl) Oregon Green 488 (13.4 mg).

diacetyl-5-(2-carboxyethylaminocarbonyl) Oregon Green 488 (**20**): yellow powder; ^{1}H NMR (500 MHz, methanol-d_4): δ 8.44 (d, J = 1.6 Hz, 1H), 8.20 (dd, J = 8.0, 1.6 Hz, 1H), 7.39 (d, J = 8.0 Hz, 1H), 7.32 (s, 1H), 7.31 (s, 1H), 6.76 (s, 1H), 6.74 (s, 1H), 3.68 (t, J = 6.8 Hz, 1H), 2.68 (t, J = 6.8 Hz, 1H), 2.32 (s, 6H).

Synthesis of Flu-tacca-2 (4)

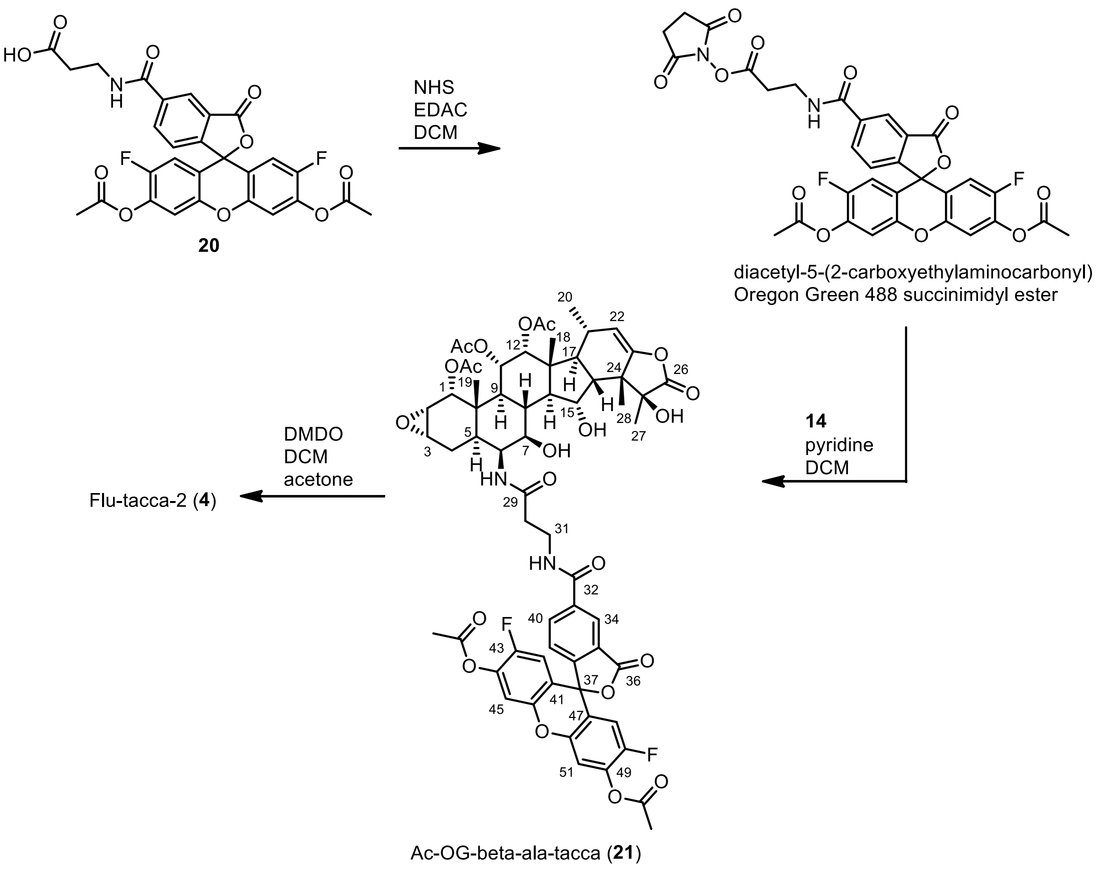

Supplementary Figure 87. Synthesis of Flu-tacca-2 (**4**)

Compound **20** (24 mg) was stirred with NHS (5.6 mg, 1.2 equiv) and EDAC (7.8 mg, 1.2 equiv) in DCM (2 mL) at room temperature for 4 h followed by the removal of the solvent in vacuo. The residue was resuspended in saturated brine (2 mL) and partitioned with ethyl acetate (EtOAc) (4 mL x 3). The combined organic layers were dried down in vacuo to yield the crude diacetyl-5-(2-carboxyethylaminocarbonyl) Oregon Green 488 succinimidyl ester which was directly mixed with **14** (11 mg) in a DCM (2 mL)/pyridine (2 mL) solution. The reactant mixture was further stirred at 40°C for 24 h. The solvents were removed in vacuo and the residue was purified by semi-preparative HPLC using a Kinetex 5 µm F5 column (isocratic, 50% MeCN in 0.1% formic acid) to yield compound **21** (2.6 mg).

Ac-OG-beta-ala-tacca (**21**): light yellow solid; ^{1}H NMR data, see Supplementary Table 2; ^{13}C NMR (125 MHz, methanol-d_4) (some carbons were not detectable due to limited amount of material): δ 177.4, 175.3, 172.3, 171.6, 171.5, 169.4, 169.3, 168.1, 155.9, 153.1, 151.2, 148.4, 141.8, 138.5, 136.0, 127.5, 125.5, 125.4, 117.3, 117.2, 115.5, 115.4, 114.2, 83.9, 80.3, 75.6, 75.3, 73.6, 72.6, 72.5, 58.1, 54.1, 54.0, 51.9, 51.4, 49.2, 45.7, 42.1, 38.3, 38.1, 36.8, 36.4, 34.4, 32.2, 27.1, 25.4, 22.1, 21.5, 21.0, 20.7, 20.6, 20.2, 13.7, 13.5; HRESIMS (m/z): $[M+Na]^+$ calcd. for $C_{62}H_{64}F_2N_2NaO_{21}$, 1233.3862; found 1233.3859.

Compound **21** (2.6 mg) was dissolved in 0.3 mL DCM and prechilled to -20°C prior to the addition of 0.3 mL DMDO-acetone solution. The epoxidation reagent dimethyldioxirane (DMDO) was prepared as previous described[5]. The mixture

was incubated at -20 °C for 4 h and then blown down by N_2**.** The residue was purified by semi-preparative HPLC using a Kinetex 5 µm F5 column (isocratic, 50% MeCN) to yield compound **4** (1.6 mg).

Flu-tacca-2 **(4)**: light yellow solid; ^{1}H NMR data, see Supplementary Table 2; HRESIMS (m/z): $[M+Na]^+$ calcd. for $C_{62}H_{64}F_2N_2NaO_{22}$, 1249.3811; found 1249.3814.

Synthesis of diacetyl-5-(2-carboxyethylaminocarbonyl) fluorescein (22)[4]

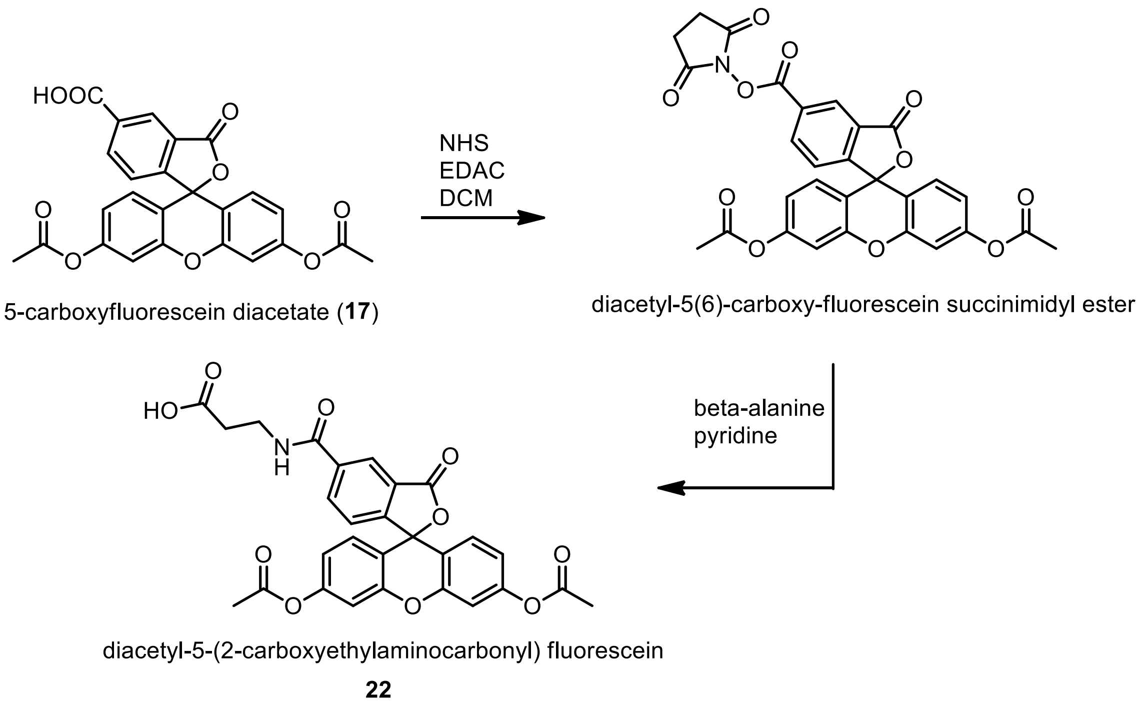

Supplementary Figure 88. Synthesis of diacetyl-5-(2-carboxyethylaminocarbonyl) fluorescein (**22**)

5-Carboxyfluorescein diacetate (**17**) (28 mg) was stirred with NHS (8.3 mg, 1.2 equiv) and EDAC (14 mg, 1.2 equiv) in DCM (2 mL) at room temperature for 3 h

and then beta-alanine (54 mg, 10 equiv) and pyridine (2 mL) were added into the mixture which was further stirred at 40°C overnight. The solvents were removed in vacuo and the residue was purified by preparative HPLC using a Luna 5 µm C18 column (isocratic, 50% MeCN in 0.1% formic acid) to yield diacetyl-5-(2-carboxyethylaminocarbonyl) fluorescein (**22**) (20 mg).

Diacetyl-5-(2-carboxyethylaminocarbonyl) fluorescein (**22**): light yellow powder; ^{1}H NMR (400 MHz, methanol-d_4): δ 8.43 (d, J = 1.6 Hz, 1H), 8.17 (dd, J = 8.0, 1.6 Hz, 1H), 7.30 (d, J = 8.0 Hz, 1H), 7.16 (s, 2H), 6.86 (m, 4H), 3.67 (t, J = 6.8 Hz, 1H), 2.67 (t, J = 6.8 Hz, 1H), 2.27 (s, 6H); ^{13}C NMR (100 MHz, methanol-d_4): δ 170.5, 170.0, 168.2, 156.6, 154.0, 152.8, 138.2, 135.9, 130.0, 127.7, 125.5, 125.1, 119.4, 117.1, 111.6, 83.3, 37.3, 20.9.

Synthesis of Flu-tacca-3 (5)

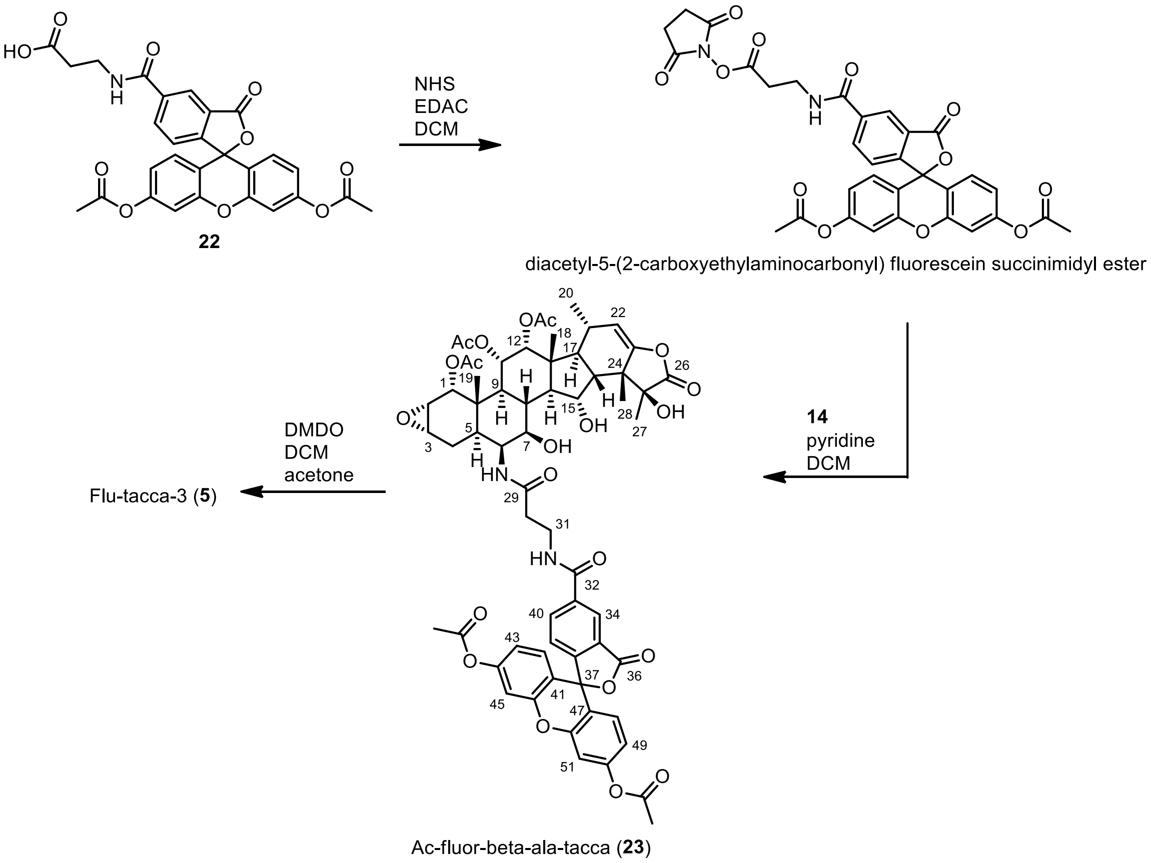

Supplementary Figure 89. Synthesis of Flu-tacca-3 (**5**)

Compound **22** (20 mg) was stirred with NHS (5.2 mg, 1.2 equiv) and EDAC (8.7 mg, 1.2 equiv) in DCM (2 mL) at room temperature for 4 h followed by the removal of the solvent in vacuo. The residue was resuspended in saturated brine (2 mL) and partitioned with EtOAc (4 mL x 3). The combined organic layers were dried down in vacuo to yield the crude diacetyl-5-(2-carboxyethylaminocarbonyl) fluorescein succinimidyl ester (24 mg) which was directly mixed with **14** (9 mg) in a DCM (2 mL)/pyridine (2 mL) solution. The reactant mixture was further stirred at 40°C overnight. The solvents were removed in vacuo and the residue was purified by semi-preparative HPLC using a Kinetex 5 µm F5 column (isocratic, 45% MeCN in 0.1% formic acid) to yield compound **23** (5.0 mg).

Ac-fluor-beta-ala-tacca (**23**): light yellow solid; ^{1}H NMR data, see Supplementary Table 3; ^{13}C NMR (125 MHz, methanol-d_4): δ 177.3, 175.3, 172.3, 171.6, 171.5, 170.5, 170.4, 169.9, 168.0, 156.0, 155.9, 154.1, 152.8, 138.0, 135.9, 129.9, 129.8, 127.7, 125.6, 125.1, 119.5, 119.4, 117.0, 112.1, 111.8, 111.7, 83.3, 80.3, 75.6, 75.4, 73.6, 72.6, 72.5, 58.1, 54.1, 54.0, 51.9, 51.4, 49.3, 45.7, 42.1, 38.3, 38.1, 36.8, 36.4, 34.4, 32.2, 27.1, 25.4, 22.1, 21.5, 21.0, 20.9, 20.7, 20.6, 13.7, 13.5; HRESIMS (m/z): $[M+Na]^+$ calcd. for $C_{62}H_{66}N_2NaO_{21}$, 1197.4050; found 1197.4055.

Compound **23** (5.0 mg) was epoxidized in 1 mL DMDO-acetone/DCM (1 : 1) solution as previously described. The product was purified by semi-preparative HPLC using a Kinetex 5 µm F5 column (isocratic, 45% MeCN) to yield compound **5** (4.2 mg).

Flu-tacca-3 (**5**): light yellow solid; ^{1}H NMR data, see Supplementary Table 3; HRESIMS (m/z): $[M+Na]^+$ calcd. for $C_{62}H_{66}N_2NaO_{22}$, 1213.3999; found 1213.4006.

Synthesis of Flu-tacca-4 (6)

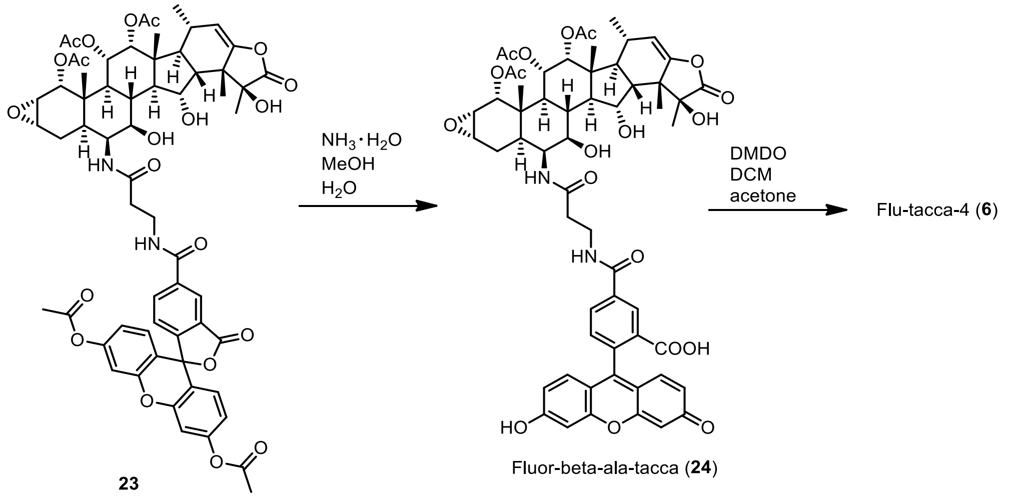

Supplementary Figure 90. Synthesis of Flu-tacca-4 (**6**)

Compound **23** (11 mg) was dissolved in 3 mL MeOH/H_2O (2 : 1) followed by the addition of 50 µL ammonia hydroxide ($NH_3 \cdot H_2O$). The mixture was stirred at room temperature for 1 h and then the solvents were removed in vacuo**.** The residue was purified by preparative HPLC using a Kinetex 5 µm F5 column (isocratic, 40% MeCN in 0.1% TFA) to yield compound **24** (9.5 mg).

Fluor-beta-ala-tacca (**24**): yellow solid; ^{1}H NMR data, see Supplementary Table 4; HRESIMS (m/z): $[M+Na]^+$ calcd. for $C_{58}H_{62}N_2NaO_{19}$, 1113.3839; found 1113.3830.

Compound **24** (6.0 mg) was epoxidized in 1 mL DMDO-acetone/DCM (1 : 1) solution as previously described. The product was purified by preparative HPLC using a Kinetex 5 µm F5 column (isocratic, 45% MeCN in 0.1% TFA) to yield compound **6** (5.2 mg).

Flu-tacca-4 (**6**): yellow solid; ^{1}H NMR data, see Supplementary Table 4; HRESIMS (m/z): $[M+Na]^+$ calcd. for $C_{58}H_{62}N_2NaO_{20}$, 1129.3788; found 1129.3799.

Synthesis of dipivalyl-5-(2-carboxyethylaminocarbonyl) fluorescein (25)[4]

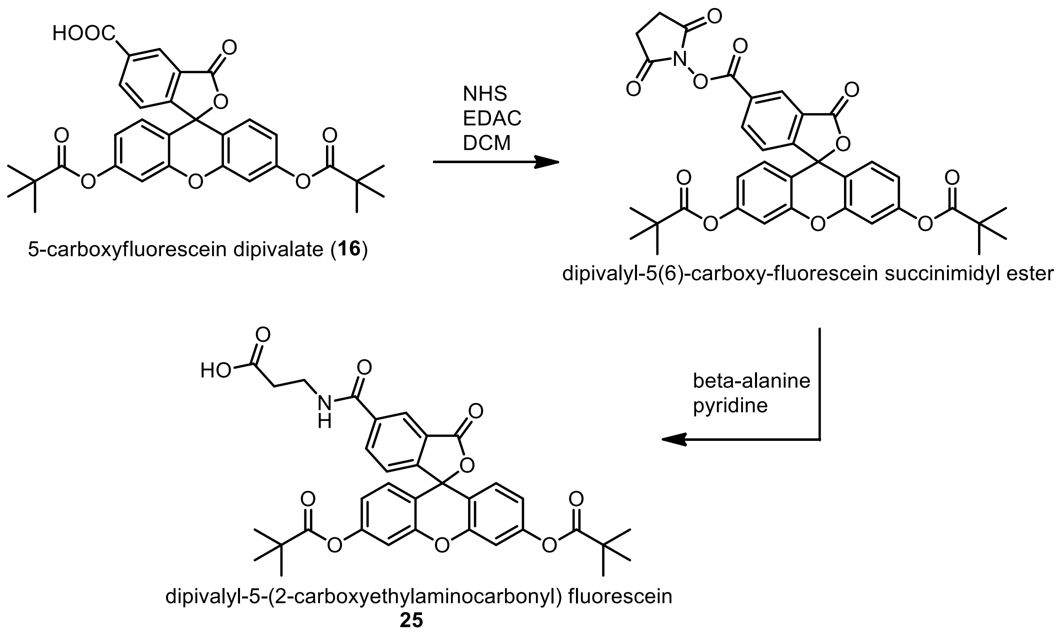

Supplementary Figure 91. Synthesis of dipivalyl-5-(2-carboxyethylaminocarbonyl) fluorescein (**25**)

5-carboxyfluorescein dipivalate (**16**) (33 mg) was stirred with NHS (8.3 mg, 1.2 equiv) and EDAC (14 mg, 1.2 equiv) in DCM (2 mL) at room temperature for 3 h and then beta-alanine (54 mg, 10 equiv) and pyridine (2 mL) were added into the mixture which was further stirred at 40°C overnight. The solvents were removed in vacuo and the residue was purified by preparative HPLC using a Luna 5 µm C18 column (isocratic, 85% MeCN in 0.1% formic acid) to yield dipivalyl-5-(2-carboxyethylaminocarbonyl) fluorescein (**25**) (27 mg).

Dipivalyl-5-(2-carboxyethylaminocarbonyl) fluorescein (**25**): white powder; ^{1}H NMR (400 MHz, methanol-d_4): δ 8.44 (d, J = 1.6 Hz, 1H), 8.17 (dd, J = 8.0, 1.6 Hz, 1H), 7.30 (d, J = 8.0 Hz, 1H), 7.13 (d, J = 2.1 Hz, 2H), 6.86 (d, J = 8.7 Hz, 2H), 6.86 (d, J = 8.7 Hz, 2H), 6.83 (dd, J = 8.7, 2.1 Hz, 2H), 3.67 (t, J = 6.8 Hz,

1H), 2.67 (t, J = 6.8 Hz, 1H), 1.34 (s, 18H); ^{13}C NMR (100 MHz, methanol-d_4): δ 177.9, 169.9, 168.1, 156.6, 154.3, 152.8, 138.1, 135.9, 130.1, 127.8, 125.5, 125.1, 119.3, 117.1, 111.5, 83.3, 40.2, 37.3, 27.4.

Synthesis of Flu-tacca-5 (8)

Compound **25** (27 mg) was stirred with NHS (6.0 mg, 1.2 equiv) and EDAC (10 mg, 1.2 equiv) in DCM (2 mL) at room temperature for 4 h followed by the removal of the solvent in vacuo. The residue was resuspended in saturated brine (2 mL) and partitioned with EtOAc (4 mL x 3). The combined organic layers were dried down in vacuo to yield the crude dipivalyl-5-(2-carboxyethylaminocarbonyl) fluorescein succinimidyl ester which was directly mixed with **14** (11 mg) in a DCM (2 mL)/pyridine (2 mL) solution. The reactant mixture was further stirred at 40°C overnight. The solvents were removed in vacuo and the residue was purified by semi-preparative HPLC using a Kinetex 5 µm F5 column (isocratic, 55% MeCN in 0.1% formic acid) to yield compound **7** (6.8 mg).

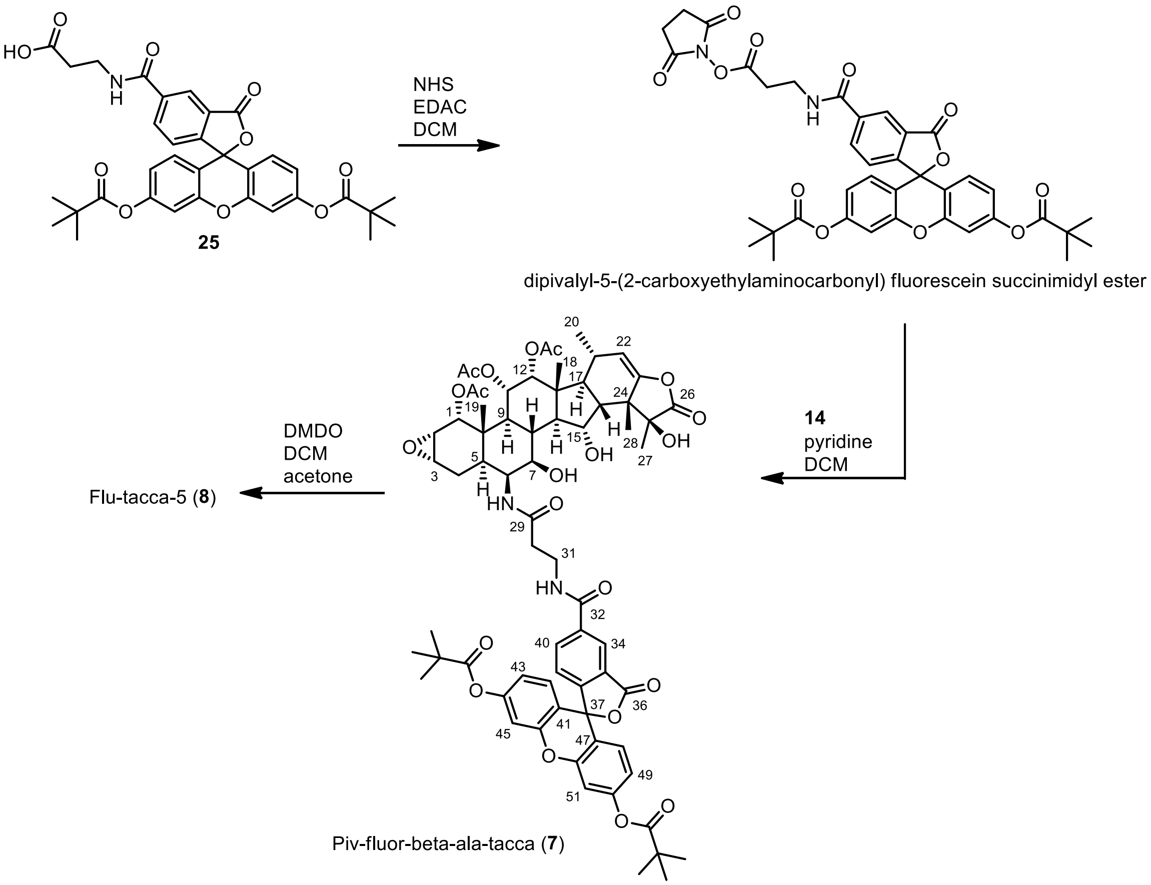

Supplementary Figure 92. Synthesis of Flu-tacca-5 (**8**)

Piv-fluor-beta-ala-tacca (**7**): white solid; ^{1}H NMR data, see Supplementary Table 5; ^{13}C NMR (125 MHz, methanol-d_4): δ 177.9 (2C), 177.3, 175.3, 172.4, 171.5, 171.4, 169.9, 167.9, 156.9, 155.9, 154.4, 152.8, 138.0, 135.9, 130.0, 129.9, 127.8, 125.7, 125.0, 119.4, 119.3, 117.0, 116.9, 112.1, 111.7, 111.6, 83.4, 80.3, 75.6, 75.4, 73.6, 72.6, 72.5, 58.1, 54.1, 54.0, 51.9, 51.3, 49.3, 45.7, 42.1, 40.2, 38.3, 38.1, 36.8, 36.4, 34.4, 32.2, 27.4, 27.1, 25.4, 22.1, 21.6, 21.0, 20.7, 20.6, 13.8, 13.6; HRESIMS (m/z): $[M+Na]^+$ calcd. for $C_{68}H_{78}N_2NaO_{21}$, 1281.4989; found 1281.5015.

Compound **7** (6.0 mg) was epoxidized in 1 mL DMDO-acetone/DCM (1 : 1) solution as previously described. The product was purified by semi-preparative HPLC using a Kinetex 5 µm F5 column (isocratic, 65% MeCN) to yield compound **8** (5.8 mg).

Flu-tacca-5 (**8**): light yellow solid; ^{1}H NMR data, see Supplementary Table 5; HRESIMS (m/z): $[M+Na]^+$ calcd. for $C_{68}H_{78}N_2NaO_{22}$, 1297.4938; found 1297.4880.

Synthesis of Fmoc-Gly-tacca (26)[6]

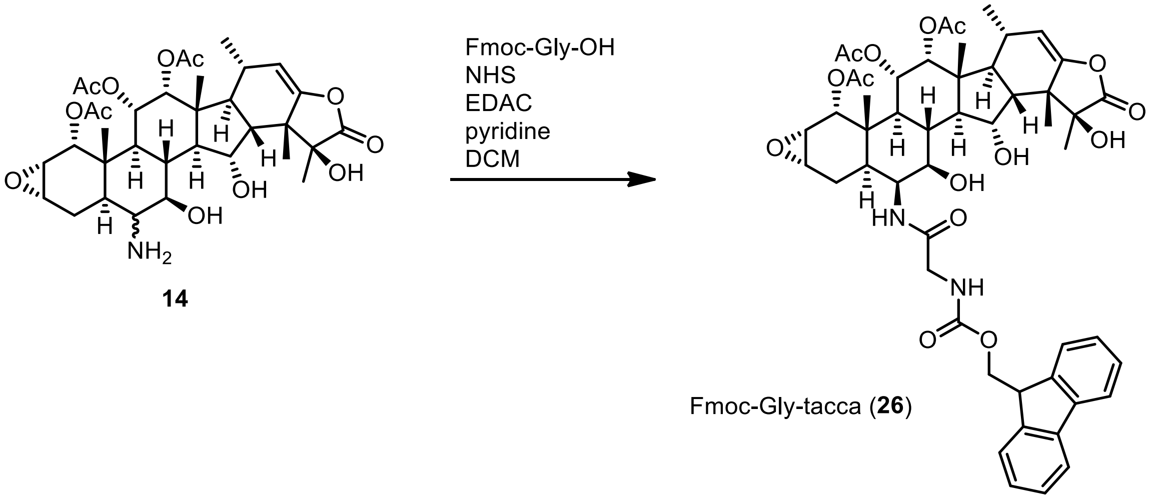

Supplementary Figure 93. Synthesis of Fmoc-Gly-tacca (**26**)

Compound **14** (10 mg) was stirred with Fmoc-Gly-OH (6.7 mg, 1.5 equiv), NHS (2.7 mg, 1.5 equiv) and EDAC (4.3 mg, 1.5 equiv) in 2 mL DCM/pyridine (1 : 1) at room temperature overnight followed by the removal of the solvents in vacuo. The residue was re-dissolved in MeCN and purified by semi-preparative HPLC

using a Kinetex 5 µm F5 column (isocratic, 50% acetonitrile in 0.1% formic acid) to yield compound **26** (4.5 mg).

Fmoc-Gly-tacca (**26**): white solid; ^{1}H NMR (500 MHz, methanol-*d*$_4$): δ 7.79 (d, J = 7.4 Hz, 2H), 7.65 (d, J = 7.4 Hz, 2H), 7.39 (t, J = 7.4 Hz, 2H), 7.32 (d, J = 7.4 Hz, 2H), 5.27 (dd, J = 11.8, 2.8 Hz, 1H), 5.23 (d, J = 2.8 Hz, 1H), 4.63 (d, J = 5.6 Hz, 1H), 4.40 (m, 2H), 4.32 (m, 1H), 4.27 (m, 1H), 4.24 (d, J = 6.7 Hz, 1H), 3.83 (m, 2H), 3.63 (dd, J = 10.1, 4.4 Hz, 1H), 3.35 (m, 1H), 3.25 (m, 1H), 2.42 (dd, J = 13.4, 10.4 Hz, 1H), 2.25 (t, J = 10.9 Hz, 1H), 2.19 (m, 1H), 2.09 (s, 3H), 2.07 (s, 3H), 1.99 (m, 1H), 1.96 (m, 1H), 1.95 (m, 1H), 1.91 (s, 3H), 1.81 (dd, J = 13.4, 9.6 Hz, 1H), 1.61 (s, 3H), 1.24 (s, 3H), 0.98 (s, 3H), 0.90 (s, 3H), 0.88 (d, J = 6.4 Hz, 3H); ^{13}C NMR (125 MHz, methanol-*d*$_4$): δ 177.3, 173.6, 171.6, 171.5, 159.4, 155.9, 145.2, 145.1, 142.6, 128.9, 128.3, 128.2, 126.2, 126.1, 121.0, 112.0, 80.3, 75.7, 75.2, 73.7, 72.5, 72.4, 68.3, 57.9, 54.3, 54.1, 51.9, 51.4, 49.3, 48.3, 45.7, 45.2, 42.0, 38.3, 36.5, 34.2, 32.2, 26.9, 25.4, 22.1, 21.5, 21.0, 20.7, 20.6, 14.0, 13.5; HRESIMS (m/z): $[M+Na]^+$ calcd. for $C_{51}H_{60}N_2NaO_{15}$, 963.3886; found 963.3892.

Synthesis of Flu-tacca-6 (9)

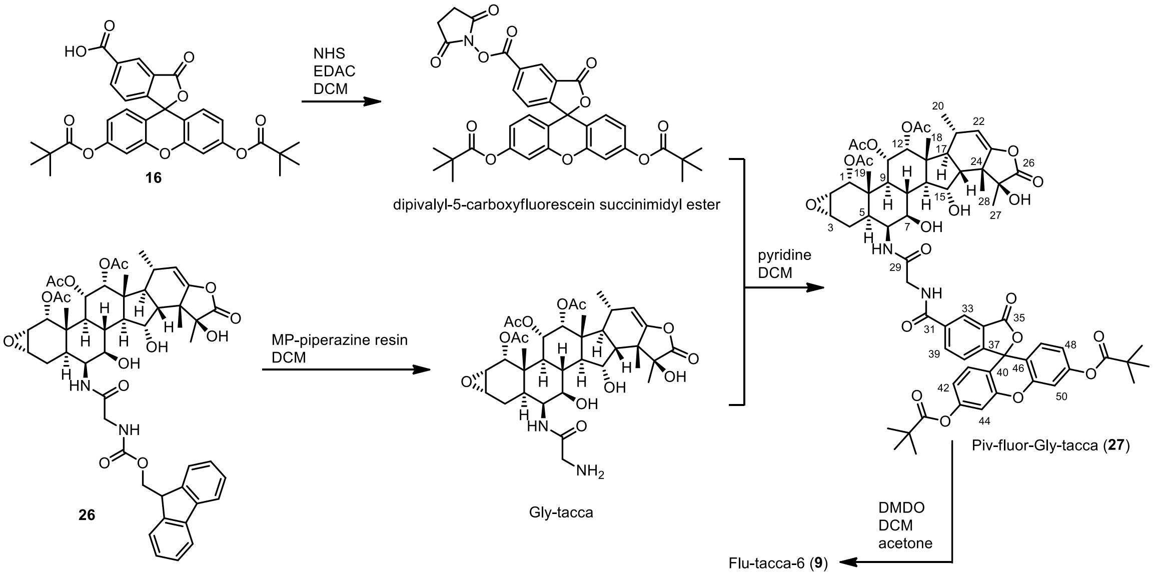

Supplementary Figure 94. Synthesis of Flu-tacca-6 (**9**)

Compound **26** (10 mg) was stirred with MP-piperazine resin (200 mg) in DCM (1 mL) at 40°C for 24 h followed by the removal of the solvent in vacuo. The residue was washed with MeOH (2 mL x 3) and the combined MeOH wash was dried down in vacuo to yield the crude Gly-tacca which was directly mixed with the crude dipivalyl-5-carboxyfluorescein succinimidyl ester (13 mg) (prepared from **16** using the same method as previously described) in 2 mL DCM/pyridine (1 : 1). The reactant mixture was further stirred at 40°C overnight. The solvents were removed in vacuo and the residue was purified by semi-preparative HPLC using a Kinetex 5 µm Biphenyl column (isocratic, 65% MeCN in 0.1% formic acid) to yield compound **27** (3.0 mg).

Piv-fluor-Gly-tacca (**27**): white solid; ^{1}H NMR data, see Supplementary Table 6; ^{13}C NMR (125 MHz, methanol-d_4) (some carbons were not detectable due to

limited amount of material): δ 177.9, 177.3, 172.9, 172.3, 171.6, 171.5, 169.9, 168.8, 157.0, 155.9, 154.4, 152.8, 137.5, 136.0, 130.0, 127.8, 125.7, 125.3, 119.4, 117.0, 112.1, 111.6, 83.4, 80.3, 75.7, 75.3, 73.8, 72.5, 72.4, 57.9, 54.5, 54.1, 51.9, 51.5, 51.3, 49.1, 45.7, 44.8, 42.1, 40.2, 38.4, 36.6, 34.3, 32.2, 27.4, 27.0, 25.4, 22.1, 21.5, 21.0, 20.7, 20.6, 14.1, 13.6; HRESIMS (m/z): $[M+Na]^+$ calcd. for $C_{67}H_{76}N_2NaO_{21}$, 1267.4833; found 1267.4845.

Compound **27** (3.0 mg) was epoxidized in 0.6 mL DMDO-acetone/DCM (1 : 1) solution as previously described. The product was purified by semi-preparative HPLC using a Kinetex 5 µm F5 column (isocratic, 65% MeCN) to yield compound **9** (1.8 mg).

Flu-tacca-6 (**9**): light yellow solid; ^{1}H NMR data, see Supplementary Table 6; HRESIMS (m/z): $[M+Na]^+$ calcd. for $C_{67}H_{76}N_2NaO_{22}$, 1283.4782; found 1283.4718.

Synthesis of Flu-tacca-7 (11)

Compound **14** (10 mg) was stirred with **16** (13 mg, 1.5 equiv) and HATU (5.5 mg, 1.5 equiv) in 2 mL EtOAc/pyridine (1 : 1) at 35°C overnight. The solvents were removed in vacuo and the residue was purified by preparative HPLC using a Kinetex 5 µm F5 column (isocratic, 70% MeCN in 0.1% formic acid) to yield compound **10** (3.8 mg).

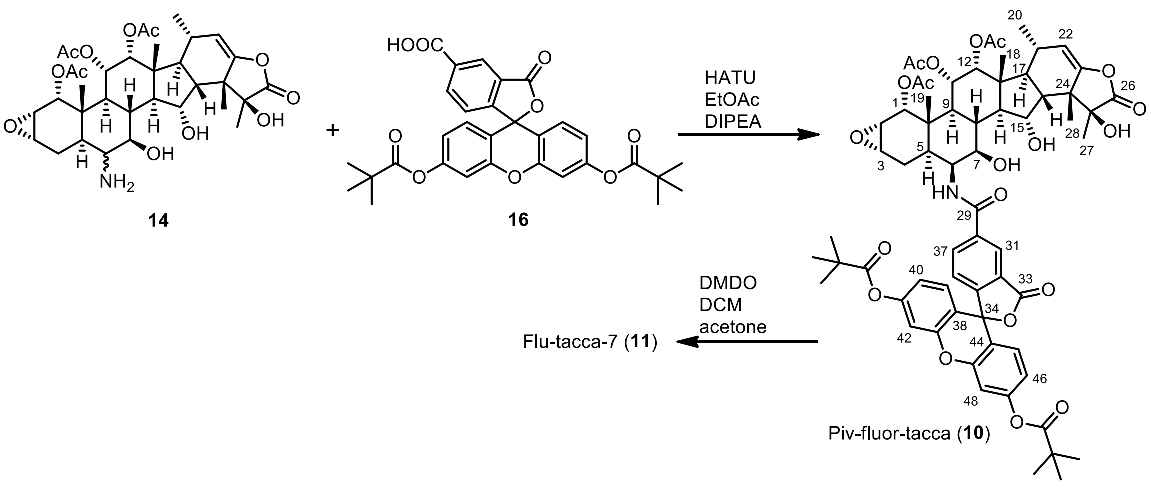

Supplementary Figure 95. Synthesis of Flu-tacca-7 (**11**)

Piv-fluor-tacca (**10**): white solid; ^{1}H NMR data, see Supplementary Table 7; ^{13}C NMR (125 MHz, acetonitrile-*d*$_3$) (some carbons were not detectable due to limited amount of material): δ 177.5, 176.6, 171.4, 171.0, 170.7, 169.1, 156.3, 155.6, 154.0, 152.4, 138.2, 136.2, 130.0, 127.1, 125.4, 125.0, 119.3, 116.9, 111.6, 111.5, 82.5, 80.0, 75.0, 74.6, 74.1, 72.2, 72.0, 57.5, 54.7, 53.4, 51.7, 51.0, 51.7, 48.6, 45.3, 41.6, 39.8, 38.0, 36.5, 33.8, 31.9, 27.3, 26.6, 25.4, 22.4, 21.6, 21.1, 20.8, 20.4, 14.4, 13.7; HRESIMS (m/z): $[M+Na]^+$ calcd. for $C_{65}H_{73}NNaO_{20}$, 1210.4618; found 1210.4676.

Compound **10** (3.0 mg) was epoxidized in 0.6 mL DMDO-acetone/DCM (1 : 1) solution as previously described. The product was purified by semi-preparative HPLC using a Kinetex 5 µm F5 column (isocratic, 70% MeCN) to yield compound **11** (1.4 mg).

Flu-tacca-7 (**11**): light yellow solid; ^{1}H NMR data, see Supplementary Table 7; HRESIMS (m/z): $[M+Na]^+$ calcd. for $C_{65}H_{73}NNaO_{21}$, 1226.4567; found 1226.4578.

Synthesis of Flu-tacca-8 (12)

Compound **14** (9.5 mg) was stirred with **17** (9.9 mg, 1.5 equiv) and HATU (8.2 mg, 1.5 equiv) in 2 mL EtOAc/pyridine (1 : 1) at 35°C overnight. The solvents were removed in vacuo to yield the crude Ac-fluor-tacca which was re-dissolved in 3 mL MeOH/H_2O (2 : 1) followed by the addition of 50 µL ammonia hydroxide ($NH_3 \cdot H_2O$). The mixture was stirred at room temperature for 1 h and then the solvents were removed in vacuo**.** The residue was purified by preparative HPLC using a Kinetex 5 µm F5 column (isocratic, 45% MeCN in 0.1% TFA) to yield compound **28** (7.1 mg).

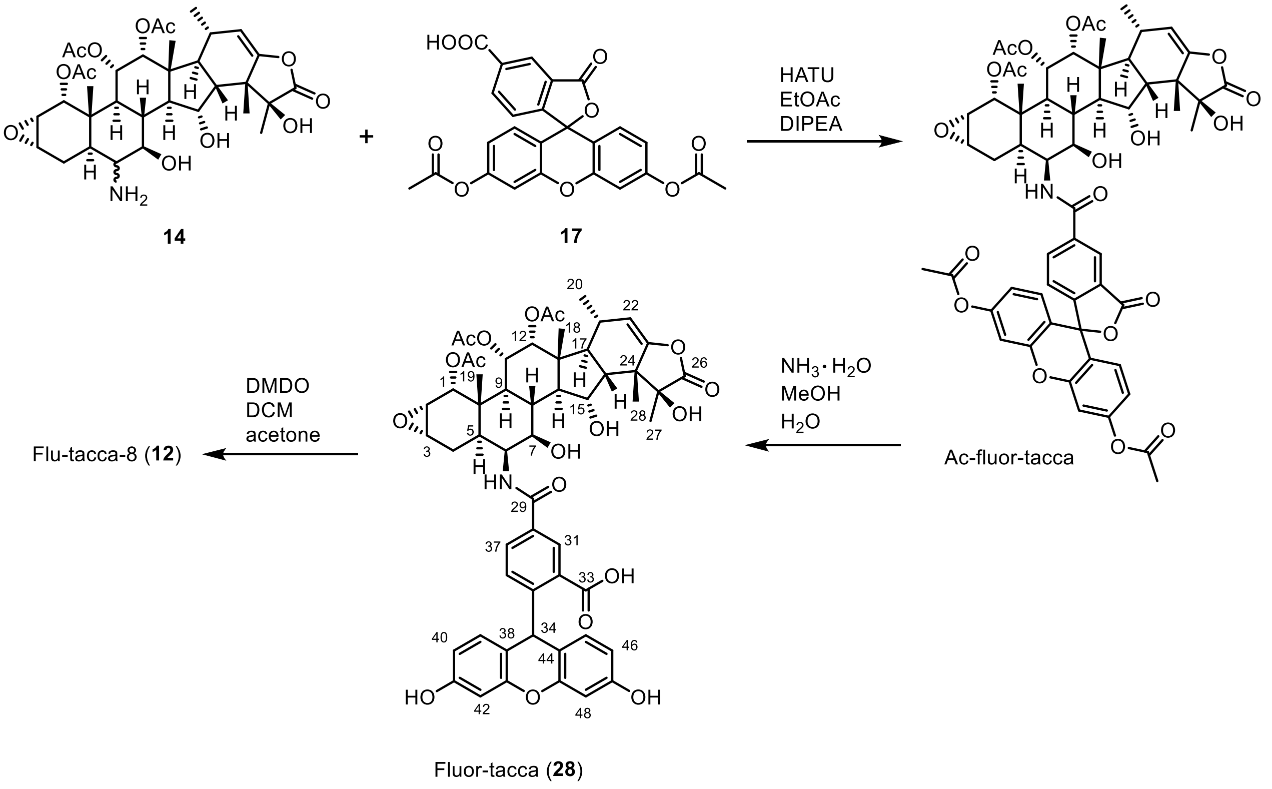

Supplementary Figure 96. Synthesis of Flu-tacca-8 (**12**)

Fluor-tacca (**28**): yellow solid; ^{1}H NMR data, see Supplementary Table 8; HRESIMS (m/z): $[M+Na]^+$ calcd. for $C_{55}H_{57}NNaO_{18}$, 1042.3468; found 1042.3427. Compound **28** (7.0 mg) was epoxidized in 1 mL DMDO-acetone/DCM (1 : 1) solution as previously described. The product was purified by preparative HPLC using a Kinetex 5 µm F5 column (isocratic, 50% MeCN in 0.1% TFA) to yield compound **12** (6.2 mg).

Flu-tacca-8 (**12**): yellow solid; ^{1}H NMR data, see Supplementary Table 8; HRESIMS (m/z): $[M+Na]^+$ calcd. for $C_{55}H_{57}NNaO_{19}$, 1058.3417; found 1058.34176.

Supplementary References

1 Ueno, Y., Jiao, G. S. & Burgess, K. Preparation of 5-and 6-carboxyfluorescein. *Synthesis-Stuttgart*, 2591-2593, doi:10.1055/s-2004-829194 (2004).

2 Oberg, C. T., Carlsson, S., Fillion, E., Leffler, H. & Nilsson, U. J. Efficient and expedient two-step pyranose-retaining fluorescein conjugation of complex reducing oligosaccharides: Galectin oligosaccharide specificity studies in a fluorescence polarization assay. *Bioconjugate Chem* **14**, 1289-1297, doi:10.1021/bc034130j (2003).

3 Sun, W. C., Gee, K. R., Klaubert, D. H. & Haugland, R. P. Synthesis of fluorinated fluoresceins. *J Org Chem* **62**, 6469-6475, doi:DOI 10.1021/jo9706178 (1997).

4 Wu, X. L. *et al.* Synthesis, Spectroscopic Properties, and Biological Applications of Eight Novel Chlorinated Fluorescent Proteins-labeling Probes. *J Fluoresc* **24**, 775-786, doi:10.1007/s10895-014-1351-x (2014).

5 Li, J. *et al.* Potent Taccalonolides, AF and AJ, Inform Significant Structure-Activity Relationships and Tubulin as the Binding Site of These Microtubule Stabilizers. *Journal of the American Chemical Society* **133**, 19064-19067, doi:10.1021/ja209045k (2011).

6 Lee, M. M., Gao, Z. & Peterson, B. R. Synthesis of a Fluorescent Analogue of Paclitaxel That Selectively Binds Microtubules and Sensitively Detects Efflux by P-Glycoprotein. *Angew Chem Int Edit* **56**, 6927-6931, doi:10.1002/anie.201703298 (2017).
