# Supplementary material for: Elucidating target specificity of the taccalonolide covalent microtubule stabilizers employing a combinatorial chemical approach
Source: Nat Commun. 2020 Jan 31;11:654. doi: 10.1038/s41467-019-14277-w (PMC6994698; doi:10.1038/s41467-019-14277-w)
Supplement: Supplementary file 3 — Reporting Summary [file 41467_2019_14277_MOESM3_ESM.pdf]

nature research

Corresponding author(s): April L. Risinger

Last updated by author(s): Dec 6, 2019

Reporting Summary

Nature Research wishes to improve the reproducibility of the work that we publish. This form provides structure for consistency and transparency in reporting. For further information on Nature Research policies, see Authors & Referees and the Editorial Policy Checklist .

Statistics

For all statistical analyses, confirm that the following items are present in the figure legend, table legend, main text, or Methods section.

n/a	Confirmed	
☐	☒	The exact sample size (*n*) for each experimental group/condition, given as a discrete number and unit of measurement
☐	☒	A statement on whether measurements were taken from distinct samples or whether the same sample was measured repeatedly
☐	☒	The statistical test(s) used AND whether they are one- or two-sided<br>*Only common tests should be described solely by name; describe more complex techniques in the Methods section.*
☒	☐	A description of all covariates tested
☐	☒	A description of any assumptions or corrections, such as tests of normality and adjustment for multiple comparisons
☐	☒	A full description of the statistical parameters including central tendency (e.g. means) or other basic estimates (e.g. regression coefficient) AND variation (e.g. standard deviation) or associated estimates of uncertainty (e.g. confidence intervals)
☒	☐	For null hypothesis testing, the test statistic (e.g. *F*, *t*, *r*) with confidence intervals, effect sizes, degrees of freedom and *P* value noted<br>*Give P values as exact values whenever suitable.*
☒	☐	For Bayesian analysis, information on the choice of priors and Markov chain Monte Carlo settings
☒	☐	For hierarchical and complex designs, identification of the appropriate level for tests and full reporting of outcomes
☒	☐	Estimates of effect sizes (e.g. Cohen's *d*, Pearson's *r*), indicating how they were calculated

Our web collection on statistics for biologists contains articles on many of the points above.

Software and code

Policy information about availability of computer code

Data collection	Molecular Devices Softmax (SRB antiproliferative/cytotoxicity data and tubulin polymerization data), PerkinElmer Harmony (high content operetta imaging), Leica Application suite (confocal imaging), LiCOR Image studio (immunoblotting), BioRad CFX Maestro (RT-PCR), VnmrJ 4.0 (Varian NMR), Labsolutions (Shimadzu HPLC and LCMS), Empower 3 (Waters HPLC), MassHunter Workstation (Agilent QTOF), APEX (Bruker X-ray)
Data analysis	Graphpad Prism (biological data analysis), MestReNova 11.0 (NMR), ChemOffice 2016 (2D and 3D structure drawing), MassHunter Workstation (HRESIMS data), Labsolutions (LCMS data), Schrodinger 2018 (docking and modeling)

For manuscripts utilizing custom algorithms or software that are central to the research but not yet described in published literature, software must be made available to editors/reviewers. We strongly encourage code deposition in a community repository (e.g. GitHub). See the Nature Research guidelines for submitting code & software for further information.

Data

Policy information about availability of data

All manuscripts must include a data availability statement. This statement should provide the following information, where applicable:

- Accession codes, unique identifiers, or web links for publicly available datasets
- A list of figures that have associated raw data
- A description of any restrictions on data availability

Provide your data availability statement here.

Field-specific reporting

Please select the one below that is the best fit for your research. If you are not sure, read the appropriate sections before making your selection.

☒ Life sciences ☐ Behavioural & social sciences ☐ Ecological, evolutionary & environmental sciences

For a reference copy of the document with all sections, see nature.com/documents/nr-reporting-summary-flat.pdf

Life sciences study design

All studies must disclose on these points even when the disclosure is negative.

Sample size	Sample sizes of 3 independent experiments for quantitative in vitro experiments and 2 independent experiments for qualitative in vitro results were used, occasionally when more independent experiments were performed, this is noted. The data obtained from these sample sizes has historically been reproducible when additional replicates are performed for these types of studies.
Data exclusions	No data was excluded, when additional experiments were performed, it has been noted
Replication	All data that are described as qualitative were representative of at least 2 independent experiments. All data that are quantified are replicated at least 3 independent times.
Randomization	No randomization was used in these studies as there was no group allocation used in this study
Blinding	No blinding was used in these studies as there was no group allocation used in this study

Reporting for specific materials, systems and methods

We require information from authors about some types of materials, experimental systems and methods used in many studies. Here, indicate whether each material, system or method listed is relevant to your study. If you are not sure if a list item applies to your research, read the appropriate section before selecting a response.

Materials & experimental systems

n/a	Involved in the study	
☐	☒	Antibodies
☐	☒	Eukaryotic cell lines
☒	☐	Palaeontology
☒	☐	Animals and other organisms
☒	☐	Human research participants
☒	☐	Clinical data

Methods

n/a	Involved in the study	
☒	☐	ChIP-seq
☒	☐	Flow cytometry
☒	☐	MRI-based neuroimaging

Antibodies

Antibodies used	β-tubulin at 1:1000 (Sigma T-4026, lot 107M4801V), Texas Red-X at 1:200 (Invitrogen T-862, lot 732914), β-tubulin at 1:1000 (abcam, ab6046, lot GR3204950-1), fluorescein at 1:500 (abcam, ab19491, lot GR3209552-4), IRDye 680 or 800 goat anti-rabbit secondary antibodies at 1:10,000 (LI-COR Biosciences, 680-926-68071, lot C50706-03 & 800-925-32211, lot C70926-01), βIII-tubulin 1:400 (Sigma-Aldrich, T8660) clone SDL.3D10 ascites fluid
Validation	All antibodies were obtained from commercial sources and have been heavily referenced. The antibodies were validated by immunoblotting by molecular weight and by detection of additional higher molecular weight bands with tubulin antibodies only when the tubulin fusion protein was expressed. Similarly, the specificity of the fluorescein antibody was confirmed by presence only when the fluorescein tagged taccalonolide was added to cells. Validation by immunofluorescence was determined by observing microtubule bundling or loss when cells were treated with microtubule stabilizing or destabilizing drugs, respectively.

Eukaryotic cell lines

Policy information about cell lines

Cell line source(s)	HCC1806 (CRL-2335) and HCC1937 (CRL-2336) human triple-negative breast cancer cells, HeLa (CCL-2) cervical cancer cells and SK-OV-3 (HTB-77) ovarian cancer cells were obtained from ATCC (Manassas, VA). SK-OV-3 ovarian cancer cells stably overexpressing Pgp (SK-OV-3/MDR-1-6/6) were obtained from Dr. Susan Kane and subcloned by limiting dilution to isolate the single-cell clones utilized in these studies. A single-cell clone from the transfection of HeLa cells with βIII-tubulin, designated wild type βIII, was constructed and obtained from Dr. Richard Ludueña.

Authentication	Commercial cell lines were validated by STR profiling (Genetica). Pgp and BIII-tubulin expressing lines were verified by RT-PCR and immunoblotting for these resistance markers, respectively.
Mycoplasma contamination	The absence of mycoplasma contamination was determined using the Mycoplasma Detection Kit-Quick Test (Cat: B39032 and Lot: JW004).
Commonly misidentified lines (See ICLAC register)	We have included the use of HeLa cells in our analysis for several reasons. One reason is that it allows for the direct comparison of the in vitro potency as compared to other compounds of this class, which have been predominantly reported in this line. However, we have also evaluated these effects in three additional cancer cell lines and obtained consistent relative potencies of our compounds in all four lines. We also utilized HeLa cells for our transfection experiments where we ectopically expressed tubulin mutants due to the high degree of transfectability of this cell line and the historic use of this line for these type of studies. This line was additionally validated by STR profiling (Genetica).
